# Design and synthesis of new indole drug candidates to treat Alzheimer’s disease and targeting neuro-inflammation using a multi-target-directed ligand (MTDL) strategy

**DOI:** 10.1080/14756366.2022.2126464

**Published:** 2022-09-22

**Authors:** Phoebe F. Lamie, Maha M. Abdel-Fattah, John N. Philoppes

**Affiliations:** a Department of Pharmaceutical Organic Chemistry, Faculty of Pharmacy, Beni-Suef University, Beni-Suef, Egypt; b Department of Pharmacology and Toxicology, Faculty of Pharmacy, Beni-Suef University, Beni-Suef, Egypt

**Keywords:** Indole, Alzheimer’s disease, inflammation, AChE, BuChE

## Abstract

A novel series of indole-based compounds was designed, synthesised, and evaluated as anti-Alzheimer’s and anti-neuroinflammatory agents. The designed compounds were *in vitro* evaluated for their AChE and BuChE inhibitory activities. The obtained results revealed that compound **3c** had higher selectivity for AChE than BuChE, while, **4a**, **4b,** and **4d** showed selectivity for BuChE over AChE. Compounds **5b**, **6b**, **7c,** and **10b** exerted dual AChE/BuChE inhibitory activities at nanomolar range. Compounds **5b** and **6b** had the ability to inhibit the self-induced Aβ amyloid aggregation. Different anti-inflammatory mediators (NO, COX-2, IL-1β, and TNF-α) were assessed for compounds **5b** and **6b**. Cytotoxic effect of **5b** and **6b** against human neuroblastoma (SH-SY5Y) and normal hepatic (THLE2) cell lines was screened *in vitro*. Molecular docking study inside *rh*AChE and *h*BuChE active sites, drug-likeness, and ADMET prediction were performed.

## Graphical Abstract

**Figure:**
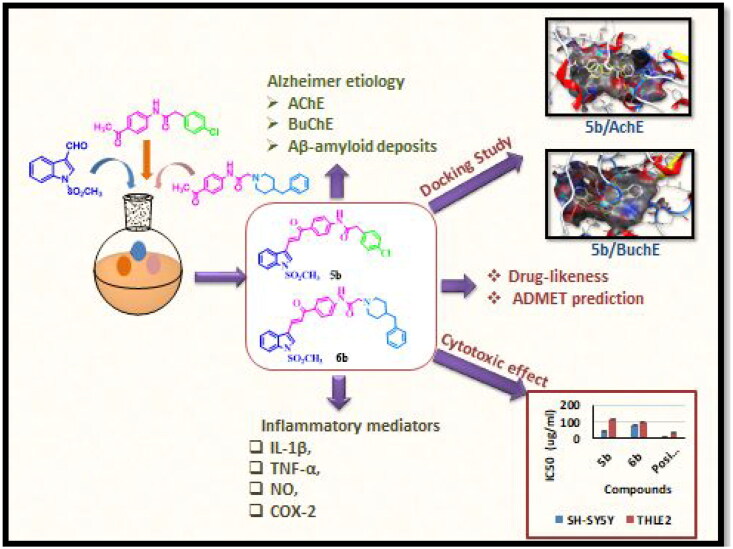

## Introduction

The most common form of a chronic irreversible neurodegenerative disorder is Alzheimer’s disease (AD). It is characterised by memory deterioration, loss of speech, cognitive impairment in elderly people^1^^,^^2^. It has been reported that 36 million people in the world were living with dementia in 2010, and every 20 years, this number will double, resulting in increasing the number of people with AD to be more than 152 million people by the end of 2050. It is expected that AD people will cost about US $2 trillion by 2030^3^^,^^4^.

Searching the literature, the aetiology of AD is not completely known, but the most characteristic pathogens of this multifactorial disease are low levels of acetyl choline, β-amyloid (Aβ) deposits, *tau*-protein (ƭ) aggregation, oxidative stress, and biometals dyshomeostasis^5–7^. The casual role in AD is arises from inflammation. Thus, the characteristic feature of AD is chronic and sustained microglia activation which results in increasing inflammatory mediators, such as cyclooxygenase-2 (COX-2), nitric oxide (NO), tumour necrosis factor α (TNF-α), and interleukin 1B (IL-1B). These mediators lead to neuronal apoptosis and facilitate the propagation of a neuro-inflammation detrimental cycle^3^^,^^5^.

Until now, there has been no drug to cure AD. The most common known FDA-approved therapeutic agents are acetyl cholinesterase inhibitors (AChEIs), namely, tacrine, rivastigmine, and galantamine^2^^,^^3^^,^^7^. They counteract the action of choline estrases (ChEs), such as acetylcholinesterase (AChE) and butyrylcholinesterase (BuChE) in hydrolysis of the neurotransmitter acetylcholine into choline and acetic acid^8–10^. Moreover, the most effective drug for treating AD is donepezil (**I**), but it is effective for a short period of time and then the symptoms are reversed^11^.

Recently, compounds containing piperazinyl pyrimidine scaffold were reported to have anti-neuroinflammatory activity. Compound GIBH-130 (**II**)-approved by China Food and Drug Administration for clinical trials against AD – can suppress IL-1β production selectively in nano molar concentration with IC_50_ = 3.4 nM^12^. Different anti-inflammatory drugs, such as indomethacine (**III**) – with indole ring as a main core – and rofecoxib (**IV**), a selective COX-2 inhibitor, have been reported^5^^,^^13–17^.

Other polyphenolic natural products with stilbene chemical structural features have also been known. Resveratrol (**V**) and Ferulic acid (**VI**) have various therapeutic activities, especially as antioxidant, anti-Aβ-aggregation, and anti-inflammatory agents^6^^,^^18^. Thus resveratrol (**V**) was reported to suppress the activation of NF-ƙB and as a result, it could inhibit COX-2 enzyme and retain anti-inflammatory properties^19^. Moreover, butin (**VIII**) is a natural product that contains chalcone part, was reported to modulate neurodegenerative disorders^20^^,^^21^.

Furthermore, it was observed that introducing certain moieties such as hydrazine (compound **VIII**), amide linkage, thiazole ring (compound **IX**), or indole scaffold to AD drugs, increased their activity through their choline esterase inhibitory activity, anti-Aβ-aggregation properties, or anti-neuroinflammatory character^22–27^.

Guided by the above facts, and due to problems of most clinical AD drugs, such as nausea, vomiting, diarrhoea, and nephrotoxicity^28–30^, there is an urgent need to apply the multi-target-directed-ligand (MTDL) strategy “one molecule, multiple targets” to design and synthesis new drug candidates that can interact with multiple targets involved in the pathogenesis of AD.

**Figure 1.**
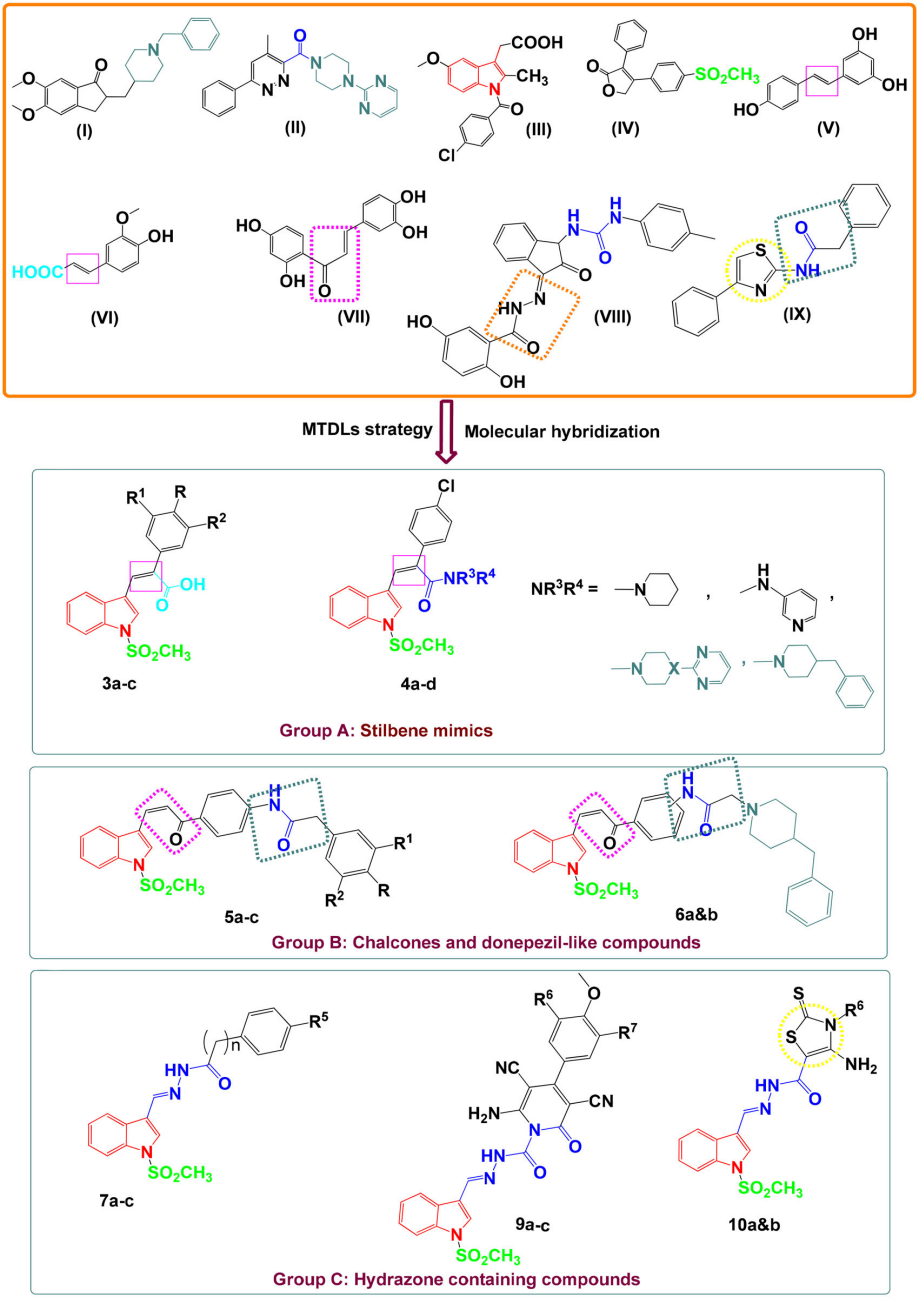
Examples of some drug candidates and natural products for the treatment of AD and anti-inflammatory agents and the design strategy for the novel derivatives.

In our design, we kept in mind the chemical structure and biological activity of donepezil (**I**) (AchEI), GIBH-130 (**II**) (anti-neuroinflammatory agent), indole-containing anti-inflammatory drug indomethacin (**III**), COX-2 selective drug celecoxib (**IV**), and anti-inflammatory and antioxidant stilbene derivatives (**V–VII**) (Figure 1).

The designed compounds were classified into three groups A–C (Figure 1) to contain an indole ring as a main core and decorated by methanesulfonyl group (SO_2_Me) as a selective COX-2 pharmacophore, besides:

- Stilben moiety [as in resveratrol (**V**) and ferulic acid (**VI**)] – group A
- Piperazinyl pyrimidine moiety and other secondary amines [to mimic GIBH-130 (**II**)] – group A
- Benzyl piperidine ring [to resemble donepezil (**I**)] – groups A and B
- Chalcone part and –NHCOCH_2_-linker in some prepared derivatives [for anti-inflammatory activity as in butein (**VIII**) and compound **IX**] – group B
- Hydrazone moiety and thiazole scaffold [as in compounds **VIII** and **IX**] – group C

The synthesised compounds were subjected to: spectroscopic analysis (IR, ^1^H NMR, Dept-Q NMR, and Mass) and elemental analysis to confirm the chemical structures, measurement of their AChE and BuChE inhibitory activities to evaluate their effect on AD, assessment of antineuro-inflammatory activity through measurement of NO, COX-2, IL-1β, and TNF-α, cytotoxic effect on human neuroblastoma (SH-SY5Y) and normal hepatic (THLE2) cell lines. Moreover, molecular docking studies and ADMET prediction were investigated.

## Results and discussion

### Chemistry

**Scheme 1.**
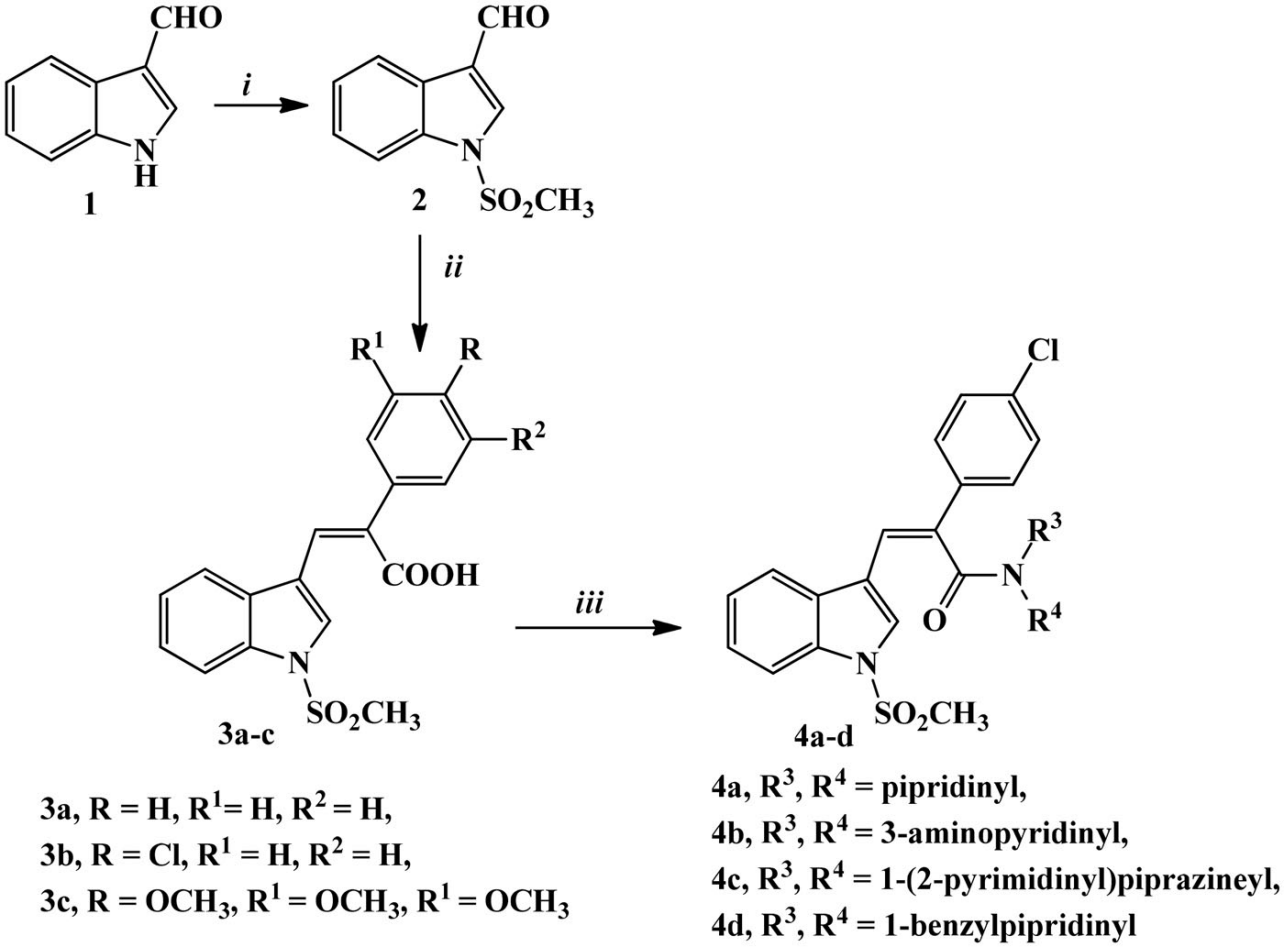
Synthetic routes for preparation of starting material **2**, carboxylic acid derivatives **3a–c**, and amide derivatives **4a–d**. Reagents and conditions: (i) NaH, ClSO_3_H, THF, stirring R.T., 3 h; (ii) phenyl acetic acid, p-chlorophenyl acetic acid or 3,4,5-trimethoxyphenyl acetic acid, K_2_CO_3_, Ac_2_O, 90 °C, 4–6 h; (iii) the appropriate amine, HBTU, DMF, stirring 2–4 h.

**Scheme 2.**
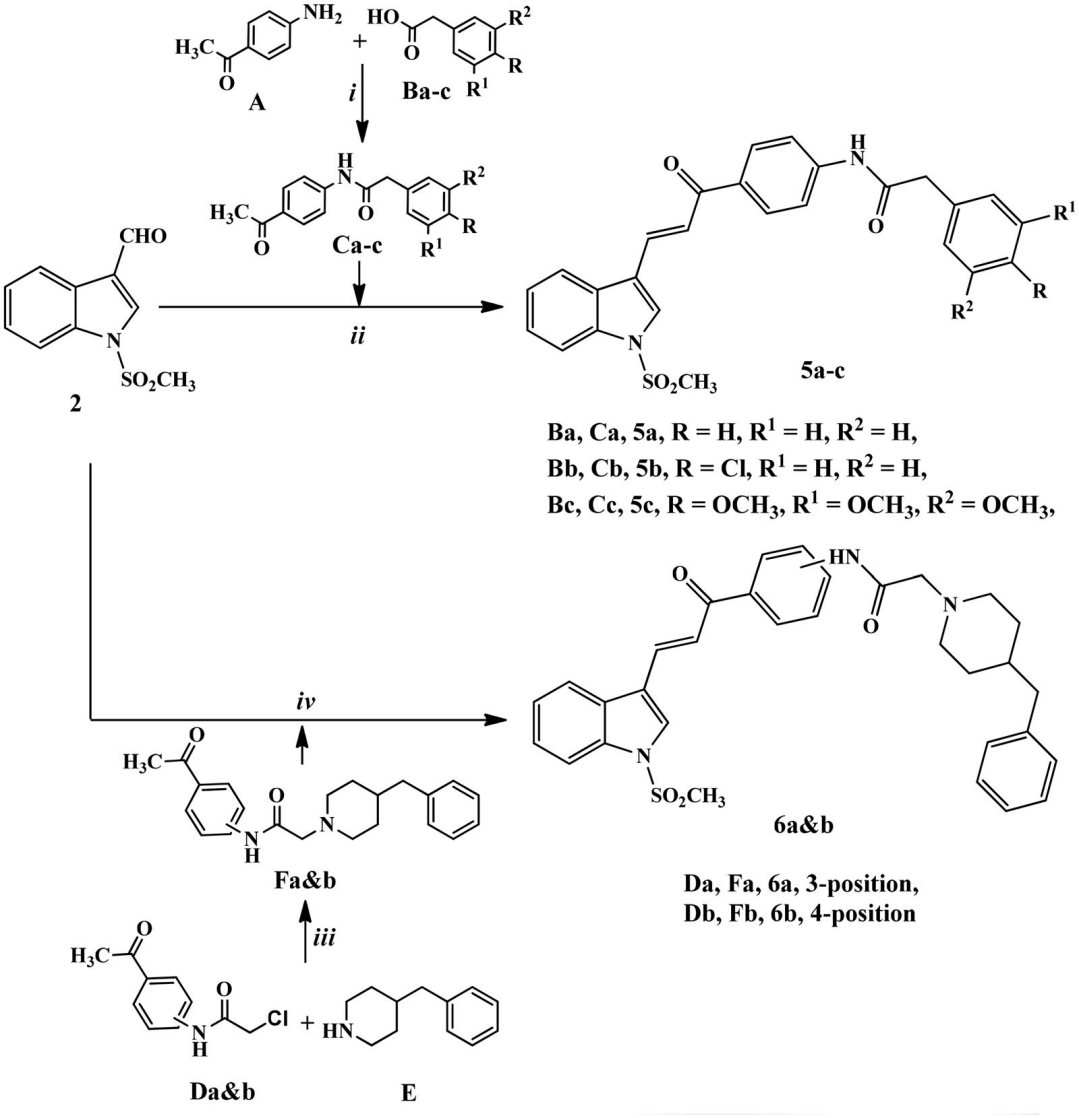
Synthetic routes for preparation of chalcone derivatives **5a–c** and **6a,b**. Reagents and conditions: (i) HBTU, DMF, stirring 8 h; (ii) NaOEt, EtOH abs., stirring R.T., 24 h; (iii) K_2_CO_3_, KI, acetone, reflux, 6–8 h; iv) KOH, MeOH, stirring R.T., 24 h.

**Scheme 3.**
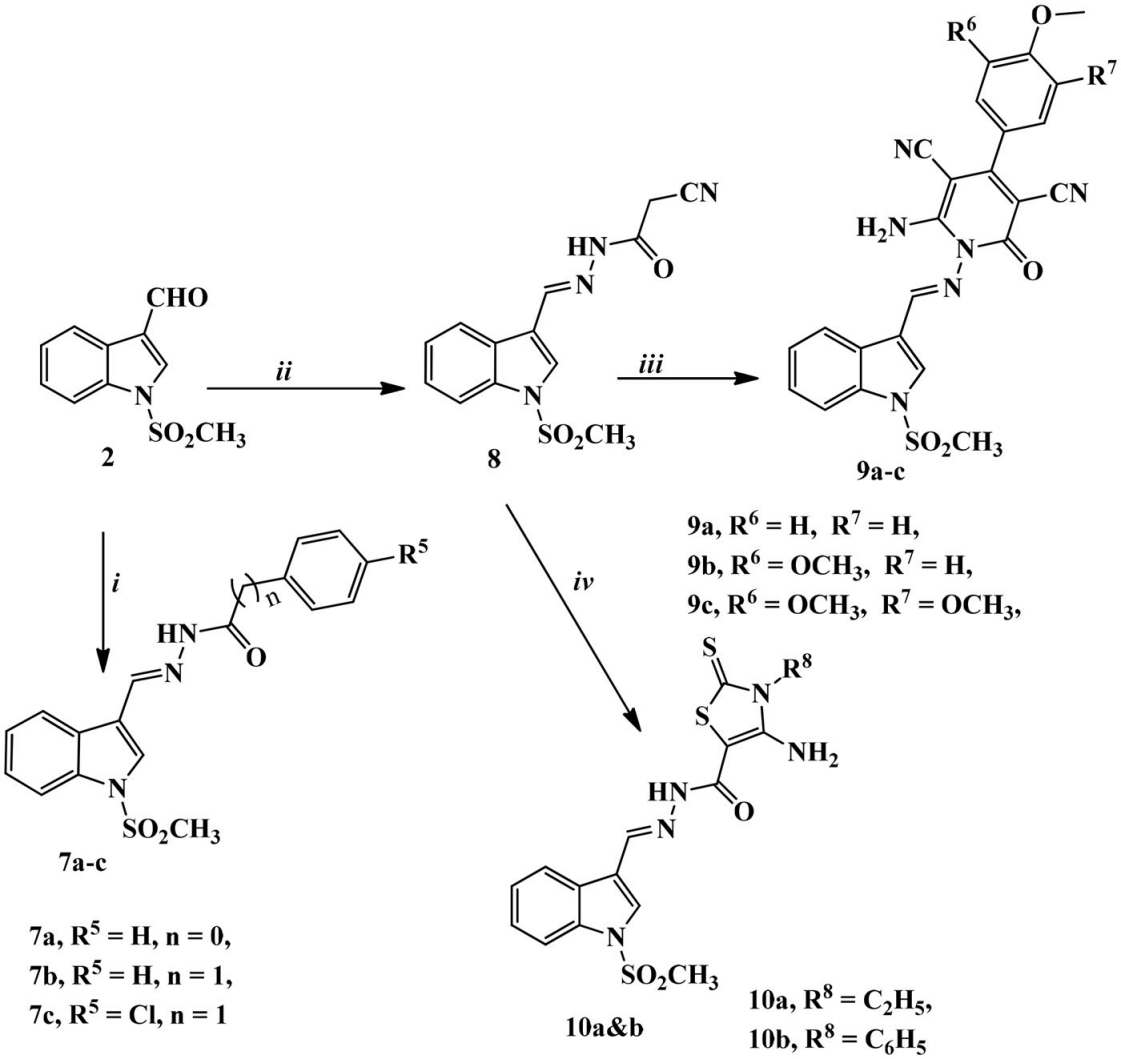
Synthetic routes for preparation of imine derivatives **7a–c**, **8**, **9a–c,** and **10a,b**. Reagents and conditions: (i) benzohydrazide, phenylacetic acid hydrazide, or *p*-chlorophenyl acetic acid hydrazide, gl. acetic acid, reflux 3–5 h; (ii) cyanoacetic acid hydrazide, abs. ethanol, reflux 3 h; (iii) the appropriate arylidine derivative, abs. EtOH, reflux 4–6 h; (iv) ethyl/or phenyl isothiocyanate, S, abs. EtOH, TEA, reflux 10–12 h.

Synthetic routes for the development of novel *N*-methylsulfonyl indole derivatives have been outlined in Schemes 1–3.

In Scheme 1, novel stilbene mimic derivatives **3a–c** were synthesised through perkin reaction^31^ by condensation of 3-indole carboxaldehyde derivative **2**^32^ and commercially available phenylacetic acid, *p*-chlorophenyl acetic acid, or 3,4,5-trimethoxyphenyl acetic acid in acetic anhydride and potassium carbonate. The produced compounds **3a–c** were subjected to a condensation reaction with different secondary aliphatic amines, such as piperidine, 1–(2-pyrimidinyl)piperazine, and 1-benzylpipridine, or with primary aromatic amine, 3-aminopyridine, using HBTU (reagent used specifically for amidic bond formation), and DMF as a solvent to obtain amidic derivatives **4a–d**. The yields ranged from 73 to 79%.

In Scheme 2, chalcone derivatives **5a–c** and **6a,b** were outlined. Reaction of *p*-aminoacetophenone (**A**) with different phenyl acetic acid derivatives **Ba–c,** afforded the key intermediates, **Ca–c**. By applying Claisen–Schmidt condensation reaction conditions, compounds **Ca–c** reacted with indole carboxaldehyde derivative **2** in absolute ethanol using sodium ethoxide to give derivatives **5a–c** for good to high yields.

*N*-Chloroacetyl derivatives of 3- or 4-aminoacetophenone **Da,b** were heated under reflux temperature with benzyl pipridine (**E**) in acetone in the presence of potassium carbonate and catalytic amount of KI to afford precursors **Fa,b** in excellent yields through nucleophilic substitution reaction.

Finally, the target compounds **6a,b** were generated by stirring at room temperature compound **2** with the intermediates **Fa,b** in methanol containing KOH for 24 h.

In Scheme 3, we tried to introduce hydrazone moiety to the prepared compounds. Thus, indole carboxaldehyde derivative **2** underwent a condensation reaction with benzohydrazide, phenylacetohydrazide, or *p*-chlorophenylacetohydrazide in glacial acetic acid to give indole molecules **7a–c**.

The key intermediate **8** was obtained from the reaction of compound **2** with cyanoacetic acid hydrazide under reflux conditions using absolute ethanol as a solvent. Then, the target derivatives **9a–c** and **10a,b** were afforded by reacting compound **8** with either different arylidene derivatives giving pyridine-containing compounds **9a–c** or with ethyl/or phenyl isothiocyanate derivatives, elemental sulphur in absolute ethanol containing catalytic amount of triethylamine to produce **10a,b**.

The structures of the synthesised compounds were confirmed with the help of IR, ^1^H, and ^13^C NMR and mass spectral data (see Experimental part).

### Biological evaluation

#### Acetylcholinesterase and butyrylcholinesterase (AChE and BuChE) inhibition activity results

**Figure 2.**
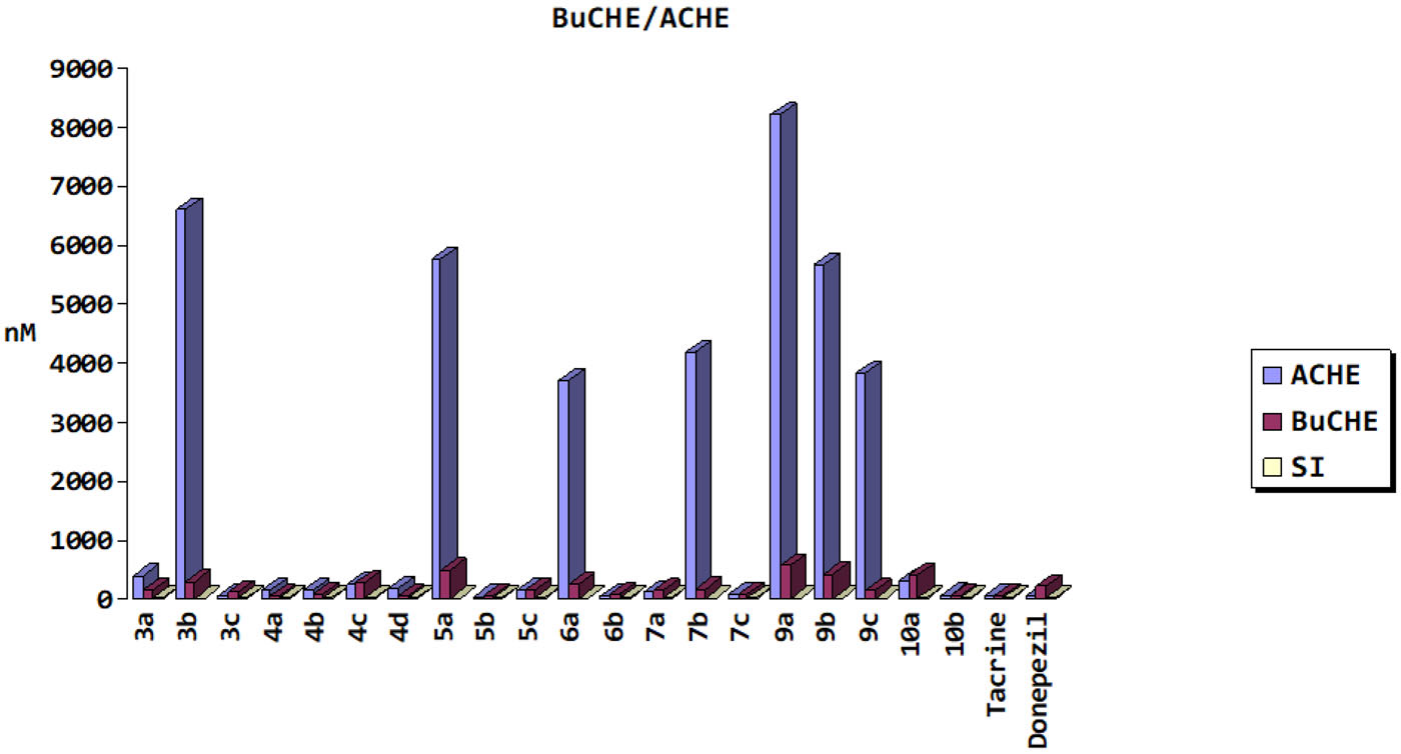
Acetylcholinesterase and butyrylcholinesterase (AChE and BuChE) inhibition activities for synthesised target compounds and reference drugs.

The effects of twenty synthetic compounds were evaluated for AChE and BuChE inhibition using the modified method of Ellman et al.^33^ Inhibitory activities were detected and results were expressed as IC_50_ (nM) values (Figure 2). The results of the assay showed that almost all compounds were moderate to strong inhibitors of AChE except **3b**, **4c**, **5a**, **6a**, **7 b**, **9a**, **10a,** and **9b** with less activity as compared to reference drugs (tacrine and donepezil). On the other side, *p*-chlorophenyl chalcone derivative **5b** exhibited the strongest AChE inhibitory activity (IC_50_ = 27.54 nM), which was better than that of the reference compounds, donepezil (IC_50_ = 55.39 nM), and tacrine (IC_50_ = 38.57 nM). As well as exhibiting the strongest inhibitory effect on BuChE (IC_50_ = 36.85 nM) as compared to reference donepezil (IC_50_ = 219.36 nM) and similar to that of tacrine activity (IC_50_ = 35.95 nM). Additionally, *p*-benzylpipridine chalcone derivative, **6b,** had AChE inhibitory effect (IC_50_ = 49.30 nM) slightly less than tacrine and donepezil, while more potent as BuChE inhibitor (IC_50_ = 80.44 nM) than reference donepezil and slightly less active than tacrine. Moreover, hydrazine-containing compounds, **7c** and **10b**, exerted dual AChE/BuChE inhibitory activities with IC_50_ values 89.12, 64.54 nM against AChE and 75.96, 47.40 nM for BuChE enzymes, sequentially. Also, it was observed that stilbene carboxylic acid derivative, **3c**, had AChE inhibitory activity with IC_50_ = 41.11 nM, while converting carboxylic acid to amide bond in derivatives **4a**, **4b,** and **4d** showed inhibitory activity against BuChE enzyme with IC_50_ ranged from 41.68 to 74.06 nM more than AChE inhibitory activity. AChE and BuChE are found in the brains of mammals. Genetically, structurally, and kinetically, the two kinds vary. In the human brain, BuChE is found in glial cells and neurons as well as in plaques and tangles in AD patients^34^. Also, it was reported that AChE activity decreases progressively in the brain of AD patients, BuChE activity shows some increase. BuChE may replace AChE by hydrolysing brain acetylcholine in some conditions, such as in mice nullizygote for AChE or in AD patients in advanced stages of the disease^34^^,^^35^. Based on the mentioned information, **5ub** and **6b** compounds may be used in both cases, mild and advanced AD cases.

#### Inhibition of Aβ1-42 self-induced aggregation

AD is a chronic irreversible neurodegenerative disease. It is caused by accumulation of amyloid plaques in the brains of patients suffering from AD^36^. Plaques are mainly composed of Beta-amyloid (Aβ) peptides: Aβ1-40 and Aβ1-42. Recent studies showed that two Beta-Amyloid peptides (Aβ1-40 and Aβ1-42) exist in brain tissues, cerebrospinal fluid (CSF), and plasma in patients suffering from AD. In particular, aggregated Aβ1-42 is considered a validated biomarker for diagnosing AD^37^.

Tacrine as a reference drug was used in this study to evaluate the inhibitory activity of eight selected compounds on Aβ1-42 aggregation. The compounds showed strong activity (IC_50_ = 5.16–22.40 µM) as compared to tacrine (IC_50_ = 3.50 µM) as indicated in Table 1. Interestingly, compound **5b** showed more potent inhibitory activity (IC_50_ = 2.50 µM) as compared to tacrine. As well as compound **6b** showed inhibitory activity (IC_50_ = 4.94 µM) nearly similar to tacrine (Table 1).

**Table 1.** Values of inhibition of Aβ1–42 self-induced aggregation for tested compounds and tacrine.

Compounds	IC_50_ (uM)
**3c**	6.91
**4a**	22.40
**4b**	13.75
**4d**	8.76
**5b**	2.50
**6b**	4.94
**7c**	14.92
**10b**	5.16
**Tacrine**	3.50

#### Nitric oxide (NO) assessment

**Figure 3.**
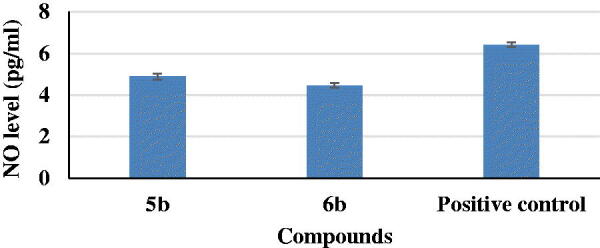
Nitric oxide (NO) assessment for compounds **5b** and **6b** and a positive control.

In AD, increased production of vascular NO, a highly neurotoxic mediator in the CNS, may contribute to the vulnerability of neurons to injury and cell death^38^. Anti-neuroinflammatory activities of the most potent two compounds, **5b** and **6b**, were evaluated on production of NO in LPS-induced BV2 microglia cell lines. It was observed that compounds **5b** and **6b** induced a decrease in NO level (4.89 and 4.46 pg/mL, respectively) if compared to a positive control (6.42 pg/mL) (Figure 3).

#### Cyclooxygenase-2 (COX-2) assessment

**Figure 4.**
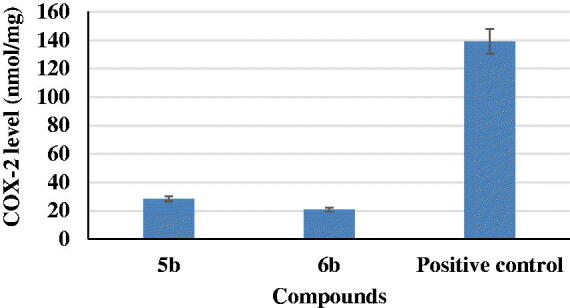
Cyclooxygenase-2 (COX-2) assessment for compounds **5b** and **6b** and a positive control.

The two most potent compounds, **5b** and **6b**, were evaluated on production of COX-2 in LPS-induced BV2 microglia cell lines. Previously, it was reported that COX-2 is a key mediator in the inflammatory response and may play a role in neurodegeneration^39^. The results of this study revealed that compounds **5b** and **6b** induced a decrease in COX-2 levels of about 20 and 14%, respectively, as compared to a positive control (Figure 4).

#### Interleukin-1β (IL-1β) assessment

**Figure 5.**
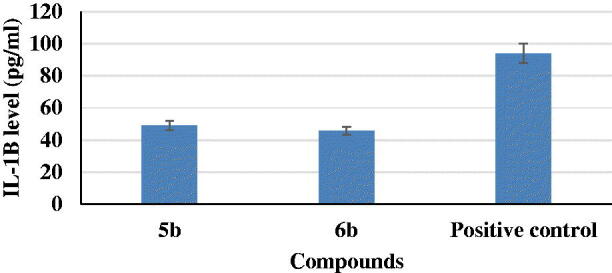
Interleukin-1β (IL-1β) assessment for compounds **5b** and **6b** and a positive control.

IL-1β is a pro-inflammatory cytokines involved in the pathogenesis of AD^40^. So, activity of **5b** and **6b** derivatives were evaluated against LPS-induced BV2 microglia cell lines production of IL-1β. They induced a decrease in IL-1β level to about 5% and 48%, sequentially, as compared to a positive control (Figure 5).

#### Tumour necrosis factor-α (TNF- α) assessment

**Figure 6.**
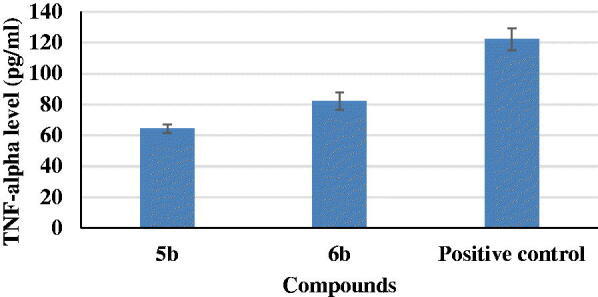
Tumour necrosis factor-α (TNF-α) assessment for compounds **5b** and **6b** and positive control.

TNF-α is a pro-inflammatory cytokine that has been demonstrated to have a key role in inflammation. TNF-α signalling exacerbates both Aβ and *tau* pathologies *in vivo*, according to several lines of evidence based on genetic and pharmacological modifications. Anti-inflammatory therapies, both preventive and interventional, were found to reduce brain damage and improve cognitive function in rodent models of AD. In this work, results revealed that compounds **5b** and **6b** showed a remarkable decrease in TNF-α levels to 53 and 67% if compared to positive control (Figure 6).

#### Cytotoxicity of synthetic compounds in SH-SY5Y and THLE2 cells

**Figure 7.**
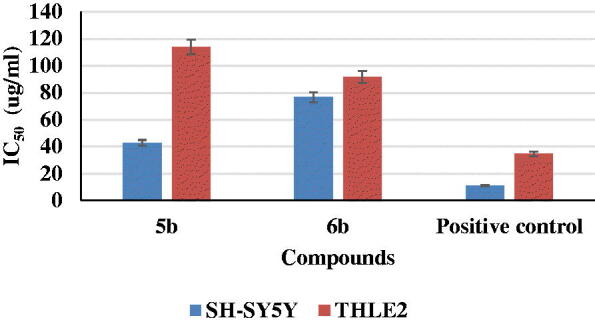
Cytotoxicity of synthetic compounds **5b** and **6b** and positive control on SH-SY5Y and THLE2 human cell lines.

An MTT assay was performed to investigate the effect of the selected two compounds, **5b** and **6b,** on cell viability using human neuroblastoma (SH-SY5Y) and normal hepatic (THLE2) cell lines. The cells were treated with compounds **5b** and **6b**, evaluated the cytotoxicity in comparison with staurosporine (IC_50_ = 11.1 µg/mL) for SH-SY5Y, as **5b** had an IC_50_ value 42.8 µg/mL and IC_50_ value of 76.6 µg/mL for compound **6b**. As well as staurosporine, which had IC_50_ = 34.6 µg/mL for THLE2 cells, compounds **5b** and **6b** exerted IC_50_ values equal to 114 and 91.5 µg/mL, respectively (Figure 7).

### Docking studies on AChE and BuChE

To explore the possible binding mode of the tested compounds and justify their potency, compounds **3c**, **5b**, **6b**, **7c**, and **10b**, as the most active AChEIs among all test compounds (IC_50_ = 27.54–89.12 nM), were selected and docked into recombinant human acetylcholinesterase (*rh*AChE) active site. Moreover, **4a**, **4b**, **4d**, **5b**, **6b**, **7c,** and **10b** derivatives, most active as BuChEIs (IC_50_ = 36.85–80.44 nM), were chosen for docking into human butrylcholinesterase (*h*BuChE) active site. A molecular docking study was conducted using Molecular Operating Environment software (MOE 2014.0901).

The X-ray crystallographic structures of both *rh*AChE in complex with donepezil (PDB: 4EY7) and *h*BuChE in complex with inden-naphthamide derivative (PDB: 4TPK) were obtained from a protein data bank.

Concerning docking studies inside the *rh*AChE active site, donepezil, the ligand compound, forms three binding interactions with the *rh*AChE active site. Thus, its dimethoxyphenyl ring formed an arene–arene interaction with Trp286 amino acid. Both –CH_2_– of piperidinyl moiety and the phenyl group of benzyl part could interact with Tyr341 and Trp86 amino acids through arene-H and arene-arene interactions, respectively. Its binding energy score was −17.2793 Kcal/mol.

By inspecting docking results of tested compounds **3c**, **5b**, **6b**, **7c,** and **10b**, it was found that their binding energy scores ranged from −31.6883 to −17.0177 Kcal/mol.

Moreover, they form binding interactions with Trp86, Tyr341, and Trp286 amino acids, the same as donepezil, *via* arene-arene and arene-H interactions, in addition to H-binding interactions with Tyr124, Ph225, Gly448, and His447 amino acids.

Thus, the binding mode of the most active AChE inhibitor, chalcone derivative **5b** (IC_50_ = 27.54 nM), showed two hydrogen-bonding interactions between SO_2_Me/Trp124 amino acid and C=O/Ph225 amino acid. Moreover, the other chalcone derivative **6b**, with –NHCOCH_2_– linker and benzylpipridine pharmacophore, exerted two arene–arene interactions between the phenyl ring of the benzyl part and that of –NHPh with Trp86 and Trp286 amino acids, respectively, beside arene-H interaction between –CH_2_– alkyl part of benzyl moiety and Tyr341 amino acid. Additionally, compound **7c**, bearing –NHCOCH_2_– spacer, displayed arene-H interaction between the –SO_2_Me moiety and Trp286 amino acid. Moreover, –SO_2_Me pharmacophore of compound **3c**, trimethoxy stilbene derivative, interacted with Trp86 amino acid through arene-H binding mode.

From the thiazole series, compound **10b** interacted with Tyr341 and His447 amino acids through arene-H binding mode with both pyrrole and thiazole rings, respectively. It also exerted arene-arene interaction between pyrrole/Trp286 amino acid and a Hydrogen-bonding interaction with Gly448 amino acid.

Regarding the BuChE active site, it was found that the ligand compound reacts with the *h*BuChE active site through arene-arene interaction between –CH_2_– group/Trp82 amino acid and arene-cation interaction between piperidine-NH/Tyr332 amino acid. Additionally, Hydrogen-bonding interaction between the C=O group and Gly116 amino acid was observed. Ligand Energy-binding score was −16.4403 Kcal/mol.

For tested derivatives, the most active one, **5b** (IC_50_ = 36.85 nM), had an energy-binding score of −19.5216 Kcal/mol. It formed three arene-arene interactions between phenyl ring/Trp82, pyrrole ring/Trp82 and –CO–Ph ring/Tyr332 amino acid, the same as was observed in the case of ligand docking study. Additionally, stilbene derivatives containing pipridine ring **4a** or benzyl piperidine moiety **4d**, with energy-binding scores of −17.9970 and −11.4498 Kcal/mol, sequentially, displayed arene-H interactions with Trp82 and Gly116 amino acids.

Thiazole derivative **10 b** showed higher binding affinity than the ligand for *h*BuChE. It formed a hydrophobic interaction with Trp82 amino acid. It was noticed that the pyridine ring in **4b** and *p*-chlorophenyl scaffold in **7c** were responsible for the hydrophobic interactions inside the *h*BuChE active site with Thr120 and Trp82 amino acids, sequentially.

Compound **6b** showed extra interactions with the *h*BuChE active site. It formed binding interactions with Trp82, Il69, and Gln71 amino acids.

From the above data analysis, we can conclude that hydrophobic interactions are mainly responsible for the binding process inside both *rh*AChE and *h*BuChE active sites. The most important pharmacophores for activity are benzyl, phenyl, *p*-chlorophenyl, pyridine, pyrrole, thiazole, piperidine rings, besides, –SO_2_Me, C=O, C=S, –CH_2_–, and –NHCOCH_2_– moieties.

Moreover, compounds **5b**, **6b**, **7c,** and **10b** showed dual AChE/BuChE inhibitory activities. They were shared in –NHCOCH_2_– group in **5b**, **6b,** and **7c** and thiazole ring in **10b**.

**Figure 8.**
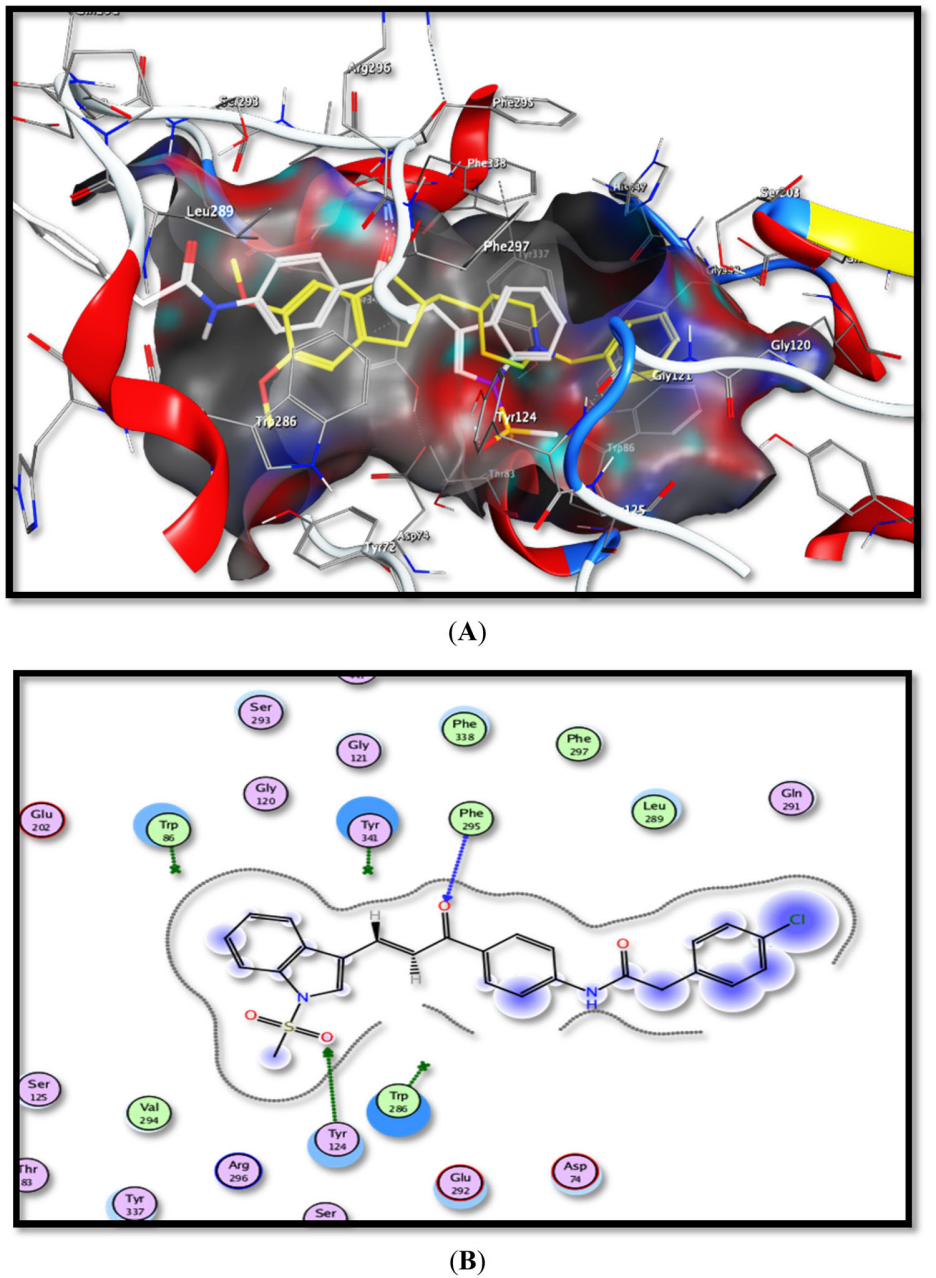
Binding interactions of the most active compound **5b** inside *rh*AChE active site, (A) 3D image, **5b** is described as white colour line and the ligand as yellow colour line, (B) 2D image.

**Figure 9.**
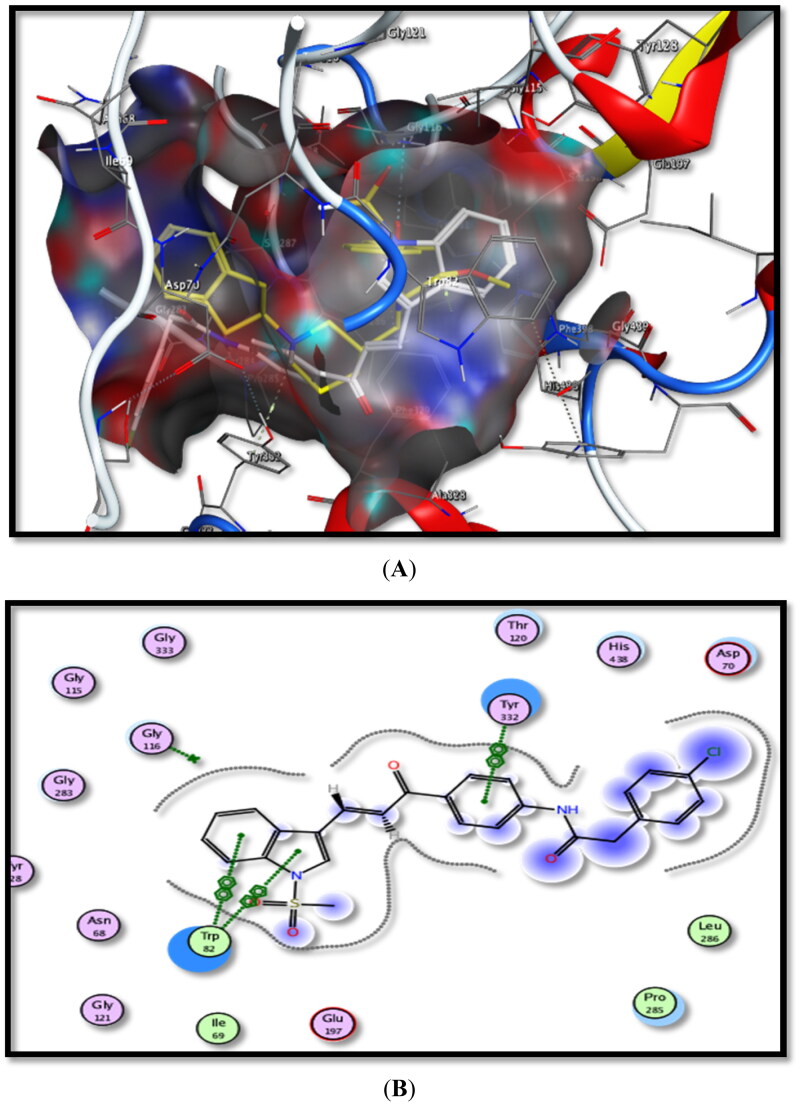
Binding interactions of the most active compound **5b** inside *h*BuChE active site, (A) 3D image, **5b** is described as white colour line and ligand as yellow colour line, (**B**) 2D image.

The obtained data are summarised in Table 2 and the schematic binding modes (2D and 3D) are depicted in Figures 8 and 9.

**Table 2.** Molecular modelling data for selected test compounds and ligands into *rh*AChE and *h*BuChE active sites.

Compd. No.	AChE			BuChE		
	Energy score (Kcal/mol)	Binding interaction	Amino acid	Energy score (Kcal/mol)	Binding interaction	Amino acid
**3c**	−17.4815	–SO_2_Me arene-H	Trp86	–	–	–
**4a**	–	–	–	−17.9970	*p*-Cl-Ph arene-H *p*-Cl-Ph arene-H	Trp82 Gly116
**4b**	–	–	–	−17.3823	Pyridine arene-H	Thr120
**4d**	–	–	–	−19.5390	Indole-Ph arene-H	Trp82
**5b**	−31.6883	–SO_2_Me –CH = CH–CO–	Tyr124 Phe225	−19.5216	Ph arene-arene Pyrrol arene-arene CO-Ph arene-arene	Trp82 Trp82 Tyr332
**6b**	−28.3927	–CH_2_–Ph arene-arene Pipridine–CH_2_– arene-H –NH–Ph arene-arene	Trp86 Tyr341 Trp286	−12.9466	-CH_2_-Ph arene-H Pipridine CH_2_ arene-H SO_2_Me arene-H SO_2_Me	Trp82 Trp82 Ile69 Gln71
**7c**	−17.0177	SO_2_Me arene-H	Trp286	−17.4498	*p*-Cl-Ph arene-arene	Trp82
**10b**	−18.4817	Pyrrole arene-H Pyrrole arene-arene Thiazole arene-H C=S	Tyr341 Trp286 His447 Gly448	−20.7428	Indole-Ph arene-H	Trp82
**Ligand/ AChE**	−17.2793	Dimethoxyphenyl arene-arene Pipridine–CH_2_– arene-H –CH_2_–Ph arene-arene	Trp286 Tyr341 Trp86	–	–	–
**Ligand/ BuChE**	–	–	–	−16.4403	C=O –CH_2_–OMe Pipridine-NH+ arene-cation	Gly116 Trp82 Tyr332

### ADME study

#### Predicted physicochemical properties and drug-likeness

To explore drug-likeness properties of the most active derivatives, such as AChE and BuChE inhibitors, **3c**, **4a**, **4b**, **4d**, **5b**, **6b**, **7c,** and **10 b** compared with donepezil and tacrine drugs, theoretical calculations, such as molecular weight (MW), the number of hydrogen-bond acceptors and donors, number of rotable bonds, TPSA, percentage of absorption as well as lipophilic indicator log*p* “octanol/water” partition coefficient were evaluated (Table 3).

**Table 3.** Predicted physicochemical properties and drug-likeness for some active compounds, donepezil and tacrine.

Compd. no.	^a^MW	^b^nON	^c^nOHNH	^d^logP (o/w)	^e^nrotb	^f^TPSA	^g^%Abs	*n* violation
Acceptable value	˂500	˂10	˂5	˂5	≤10	˂160	100%	≤1
**3c**	431.47	8	1	2.29	7	104.08	73.10	0
**4a**	442.97	5	0	4.35	4	59.38	88.52	0
**4b**	451.94	6	1	3.82	5	81.07	81.04	0
**4d**	533.09	5	0	6.02	6	59.38	88.52	2
**5b**	492.98	6	1	4.88	7	85.25	79.59	0
**6b**	555.70	7	1	5.38	9	88.48	78.48	2
**7c**	389.86	6	1	3.46	5	80.54	81.22	0
**10b**	471.59	8	3	2.60	5	111.50	70.54	0
**Donepezil**	379.50	4	0	4.10	6	38.78	95.63	0
**Tacrine**	198.27	2	2	3.05	0	38.91	95.58	0

^a^Molecular weight, ^b^number of hydrogen-bond acceptors, ^c^number of hydrogen-bond donors, ^d^octanol/water partition coefficient,^ e^number of rotable bonds, ^f^topological polar surface area, ^g^absorbtion (%).

Missing more than one of Lipinski’s parameters may be attributed to bioavailability problems in the target compounds as drugs predicted.

The obtained results showed that six of eight tested compounds, stilbene derivatives **3c**, **4a**, **4b**, chalcone derivative **5b**, hydrazone derivative **7c** and thiazole derivative **10b**, obeyed Lipinski’s rule with no violation and may meet the criteria for orally active drugs. They had a similar drug-likeness to donepezil and tacrine.

Compounds **4d** and **6b** had slightly increased MW, over 500, besides having low membrane permeability with octanol/water partition coefficient of 6.02 and 5.38, respectively, more than the acceptable value.

#### In silico ADME prediction

Pharmacokinetic properties, such as absorption, distribution, metabolism, and excretion of the most active derivatives **3c**, **4a**, **4b**, **4d**, **5b**, **6b**, **7c,** and **10b** were determined using *in silico* ADME properties prediction. The results were compared to donepezil and tacrine drugs. As shown in Table 4, all the target derivatives showed high intestinal absorption values ranging from 96.61% to 99.78%, which are nearer to those of reference drugs, donepezil (97.95%) and tacrine (96.51%).

**Table 4.** *In silico* ADME prediction results for some active compounds, donepezil and tacrine.

Compd. no.	^a^HIA	^b^CaCo-2	^c^MDCK	^d^PPB	^e^BBB	^f^SkinP	
Acceptable value	70–100%	>90		>90%	>0.40	≥ −3	Solubility mg/L
**3c**	99.78	3.74	0.04	100	0.07	–1.78	5.07
**4a**	97.77	19.12	0.04	92.73	0.02	–1.66	0.39
**4b**	97.30	10.86	0.04	95.71	0.01	–2.13	0.48
**4d**	98.26	21.50	0.04	90.70	0.09	–1.59	0.01
**5b**	97.47	10.29	0.06	93.68	0.21	–2.04	0.01
**6b**	97.52	17.47	0.43	96.63	0.22	–1.95	0.01
**7c**	96.61	2.07	0.07	100.00	0.01	–2.15	1.13
**10b**	98.00	0.60	0.04	100.00	0.05	–2.44	0.00
**Donepezil**	97.95	55.51	0.13	84.61	0.187	–3.04	6.23
**Tacrine**	96.51	25.85	38.45	95.39	0.86	–3.04	41.09

^a^Human intestinal absorbtion (%), ^b^*in vitro* CaCo cell permeability (nm/sec), ^c^*in vitro* MDCK cell permeability (nm/s), ^d^*in vitro* plasma protein binding (%), ^e^*in vitro* blood–brain barrier penetration (C. brain/C. blood), and ^f^Skin permeability.

Permeability for *in-vitro* CaCo-2 cells was in the low to moderate range (0.60 − 21.50).

Additionally, low permeability values for *in-vitro* MDCK cells in the range 0.04 − 0.43 were noticed.

Plasma protein binding (PPB) scores for tested derivatives (90.73–96.63%) were close to that of donepezil (84.61%) and tacrine (95.39%) except for **3c**, **7c,** and **10b** derivatives which exerted those strong bound effect reached to 100%.

Moreover, chalcone derivatives **5b** and **6b** had higher absorption into the CNS than donepezil. Their predicted blood–brain barrier (BBB) values were 0.21 and 0.22, sequentially, while that of donepezil was 0.18.

Lower skin permeability (SP) properties ranging from −1.59 to −2.04 were observed for all tested derivatives than those of reference drugs (−3.04). On the other hand, solubility in pure water for trimethoxy stilbene derivative **3c** was 5.07 mg/L, close to that of donepezil 6 mg/L. From the above results, we conclude that tested compounds, especially, **3c**, **4a,** and **5b** have good ADME properties and can be further optimised for durability.

#### Predicted toxicity properties

To predict the toxicity properties of the most active derivatives **3c**, **4a**, **4d**, **5b,** and **6b**, the AMES test and carcino-Mouse/Rat were measured. Additionally, cardiac toxicity of the selected compounds was checked *via* hERG-inhibition. Standard drugs, donepezil and tacrine were used to compare the obtained results (Table 5).

**Table 5.** Predicted toxicity properties results for some active compounds, donepezil and tacrine.

Compd. no.	AMES	Carcino-Mouse	Carcino-Rat	hERG-inhibition
**3c**	Mutagen	Negative	Negative	Medium-risk
**4a**	Non-mutagen	Negative	Negative	Medium-risk
**4b**	Non-mutagen	Negative	Negative	Medium-risk
**4d**	Non-mutagen	Negative	Negative	Medium-risk
**5b**	Non-mutagen	Positive	Negative	Medium-risk
**6b**	Non-mutagen	Negative	Negative	Medium-risk
**7c**	Mutagen	Negative	Negative	Low-risk
**10b**	Non-mutagen	Positive	Negative	Ambiguous
**Donepezil**	Mutagen	Negative	Negative	Medium-risk
**Tacrine**	Mutagen	Positive	Negative	Medium-risk

Results showed that trimethoxy stilbene derivative, **3c** and hydrazine derivative **7c**, resemble to donepezil in its mutagenic behaviour in the AMES test and had negative carcinogenic effect in mice and rats, besides its medium- to low-risk effect as a cardiotoxic agent. On the other hand, chalcone derivative, **5b** and thiazole analogue **10b**, exerted similar effects on tacrine as being positive carcinogenic in mice and negative in rats and still having medium-risk or ambiguous behaviour as a cardiotoxic agent. They were differing in being non-mutagenic in AMES test. All other tested derivatives **4a**, **4b**, **4d,** and **6b** showed non-mutagenic effects in the AMES test, negative carcinogenic behaviour in mice and rats and medium-risk as cardiotoxic agents. From the above results, it was justified that the target derivatives may have good characters as lead drugs.

#### Metabolism prediction

*In silico* phase I metabolism study can explore inhibitors to cytochrome P450 isoforms, such as CYP1A2, CYP2C19, CYP2C9, CYP2D6, and CYP3A4 properties and predict excretion property of the target compounds through measuring P-glycoprotein (P-gp) substrates. Thus, all tested derivatives, **3c**, **4a**, **4b**, **4d**, **5b**, **6b**, **7c,** and **10b** were similar to donepezil and tacrine in that they could not inhibit CYP1A2 and CYP2D6 isoforms, respectively. While they could act as inhibitors to CYP2C9 and CYP3A4, except **3c** and **7c**, did not show any inhibitory activity on CYP3A4 isoform. On the other hand, only two derivatives, **4d** and **6b** could not act as CYP2C19 inhibitors mimic the action of both donepezil and tacrine. Regarding P-gp, two of the tested compounds **4d** and **6b** were considered as P-gp substrates (Table 6).

**Table 6.** Metabolism prediction results for some active compounds, donepezil and tacrine.

Compd. No.	CYP1A2 inhibitor	CYP2C19 inhibitor	CYP2C9 inhibitor	CYP2D6 inhibitor	CYP3A4 inhibitor	P-gp substrate
**3c**	No	Yes	Yes	No	No	No
**4a**	No	Yes	Yes	No	Yes	No
**4b**	No	Yes	Yes	No	Yes	No
**4d**	No	No	Yes	No	Yes	Yes
**5b**	No	Yes	Yes	No	Yes	No
**6b**	No	No	Yes	No	Yes	Yes
**7c**	No	Yes	Yes	No	No	No
**10b**	No	Yes	Yes	No	Yes	No
**Donepezil**	No	No	No	Yes	Yes	Yes
**Tacrine**	Yes	No	No	No	Yes	Yes

## Conclusion

A new series of indole-based compounds were designed and synthesised as potent anti-Alzheimer’s and anti-neuroinflammatory agents. All the prepared compounds were *in vitro* evaluated for their AChE and BuChE inhibitory activities. The obtained results revealed that stilbene carboxylic acid derivative, **3c**, possessed higher AChEI activity (IC_50_ = 41.11 nM) than BuChEI activity (IC_50_ = 117 nM). While, stilbene amide derivatives, **4a**, **4b,** and **4d**, showed BuChEI activity (IC_50_ = 41.68 − 74.06 nM) over AChEI activity (IC_50_ = 132.20 − 163.80 nM). Chalcone derivatives **5b** and **6b** and hydrazone derivatives **7c** and **10b** showed dual AChE/BuChE inhibitory activities with the IC_50_ range (27.54 − 89.12 nM and 36.85 − 80.44 nM, respectively). All the active compounds were further evaluated for their self-induced Aβ-amyloid aggregation. Compounds **5b** and **6b** were the most potent with IC_50_ = 2.50 and 4.94 µM, sequentially, if compared to tacrine (IC_50_ = 3.5 µM). Different anti-inflammatory mediators, such as NO, COX-2, IL-1β, and TNF-α were assessed for compounds **5b** and **6b** showing higher anti-inflammatory activity than positive control. Additionally, compounds **5b** and **6b** exhibited low toxicity on neuroblastoma (SH-SY5Y) and normal hepatic (THLE2) cell lines. To explore the binding mode of active compounds, derivatives **3c**, **5b**, **6b**, **7c,** and **10b** were docked inside the AChE active site, while, **4a**, **4b**, **4d**, **6b**, **7c,** and **10b** were chosen for docking into the BuChE active site. Data analysis showed that hydrophobic interactions were mainly responsible for the binding process beside H-bonding interactions with some important amino acids such as Trp86, Trp286, Tyr124, Tyr341, Phe225, His447, and Gly448 for AChE, and Trp82, Tyr332, Gly116, Thr120, Ile69, and Gln71 amino acids for BuChE. Finally, from drug-likeness and ADMET prediction results, it was found that six of eight tested compounds (stilbene derivatives **3c**, **4a**, **4b**, chalcone derivative **5b,** and hydrazone containing compounds **7c** and **10b**) obeyed Lipinski’s rule of five and were considered as good candidates for further optimisation to develop new anti-Alzheimer/anti-neuroinflammatory drugs.

## Experimental

### Chemistry

For determination of melting points, a Griffin apparatus was used without correction. Moreover, on a Shimadzu IR-435 spectrophotometer, Infra-red spectra (IR) were recorded, using KBr discs, and values were represented in cm^−1^ (Faculty of Pharmacy, Cairo University). Both ^1^H NMR and ^13^C NMR (DEPT-Q) were carried out using the Bruker instrument at 400 MHz for ^1^H NMR and 100 MHz for ^13^C NMR spectrophotometer (Faculty of Pharmacy, Beni-Suef University, Beni-Suef, Egypt and Faculty of Pharmacy, Mansoura University, Mansoura, Egypt), in DMSO-*d_6_*, D_2_O using TMS as an internal standard and chemical shifts were recorded in ppm on the *δ* scale using DMSO-*d_6_* (2.5) as a solvent. Coupling constant (*J*) values were estimated at Hertz (Hz). Splitting patterns are designated as follows: s, singlet; d, doublet, t, triplet; q, quartette; dd, doublet of doublet; m, multiplet. A Hewlett Packard 5988 spectrometer (Palo Alto, CA), the device that was used for recording the electron impact (EI) mass spectra. C, H, N microanalysis was performed on Perkin-Elmer 2400 at the Microanalytical Centre, Cairo University, Egypt and was within + 0.4% of theoretical values. To follow the course of reactions and to check the purity of final products, analytical thin-layer chromatography (TLC) (pre-coated plastic sheets, 0.2 mm silica gel with UV indicator [Macherey-Nagel]) was employed. All other reagents and solvents were purchased from the Aldrich Chemical Company (Milwaukee, WI) and, were used without further purification.

#### General procedure for the synthesis of compounds 3a–c

Indole carboxaldehyde derivative **2** (0.01 mol, 2.23 g), the appropriate phenylacetic acid derivative (0.01 mol), and potassium carbonate (0.01 mol, 1.38 g) were dissolved in acetic anhydride (5 mL). The mixture was stirred at 90 °C for 4–6 h (monitored by TLC). Water (10 mL) was added and the reaction mixture was stirred at 60 °C for 1 h. The reaction mixture was cooled and acidified with 12 N HCl. The aqueous solution was extracted with CH_2_Cl_2_ (3 × 10 mL), and the obtained organic layers were combined and evaporated to dryness. The formed residue was crystallised from EtOAc to give compounds **3a–c**.

#### (E)-3-[1-(Methylsulfonyl)-1H-indol-3-yl]-2-phenylacrylic acid (3a)

Light brown solid; 81%; mp 181–183 °C; IR (KBr) 3429 (OH), 3028 (CH-aromatic), 2926 (CH-aliphatic), 1671 (C=O), 1363, 1167 (SO_2_) cm^−1^; ^1^H NMR (400 MHz, DMSO-*d_6_*, *δ* = ppm) *δ* = 3.42 (s, 3H, SO_2_CH_3_), 7.26–7.44 (m, 3H, phenyl H-2, H-6 and indole H-6), 7.45–7.47 (m, 5H, phenyl H-3, H-4, H-5 and indole H-5, H-7), 7.73–7.98 (m, 2H, indole H-4 and =CH), 8.23 (s, 1 H, indole H-2), 12.61 (s, 1H, OH, D_2_O exchangeable); ^13^C NMR (100 MHz, DMSO-*d_6_*, *δ* = ppm) *δ* = 41.10, 113.37, 115.72, 119.90, 124.14, 125.80, 126.93, 128.41, 129.15, 129.39, 129.51, 129.75, 133.65, 133.98, 137.36, 168.54; Anal.Calcd for C_18_H_15_NO_4_S (341.38): C, 63.33; H, 4.43; N, 4.10. Found: C, 63.49; H, 4.25; N, 4.13.

#### (E)-2–(4-Chlorophenyl)-3-[1-(methylsulfonyl)-1H-indol-3-yl]acrylic acid (3b)

*Light brown solid; 83%;* mp 232–234 °C; IR (KBr) 3432 (OH), 3025 (CH-aromatic), 2927 (CH-aliphatic), 1669 (C=O), 1331, 1168 (SO_2_) cm^−1^; ^1^H NMR (400 MHz, DMSO-*d_6_*, *δ* = ppm) *δ* = 3.41 (s, 3H, SO_2_CH_3_), 6.72 (s, 1H, =CH), 7.30–7.37 (m, 3H, *p*-chlorophenyl H-2, H-6 and indole H-6), 7.44 (t, 1H, *J* = 7.6 Hz, indole H-5), 7.53 (d, 2H, *J* = 8 Hz, *p*-chlorophenyl H-3, H-5), 7.71 (d, 1H, *J* = 7.6 Hz, indole H-4), 7.80 (d, 1H, *J* = 8 Hz, indole H-7), 8.00 (s, 1 H, indole H-2), 12.84 (s, 1H, OH, D_2_O exchangeable); ^13^C NMR (100 MHz, DMSO-*d_6_*, *δ* = ppm) *δ* = 41.57, 113.41, 115.52, 120.06, 124.15, 125.82, 127.08, 129.37, 129.53, 129.74, 131.67, 132.38, 133.12, 134.10, 136.21, 168.09; EIMS (*m/z*) 376.63 (M + 1, 17.16%), 375.63 (M^+.^, 16.99%), 374.96 (M-1, 11.83%), 130.48 (100%). Anal.Calcd for C_18_H_14_ClNO_4_S (375.83): C, 57.52; H, 3.75; N, 3.73. Found: C, 57.67; H, 3.55; N, 3.91.

#### (E)-3-[1-(Methylsulfonyl)-1H-indol-3-yl]-2–(3,4,5-trimethoxyphenyl)acrylic acid (3c)

Light brown solid; 79%; mp 229–231 °C; IR (KBr) 3428 (OH), 3025 (CH-aromatic), 2932 (CH-aliphatic), 1670 (C=O), 1364, 1173 (SO_2_) cm^−1^; ^1^H NMR (400 MHz, DMSO-*d_6_*, *δ* = ppm) *δ* = 3.27 (s, 3H, SO_2_CH_3_), 3.72 (s, 3H, OCH_3_), 3.80 (s, 6H, 2OCH_3_), 6.52 (s, 2H, trimethoxyphenyl H-5, H-6), 7.31–7.44 (m, 2H, indole H-5, H-6), 7.76–7.82 (m, 3H, indole H-4, H-7 and = CH), 7.98 (s, 1H, indole H-2), 12.71 (s, 1H, OH, D_2_O exchangeable); ^13^C NMR (100 MHz, DMSO-*d_6_*, *δ* = ppm) *δ* = 41.59, 56.53, 60.62, 106.11, 113.05, 114.26, 115.55, 119.34, 122.78, 123.71, 125.63, 126.57, 129.07, 131.29, 133.85, 137.58, 153.67, 164.66; Anal.Calcd for C_21_H_21_NO_7_S (431.46): C, 58.46; H, 4.91; N, 3.25. Found: C, 58.43; H, 5.07; N, 3.17.

#### General procedure for the synthesis of compounds 4a–d

A mixture of compound **3b** (0.001 mol, 0.37 g) with *N*,*N*,*N*',*N*'-tetramethyl-*O*-(1*H*-benzotriazol-1-yl)uranium hexafluorophosphate (HBTU) (0.001 mol, 0.37 g) in dimethylformamide (2 mL) was stirred for 30 min at room temperature. Then, the appropriate amine (0.001 mol) and a catalytic amount of triethylamine were added. The reaction mixture was stirred for 2–4 h at room temperature (monitored by TLC). Water (10 mL) was added. The product was extracted using ethyl acetate. The combined extract was concentrated. The obtained crude compound was crystallised from 95% ethanol to give a pure form of the desired compounds **4a–d**.

#### (E)-2–(4-Chlorophenyl)-3-[1-(methylsulfonyl)-1H-indol-3-yl]-1-(pipridin-1-yl)prop-2-en-1-one (4a)

Brown solid; 74%; mp 121–123 °C; IR (KBr) 3025 (CH-aromatic), 2929 (CH-aliphatic), 1658 (C=O), 1367, 1170 (SO_2_) cm^−1^; ^1^H NMR (400 MHz, DMSO-*d_6_*, *δ* = ppm) *δ* = 1.25–1.59 (m, 6H, 3 CH_2_), 3.44 (s, 3H, SO_2_CH_3_), 3.60 (t, 4H, *J* = 7.6 Hz, N(CH_2_)_2_), 7.10 (s, 1H, =CH), 7.24–7.29 (m, 2H, indole H-5, H-6), 7.35–7.41 (m, 3H, *p*-chlorophenyl H-2, H-6 and indole H-7), 7.45–7.52 (m, 3H, *p*-chlorophenyl H-3, H-5 and indole H-2), 7.77 (d, 1H, J = 7.6 Hz, indole H-4); ^13^C NMR (100 MHz, DMSO-*d_6_*, *δ* = ppm) *δ* = 23.79, 25.18, 41.41, 48.06, 110.83, 113.36, 119.62, 120.91, 123.41, 124.36, 125.32, 128.12, 129.45, 130.55, 135.43, 137.92, 138.86, 142.37, 171.32; Anal.Calcd for C_23_H_23_ClN_2_O_3_S (442.96): C, 62.36; H, 5.23; N, 6.32. Found: C, 62.49; H, 5.15; N, 6.36.

#### (E)-2–(4-Chlorophenyl)-3-[1-(methylsulfonyl)-1H-indol-3-yl]-N-(pyridin-3-yl)acrylamide (4b)

*Yellow crystals; 76%;* mp 187–189 °C; IR (KBr) 3329 (NH), 3026 (CH-aromatic), 2925 (CH-aliphatic), 1708 (C=O), 1366, 1169 (SO_2_) cm^−1^; ^1^H NMR (400 MHz, DMSO-*d_6_*, *δ* = ppm) *δ* = 3.40 (s, 3H, SO_2_CH_3_), 6.72 (s, 1H, *=*CH), 7.30–4.44 (m, 5H, pyridine H-5, indole H-5, H-6 and *p*-chlorophenyl H-3, H-5), 7.51–7.55 (m, 3 H, indole H-7 and *p*-chlorophenyl H-2, H-6), 7.72–7.81 (m, 4 H, pyridine H-4, H-6 and indole H-2, H-4), 8.00 (s, 1H, pyridine H-2), 10.17 (s, 1H, NH, D_2_O exchangeable); ^13^C NMR (100 MHz, DMSO-*d_6_*, *δ* = ppm) *δ* = 41.56, 109.94, 113.18, 115.52, 120.05, 124.15, 125.82, 127.02, 127.84, 129.26, 129.37, 129.74, 131.66, 131.82, 132.26, 134.10, 135.80, 136.18, 141.74, 144.86, 168.19; EIMS (*m/z*) 452.66 (M + 1, 18.37%), 340.73 (100%). Anal.Calcd for C_23_H_18_ClN_3_O_3_S (451.93): C, 61.13; H, 4.01; N, 9.30. Found: C, 60.89; H, 3.80; N, 9.15.

#### (E)-2–(4-Chlorophenyl)-3-[1-(methylsulfonyl)-1H-indol-3-yl]-1-[4-(pyrimidin-2-yl)piprazin-1-yl]prop-2-en-1-one (4c)

White-off solid; 73%; mp 109–111 °C; IR (KBr) 3028 (CH-aromatic), 2923 (CH-aliphatic), 1630 (C=O), 1361, 1169 (SO_2_) cm^−1^; ^1^H NMR (400 MHz, DMSO-*d_6_*, *δ* = ppm) *δ* = 3.43–3.46 (m, 7H, SO_2_CH_3_ and N(CH_2_)_2_), 3.64–3.75 (m, 4H, CON(CH_2_)_2_), 7.01 (t, 1H, *J* = 7.2 Hz, pyrimidinyl H-5), 7.26 (s, 1H, =CH), 7.28 (t, 1H, *J* = 8.8 Hz, indole H-6), 7.30–7.37 (m, 3H, *p*-chlorophenyl H-2, H-6 and indole H-5), 7.45–7.68 (m, 3H, *p*-chlorophenyl H-3, H-5 and indole H-7), 7.81 (d, 1H, *J* = 8 Hz, indole H-4), 8.37–8.40 (m, 3H, indole H-2 and pyrimidinyl H-4, H-6); ^13^C NMR (100 MHz, DMSO-*d_6_*, *δ* = ppm) *δ* = 41.44, 64.58, 67.89, 111.06, 113,35, 115.97, 118.60, 123.77, 125.37, 125.58, 127.98, 128.70, 129.49, 130.71, 133.37, 134.38, 135.68, 136.19, 157.003, 161.62, 169.09; Anal.Calcd for C_26_H_24_ClN_5_O_3_S (522.02): C, 59.82; H, 4.63; N, 13.42. Found: C, 60.12; H, 4.80; N, 13.15.

#### (E)-1–(4-Benzylpipridin-1-yl)-2–(4-chlorophenyl)-3-[1-(methylsulfonyl)-1H-indol-3-yl]prop-2-en-1-one (4d)

White-off solid; 79%; mp 144–146 °C; IR (KBr) 3323 (NH), 3023 (CH-aromatic), 2923 (CH-aliphatic), 1633 (C=O), 1365, 1170 (SO_2_) cm^−1^; ^1^H NMR (400 MHz, DMSO-*d_6_*, *δ* = ppm) *δ* = 1.51–1.64 (m, 1H, CH(CH_2_)_2_), 1.73–1.84 (m, 4H, CH(CH_2_)_2_), 2.82–2.93 (m, 4H, N(CH_2_)_2_), 3.02 (s, 2H, CH_2_), 3.36 (s, 3H, SO_2_CH_3_), 6.87 (s, 1H, =CH), 7.15–7.28 (m, 3H, phenyl H-4 and indole H-5, H-6), 7.30–7.41 (m, 4H, phenyl H-2, H-6 and *p*-chlorophenyl H-2, H-6), 7.44–7.51 (m, 5H, indole H-7, phenyl H-3, H-5 and *p*-chlorophenyl H-3, H-5), 7.79 (d, 1H, *J* = 8 Hz, indole H-4), 7.81 (s, 1H, indole H-2); ^13^C NMR (100 MHz, DMSO-*d_6_*, *δ* = ppm) *δ* = 30.50, 37.95, 41.36, 42.41, 45.30, 113.35, 116.07, 119.72, 120.60, 123.75, 125.34, 125.90, 126.32, 128.65, 129.34, 129.46, 130.55, 132.69, 133.26, 134.43, 135.74, 138.30, 140.44, 168.61; EIMS (m/z) 533.11 (M + 1, 32.27%), 495.01 (100%). Anal.Calcd for C_30_H_29_ClN_2_O_3_S (532.08): C, 67.59; H, 5.48; N, 5.26. Found: C, 67.39; H, 5.57; N, 5.13.

#### General procedure for synthesis of compounds Ca–c

A mixture of the appropriate compound **Ba–c** (0.001 mol) and *N*,*N*,*N*',*N*'-tetramethyl-*O*-(1*H*-benzotriazol-1-yl)uranium hexafluorophosphate (HBTU) (0.001 mol, 0.37 g) in dimethylformamide (2 mL) was stirred for 30 min at room temperature. Then, *p*-aminoacetophenone derivative (**A**) (0.001 mol, 0.13 g) and a catalytic amount of triethylamine were added. The reaction mixture was stirred for 8 h at room temperature. Water (10 mL) was added. The product was extracted using ethylacetate. The combined extract was concentrated. The obtained crude compound was crystallised from 95% ethanol to give pure form of the desired compounds **Ca–c**.

#### N-(4-Acetylphenyl)-2-phenylacetamide (Ca)

Yellow solid; 82%; mp 118–120 °C; IR (KBr) 3163 (NH), 3042 (CH-aromatic), 2976 (CH-aliphatic), 1669, 1634 (2 C=O) cm^−1^; ^1^H NMR (400 MHz, DMSO-*d_6_*, *δ* = ppm) *δ* = 2.56 (s, 3H, CH_3_), 3.69 (s, 2H, CH_2_), 7.35–7.41 (m, 5H, phenyl H-2, H-3, H-4, H-5, H-6), 7.73 (d, 2H, *J* = 8 Hz, aminophenyl H-2, H-6), 7.93 (d, 2H, *J* = 8 Hz, aminophenyl H-3, H-5), 10.52 (s, 1H, NH, D_2_O exchangeable); ^13^C NMR (100 MHz, DMSO-*d_6_*, *δ* = ppm) *δ* = 26.90, 42.90, 118.70, 128.78, 129.71, 131.28, 131.93, 134.15, 135.43, 142.94, 169.47, 196.91; Anal.Calcd for C_16_H_15_NO_2_ (253.30): C, 75.87; H, 5.97; N, 5.53. Found: C, 75.64; H, 5.96; N, 5.37.

#### N-(4-Acetylphenyl)-2–(4-chlorophenyl)acetamide (Cb)

Yellow solid; 79%; mp 140–142 °C; IR (KBr) 3272 (NH), 3043 (CH-aromatic), 2928 (CH-aliphatic), 1669, 1628 (2 C=O) cm^−1^; ^1^H NMR (400 MHz, DMSO-*d_6_*, *δ* = ppm) *δ* = 2.51 (s, 3H, CH_3_), 3.69 (s, 2H, CH_2_), 7.35–7.41 (m, 4H, *p*-chlorophenyl H-2, H-3, H-5, H-6), 7.73 (d, 2H, *J* = 8.4 Hz, phenyl H-2, H-6), 7.93 (d, 2H, *J* = 8.4 Hz, phenyl H-3, H-5), 10.34 (s, 1H, NH, D_2_O exchangeable); ^13^C NMR (100 MHz, DMSO-*d_6_*, *δ* = ppm) *δ* = 26.88, 42.90, 118.86, 128.47, 128.72, 129.96, 131.59, 131.87, 132.24, 143.88, 167.98, 197.03; Anal.Calcd for C_16_H_14_ClNO_2_ (287.74): C, 66.79; H, 4.90; N, 4.87. Found: C, 66.88; H, 4.96; N, 4.71.

#### N-(4-Acetylphenyl)-2–(3,4,5-trimethoxyphenyl)acetamide (Cc)

Yellow solid; 81%; mp 110–112 °C; IR (KBr) 3307 (NH), 3051 (CH-aromatic), 2932 (CH-aliphatic), 1672, 1650 (2 C=O) cm^−1^; ^1^H NMR (400 MHz, DMSO-*d_6_*, *δ* = ppm) *δ* = 2.51 (s, 3H, CH_3_), 3.63 (s, 2H, CH_2_), 3.74 (s, 6H, 2OCH_3_), 3.77 (s, 3H, OCH_3_), 6.66 (s, 2H, trimethoxyphenyl H-2, H-6), 7.73 (d, 2H, *J* = 8.4 Hz, phenyl H-2, H-6), 7.93 (d, 2H, *J* = 8.4 Hz, phenyl H-3, H-5), 10.57 (s, 1H, NH, D_2_O exchangeable); ^13^C NMR (100 MHz, DMSO-*d_6_*, *δ* = ppm) *δ* = 26.88, 47.99, 56.32, 60.44, 107.06, 118.86, 129.95, 131.65, 132.18, 138.10, 143.98, 152.98, 168.29, 197.01; Anal.Calcd for C_19_H_21_NO_5_ (343.37): C, 66.46; H, 6.16; N, 4.08. Found: C, 66.32; H, 5.78; N, 3.84.

#### General procedure for the synthesis of compounds 5a–c

To a solution of the appropriate acetophenone derivative **Ca–c** (0.01 mol) in absolute ethanol (10 mL) containing sodium ethoxide (0.02 g Na metal, 0.01 mol, in 5 mL absolute ethanol), aldehyde derivative **2** (0.01 mol, 1.45 g) was added. The reaction mixture was stirred for 24 h at room temperature. The obtained solution was poured into ice-cold water and neutralised with few drops of Conc. HCl (indicated by litmus paper). The obtained solid was filtered off, dried, and crystallised from acetone to give pure form of the compounds **5a–c**.

#### (E)-N-{4-[3–(1-(methylsulfonyl)-1H-indol-3-yl)acryloyl]phenyl}-2-phenylacetamide(5a)

Yellow solid; 78%; mp 120–122 °C; IR (KBr) 3311 (NH), 3025 (CH-aromatic), 2924 (CH-aliphatic), 1672, 1628 (2 C=O), 1305, 1178 (SO_2_) cm^−1^; ^1^H NMR (400 MHz, DMSO-*d_6_*, *δ* = ppm) *δ* = 3.24 (s, 3H, SO_2_CH_3_), 3.67 (s, 2H, CH_2_), 7.22–7.27 (m, 7H, indole H-5, H-6 and phenyl H-2, H-3, H-4, H-5, H-6), 7.34 (d, 2H, *J* = 8.4 Hz, aminophenyl H-2, H-6), 7.50 (d, 1H, *J* = 7.6 Hz, indole H-7), 7.52 (d, 1H, *J* = 8 Hz, indole H-4), 7.53–7.73 (m, aminophenyl H-3, H-5 and COCH=CH), 8.00 (d, 1H, *J* = 11.2 Hz, COCH=CH), 8.10 (s, 1H, indole H-2), 9.91 (s, 1H, NH, D_2_O exchangeable);^13^C NMR (100 MHz, DMSO-*d_6_*, *δ* = ppm) *δ* = 41.55, 43.03, 112.05, 113.33, 119.98, 120.91, 122.78, 124.34, 127.85, 128.13, 129.71, 128.13, 129.71, 130.35, 134.42, 136.36, 137.01, 138.86, 144.86, 145.51, 168.53, 189.21; Anal.Calcd for C_26_H_22_N_2_O_4_S (458.53): C, 68.10; H, 4.84; N, 6.11. Found: C, 67.88; H, 4.96; N, 5.84.

#### (E)-2–(4-Chlorophenyl)-N-{4-[3–(1-(methylsulfonyl)-1H-indol-3-yl)acryloyl]phenyl}acetamide (5b)

White off solid; 73%; mp 112–114 °C; IR (KBr) 3325 (NH), 3023 (CH-aromatic), 2926 (CH-aliphatic), 1674, 1626 (2 C=O), 1259, 1176 (SO_2_) cm^−1^; ^1^H NMR (400 MHz, DMSO-*d_6_*, *δ* = ppm) *δ* = 3.61(s, 3H, SO_2_CH_3_), 3.71 (s, 2H, CH_2_), 7.22–7.24 (m, 3H, indole H-5, H-6, H-7), 7.34–7.41 (m, 7H, indole H-2, H-4, *p*-chlorophenyl H-2, H-3, H-5, H-6 and COCH=CH), 7.73 (d, 2H, *J* = 8.8 Hz, phenyl H-2, H-6), 7.93 (d, 2H, *J* = 8.8 Hz, phenyl H-3, H-5), 8.39 (d, 1H, *J* = 11.2 Hz, COCH=CH), 9.31 (s, 1H, NH, D_2_O exchangeable); ^13^C NMR (100 MHz, DMSO-*d_6_*, *δ* = ppm) *δ* = 42.89, 48.00, 109.27, 113.33, 118.41, 122.48, 124.05, 125.35, 127.30, 128.50, 129.72, 130.35, 131.64, 132.29, 132.85, 133.50, 135.07, 135.43, 143.30, 143.95, 168.90, 186.70; Anal.Calcd for C_26_H_21_ClN_2_O_4_S (492.97): C, 63.35; H, 4.29; N, 5.68. Found: C, 63.10; H, 4.15; N, 5.50.

#### (E)-N-{4-[3–(1-(methylsulfonyl)-1H-indol-3-yl)acryloyl]phenyl}-2–(3,4,5-trimethoxyphenyl)acetamide (5c)

White off solid; 75%; mp 176–178 °C; IR (KBr) 3325 (NH), 3019 (CH-aromatic), 2926 (CH-aliphatic), 1677, 1629 (2 C=O), 1333, 1125 (SO_2_) cm^−1^; ^1^H NMR (400 MHz, DMSO-*d_6_*, *δ* = ppm) *δ* = 3.43 (s, 3H, SO_2_CH_3_), 3.69 (s, 3H, OCH_3_), 3.77 (s, 6H, 2OCH_3_), 3.83 (s, 2H, CH_2_), 6.52 (s, 2H, trimethoxyphenyl H-2, H-6), 7.44–7.48 (m, 2H, indole H-5, H-6), 7.49–7.52 (m, 3H, phenyl H-2, H-6 and CH=CHCO), 7.53 (d, 1H, *J* = 7.2 Hz, indole H-7), 7.93 (d, 2H, *J* = 8 Hz, phenyl H-3, H-5), 8.21 (d, 1H, *J* = 7.6 Hz, indole H-4), 8.23–8.29 (m, 2H, indole H-2 and CH=CHCO), 10.47 (s, 1H, NH, D_2_O exchangeable); ^13^C NMR (100 MHz, DMSO-*d_6_*, *δ* = ppm) *δ* = 41.33, 44.07, 56.28, 60.43, 107.18, 112.89, 113.65, 118.88, 121.11, 123.96, 125.35, 126.01, 126.52, 127.30, 129.94, 131.40, 135.20, 135.85, 138.11, 144.32, 145.1, 152.98, 168.90, 187.41; EIMS (m/z) 549.46 (M + 1, 20.75%), 55.66 (100%). Anal.Calcd for C_29_H_28_N_2_O_7_S (548.16): C, 63.49; H, 5.14; N, 5.11. Found: C, 63.37; H, 5.41; N, 4.84.

#### General procedure for the synthesis of compounds Fa,b

A mixture of the appropriate *N*-acetyl derivative **Da,b** (0.001 mol), benzylpipridine (**E**) (0.012 mol, 2.10 g), K_2_CO_3_ (0.001 mol, 0.13 g), and catalytic amount of KI in acetone (15 mL), was heated under reflux temperature for 6–8 h (monitored by TLC). The obtained solution was cooled and poured into water. The solid obtained was filtered, dried, and crystallised from 95% ethanol to give pure form of compounds **Fa,b**.

#### N-(3-Acetylphenyl)-2–(4-benzylpiperidin-1-yl)acetamide (Fa)

White solid; 79%; mp 126–128 °C; IR (KBr) 3206 (NH), 3053 (CH-aromatic), 2926 (CH-aliphatic), 1665, 1616 (2 C=O) cm^−1^; ^1^H NMR (400 MHz, DMSO-*d_6_*, *δ* = ppm) *δ* = 1.3–1.34 (m, 2H, CHCH_2_), 1.53–1.56 (m, 3H, CHCH_2_), 2.05–2.10 (m, 2H, CH_2_), 2.50 (s, 2H, CH_2_), 2.51 (s, 3H, CH_3_), 2.83–2.88 (m, 2H, CH_2_), 3.09 (s, 2H, CH_2_), 7.16–7.19 (m, 4H, phenyl H-2, H-3, H-5, H-6), 7.27 (t, 1H, *J* = 8.4 Hz, phenyl H-4), 7.44 (t, 2H, *J* = 8 Hz, aminophenyl H-5), 7.47 (d, 1H, *J* = 7.8 Hz, aminophenyl H-4), 7.67 (d, 1H, *J* = 8 Hz, aminophenyl H-6), 8.22 (s, 1H, aminophenyl H-2), 9.86 (s, 1H, NH, D_2_O exchangeable); ^13^C NMR (100 MHz, DMSO-*d_6_*, *δ* = ppm) *δ* = 26.95, 31.96, 37.33, 42.66, 53.77, 62.56, 119.24, 123.87, 124.45, 126.21, 128.53, 129.44, 129.55, 137.57, 139.51, 140.79, 169.12, 198.15; Anal.Calcd for C_22_H_26_N_2_O_2_ (350.20): C, 75.40; H, 7.48; N, 7.99. Found: C, 75.28; H, 7.55; N, 8.01.

#### N-(4-Acetylphenyl)-2–(4-benzylpiperidin-1-yl)acetamide (Fb)

White solid; 86%; mp 94–96 °C; IR (KBr) 3210 (NH), 3054 (CH-aromatic), 2925 (CH-aliphatic), 1667, 1617 (2 C=O) cm^−1^; ^1^H NMR (400 MHz, DMSO-*d_6_*, *δ* = ppm) *δ* = 1.23–1.27 (m, 2H, CHCH_2_), 1.32–1.46 (m, 3H, CHCH*_2_*), 2.04–2.12 (m, 2H, CH_2_), 2.51 (s, 2H, CH_2_), 2.53 (s, 3H, CH_3_), 2.78–2.85 (m, 2H, CH_2_), 3.11 (s, 2H, CH_2_), 7.15 − 7.18 (m, 3H, phenyl H-2, H-4, H-6), 7.19 (t, 2H, *J* = 7.8 Hz, phenyl H-3, H-5), 7.78 (d, 2H, *J* = 7.6 Hz, aminophenyl H-2, H-6), 7.91 (d, 2H, *J* = 7.6 Hz, aminophenyl H-3, H-5), 9.98 (s, 1H, NH, D_2_O exchangeable); ^13^C NMR (100 MHz, DMSO-*d_6_*, *δ* = ppm) *δ* = 26.90, 32.05, 37.39, 42.82, 53.83, 62.72, 119.10, 126.22, 128.61, 129.44, 129.86, 132.30, 140.81, 143.39, 169.76, 196.93; Anal.Calcd for C_22_H_26_N_2_O_2_ (350.20): C, 75.40; H, 7.48; N, 7.99. Found: C, 73.37; H, 7.41; N, 7.84.

#### General procedure for the synthesis of compounds 6a,b

To a solution of the appropriate acetophenone derivative **Fa,b** (0.01 mol) in methanol (10 mL) containing KOH (0.01 mol, 0.56 g), aldehyde derivative **2** (0.01 mol, 1.45 g) was added. The reaction mixture was stirred for 24 h at room temperature. The obtained solution evaporated into dryness. The obtained crude solid was crystallised from 95% ethanol to give pure form of the compounds **6a,b**.

#### (E)-2–(4-Benzylpiperidin-1-yl)-N-{3-[3–(1-(methylsulfonyl)-1H-indol-3-yl)acryloyl]phenyl}acetamide (6a)

White off solid; 82%; mp 120–122 °C; IR (KBr) 3221 (NH), 3022 (CH-aromatic), 2915 (CH-aliphatic), 1664, 1642 (2 C=O), 1360, 1166 (SO_2_) cm^−1^; ^1^H NMR (400 MHz, DMSO-*d_6_*, *δ* = ppm) *δ* = 1.31–1.57 (m, 4H, CH(CH_2_)_2_), 1.99 (m, 1H, CH(CH_2_)_2_), 2.73 (s, 2H, CH_2_), 2.71 (s, 2H, COCH_2_), 2.76–2.85 (m, 4H, N(CH_2_)_2_), 3.10 (s, 3H, SO_2_CH_3_), 7.18–7.28 (m, 7H, benzyl H-2, H-3, H-4, H-5, H-6 and indole H-5, H-6), 7.47–7.69 (m, 4H, phenyl H-4, H-5, H-6 and CH=CH-CO), 7.91–7.94 (m, 4H, phenyl H-2, indole H-4, H-7 and CH=CH-CO), 8.23 (s, 1H, indole H-2), 9.88 (s, 1H, NH, D_2_O exchangeable); ^13^C NMR (100 MHz, DMSO-*d_6_*, *δ* = ppm) *δ* = 26.57, 30.74, 37.03, 43.03, 54.32, 63.20, 112.70, 119.25, 121.55, 123.85, 124.49, 125.93, 126.11, 126.92, 128.58, 129.42, 129.54, 131.93, 132.58, 133.86, 135.80, 137.82, 138.11, 139.58, 140.94, 145.10, 169.11, 198.14; EIMS (m/z) 556.38 (M^+.^, 12.74%), 93.36 (100%). Anal.Calcd for C_33_H_33_N_3_O_4_S (555.69): C, 69.17; H, 5.99; N, 7.56. Found: C, 69.22; H, 6.05; N, 7.32.

#### (E)-2–(4-Benzylpiperidin-1-yl)-N-{4-[3–(1-(methylsulfonyl)-1H-indol-3-yl)acryloyl]phenyl}acetamide (6b)

White off solid; 82%; mp 211–213 °C; IR (KBr) 3219 (NH), 3054 (CH-aromatic), 2923 (CH-aliphatic), 1664, 1635 (2 C=O), 1398, 1164 (SO_2_) cm^−1^; ^1^H NMR (400 MHz, DMSO-*d_6_*, *δ*= ppm) *δ* = 1.29–1.57 (m, 4H, CH(CH_2_)_2_), 2.06–2.11 (m, 1H, CH(CH_2_)_2_), 2.53 (s, 2H, CH_2_), 2.73–2.86 (m, 4H, N(CH_2_)_2_), 2.89 (s, 2H, COCH_2_), 3.13 (s, 3H, SO_2_CH_3_), 7.16–7.19 (m, 3H, benzyl H-4 and indole H-5, H-6), 7.28 (t, 2H, *J*= 8.4 Hz, benzyl H-3, H-5), 7.52–7.54 (d, 2H, *J*= 8.4 Hz, benzyl H-2, H-6), 7.71 (d, 1H, *J*= 15.6 Hz, COCH=CH), 7.83 (d, 2H, *J* = 8.8 Hz, phenyl H-3, H-5), 7.95–8.00 (m, 4H, indole H-7, phenyl H-2, H-6 and COCH=CH), 8.15 (s, 1H, indole H-2), 8.18 (d, 1H, *J* = 8.8 Hz, indole H-4), 10.18 (s, 1H, NH, D_2_O exchangeable); ^13^C NMR (100 MHz, DMSO-*d_6_*, *δ* = ppm) *δ* = 31.02, 35.45, 37.31, 42.39, 53.41, 63.49, 110.61, 114.53, 118.700, 122.49, 124.91, 125.89, 126.28, 127.30, 128.14, 129.06, 130.07, 131.27, 132.29, 134.42, 134.78, 140.44, 142.01, 143.58, 169.47, 188.06; Anal.Calcd for C_32_H_33_N_3_O_4_S (555.69): C, 69.17; H, 5.99; N, 7.56. Found: C, 68.80; H, 6.05; N, 7.33.

#### General procedure for the synthesis of compounds 7a–c

A mixture of indole carboxaldehyde derivative **2** (0.01 mol, 1.45 g) and the appropriate hydrazide derivative (0.01 mol) was dissolved in glacial acetic acid (15 mL). The reaction mixture was heated under reflux temperature for 3–5 h (monitored by TLC). The solution was concentrated to half its volume. The obtained solid was filtered, dried, and crystallised from 95% ethanol to give pure form of the compounds **7a–c**.

#### (E)-N'-{[1-(Methylsulfonyl)-1H-indol-3-yl]methylene}benzohydrazide (7a)

White off solid; 83%; mp 114–116 °C; IR (KBr) 3222 (NH), 3069 (CH-aromatic), 2924 (CH-aliphatic), 1652 (C=O), 1355, 1162 (SO_2_) cm^−1^; ^1^H NMR (400 MHz, DMSO-*d_6_*, *δ* = ppm) *δ* = 3.38 (s, 3H, SO_2_CH_3_), 7.45–7.50 (m, 2H, indole H-5, H-6), 7.55 (t, 2H, *J* = 7.2 Hz, phenyl H-3, H-5), 7.59 (t, 1H, *J* = 8.4 Hz, phenyl H-4), 7.90 (d, 1H, *J* = 8 Hz, indole H-7), 7.95 (d, 2H, *J* = 7.2 Hz, phenyl H-2, H-6), 8.13 (s, 1H, indole H-2), 8.52 (d, 1H, *J* = 7.2 Hz, indole H-4), 8.65 (s, 1H, =CH), 11.93 (s, 1H, NH, D_2_O exchangeable); ^13^C NMR (100 MHz, DMSO-*d_6_*, *δ* = ppm) *δ* = 41.66, 113.38, 117.43, 119.80, 123.78, 124.42, 126.09, 127.17, 128.09, 130.38, 132.19, 133.98, 135.48, 142.95, 163.53; Anal.Calcd for C_17_H_15_N_3_O_3_S (341.38): C, 59.81; H, 4.43; N, 12.31. Found: C, 60.08; H, 4.15; N, 12.13.

#### (E)-N'-{[1-(Methylsulfonyl)-1H-indol-3-yl]methylene}-2-phenylacetohydrazide (7b)

White off solid; 81%; mp 194–196 °C; IR (KBr) 3232 (NH), 3028 (CH-aromatic), 2925 (CH-aliphatic), 1675 (C=O), 1364, 1168 (SO_2_) cm^−1^; ^1^H NMR (400 MHz, DMSO-*d_6_*, *δ* = ppm) *δ* = 3.36 (s, 3H, SO_2_CH_3_), 3.37 (s, 2H, CH_2_), 7.26–7.29 (m, 2H, indole H-5, H-6), 7.37 (t, 1H, *J* = 8 Hz, phenyl H-4), 7.41–7.50 (m, 5H, phenyl H-2, H-3, H-5, H-6 and indole H-7), 7.75 (d, 1H, *J* = 8 Hz, indole H-4), 7.80 (s, 1H, indole H-2), 7.99 (s, 1H, =CH), 12.77 (s, 1H, NH, D_2_O exchangeable); ^13^C NMR (100 MHz, DMSO-*d_6_*, *δ* = ppm) *δ* = 41.52, 42.50, 113.37, 115.69, 119.98, 124.15, 125.76, 126.94, 128.42, 129.17, 129.45, 129.68, 133.65, 134.03, 137.42, 168.38; EIMS (*m/z*) 355.86 (M + 1, 5.63%), 355.21 (M^+.^, 17.15%), 222.11 (100%). Anal.Calcd for C_18_H_17_N_3_O_3_S (355.41): C, 60.83; H, 4.82; N, 11.82. Found: C, 60.79; H, 4.57; N, 12.10.

#### (E)-2–(4-Chlorophenyl)-N'-{[1-(methylsulfonyl)-1H-indol-3-yl]methylene}acetohydrazide (7c)

Yellow solid; 78%; mp 251–253 °C; IR (KBr) 3232 (NH), 3084 (CH-aromatic), 2933 (CH-aliphatic), 1669 (C=O), 1365, 1168 (SO_2_) cm^−1^; ^1^H NMR (400 MHz, DMSO-*d_6_*, *δ* = ppm) *δ* = 3.52 (s, 3H, SO_2_CH_3_), 4.08 (s, 2H, CH_2_), 7.34–7.47 (m, 4H, *p*-chlorophenyl H-3, H-5 and indole H-5, H-6), 7.88 (d, 1H, *J* = 8 Hz, indole H-7), 8.07 (s, 1H, indole H-2), 8.13 (d, 2H, *J* = 8 Hz, *p*-chlorophenyl H-2, H-6), 8.27–8.40 (m, 2H, indole H-4 and = CH), 11.47 (s, 1H, NH, D_2_O exchangeable); ^13^C NMR (100 MHz, DMSO-*d_6_*, *δ* = ppm) *δ* = 38.74, 41.61, 113.33, 117.12, 123.11, 123.61, 124.30, 126.03, 127.07, 128.60, 128.72, 131.44, 131.58, 135.39, 141.72, 172.60; Anal.Calcd for C_18_H_16_ClN_3_O_3_S (389.86): C, 55.45; H, 4.14; N, 10.78. Found: C, 55.63; H, 4.07; N, 10.97.

#### General procedure for the synthesis of (E)-2-cyano-N'-{[1-(Methylsulfonyl)-1H-indol-3-yl]methylene}acetohydrazide (8)

A mixture of indole carboxaldehyde derivative **2** (0.01 mol, 1.45 g) and cyanoacetic acid hydrazide (0.01 mol, 0.99 g) was dissolved in absolute ethanol (15 mL). The reaction mixture was heated under reflux temperature for 3 h. The obtained solid was filtered on hot, washed with ethanol, and crystallised from 95% ethanol to give pure form of the compound **8**. White off solid; 89%; mp 217–219 °C; IR (KBr) 3175 (NH), 3083 (CH-aromatic), 2960 (CH-aliphatic), 2260 (C≡N), 1681 (C=O), 1365, 1171 (SO_2_) cm^−1^; ^1^H NMR (400 MHz, DMSO-*d_6_*, *δ* = ppm) *δ* = 3.38 (s, 3H, SO_2_CH_3_), 4.30 (s, 2H, CH_2_), 7.42–7.51 (m, 2H, indole H-5, H-6), 7.89 (d, 1H, *J* = 8 Hz, indole H-7), 8.14 (s, 1H, indole H-2), 8.24 (s, 1H, =CH), 8.28 (d, 1H, *J* = 8 Hz, indole H-4), 11.81 (s, 1H, NH, D_2_O exchangeable); ^13^C NMR (100 MHz, DMSO-*d_6_*, *δ* = ppm) *δ* = 25.18, 41.71, 113.38, 116.70, 119.40, 123.51, 126.75, 131.05, 135.39, 140.28, 143.07, 159.17, 164.99; Anal.Calcd for C_13_H_12_N_4_O_3_S (304.32): C, 51.31; H, 3.97; N, 18.41. Found: C, 51.02; H, 4.15; N, 18.37.

#### General procedure for the synthesis of compounds 9a–c

A mixture of compound **8** (0.001 mol, 0.30 g) and the appropriate arylidene derivative (0.001 mol) was dissolved in absolute ethanol (15 mL). The reaction mixture was heated under reflux temperature for 4–6 h (monitored by TLC). The solution was concentrated and the solid obtained was filtered, dried, and crystallised from 95% ethanol to give pure form of the compounds **9a–c**.

#### (E)-6-Amino-4-(4-methoxyphenyl)-1-{[(1-(methylsulfonyl)-1H-indol-3-yl) methylene]amino}-2-oxo-1,2-dihydropyridine-3,5-dicarbonitrile (9a)

Yellow solid; 79%; mp 210–212 °C; IR (KBr) 3338, 3132 (NH_2_), 3028 (CH-aromatic), 2927 (CH-aliphatic), 2209 (2 C≡N), 1675 (C=O), 1362, 1168 (SO_2_) cm^−1^; ^1^H NMR (400 MHz, DMSO-*d_6_*, *δ* = ppm) *δ* = 3.52 (s, 3H, SO_2_CH_3_), 3.84 (s, 3H, OCH_3_), 7.08–7.39 (m, 3H, methoxyphenyl H-3, H-5 and indole H-6), 7.41–7.62 (m, 6H, methoxyphenyl H-2, H-6, indole H-5, H-7 and NH_2_, D_2_O exchangeable), 7.98 (s, 1H, indole H-2), 8.10–8.22 (m, 2H, indole H-4 and = CH); ^13^C NMR (100 MHz, DMSO-*d_6_*, *δ* = ppm) *δ* = 41.09, 55.34, 77.41, 112.93, 114.13, 114.64, 114.74, 114.88, 118.92, 121.33, 124.12, 125.75, 128.53, 128.60, 130.82, 134.65, 143.66, 154.03, 157.17, 160.96, 165.67, 171.68; Anal.Calcd for C_24_H_18_N_6_O_4_S (486.50): C, 59.25; H, 3.73; N, 17.27. Found: C, 60.06; H, 3.45; N, 17.13.

#### (E)-6-Amino-4–(3,4-dimethoxyphenyl)-1-{[(1-(methylsulfonyl)-1H-indol-3-yl)methylene]amino}-2-oxo-1,2-dihydropyridine-3,5-dicarbonitrile (9b)

Yellow solid; 73%; mp 211–213 °C; IR (KBr) 3332, 3137 (NH_2_), 3063 (CH-aromatic), 2925 (CH-aliphatic), 2359 (2 C≡N), 1677 (C=O), 1369, 1175 (SO_2_) cm^−1^; ^1^H NMR (400 MHz, DMSO-d_6_, δ = ppm) *δ* = 3.51 (s, 3H, SO_2_CH_3_), 3.85 (s, 3H, OCH_3_), 3.88 (s, 3H, OCH_3_), 7.08 (s, 1H, dimethoxyphenyl H-2), 7.11–7.19 (m, 3H, dimethoxyphenyl H-5, H-6, and indole H-6), 7.42 (t, 1H, J = 6.8 Hz, indole H-5), 7.50–7.78 (m, 4H, indole H-2, H-7 and NH_2_, D_2_O exchangeable), 7.96–7.99 (m, 2H, indole H-4, and = CH); ^13^C NMR (100 MHz, DMSO-d_6_, *δ* = ppm) *δ* = 41.47, 56.54, 60.05, 78.92, 104.18, 107.32, 113.34, 114.51, 115.32, 115.87, 115.90, 116.48, 119.34, 120.55, 121.85, 124.07, 126.93, 131.27, 134.42, 139.22, 152.45, 153.10, 157.17, 163.47, 167.53; Anal.Calcd for C_25_H_20_N_6_O_5_S (516.53): C, 58.13; H, 3.90; N, 16.27. Found: C, 58.09; H, 3.85; N, 16.18.

#### (E)-6-Amino-1-{[(1-(methylsulfonyl)-1H-indol-3-yl)methylene]amino}-2-oxo-4–(3,4,5-trimethoxyphenyl)-1,2-dihydropyridine-3,5-dicarbonitrile (9c)

Yellow solid; 75%; mp 210–212 °C; IR (KBr) 3329, 3130 (NH_2_), 3025 (CH-aromatic), 2930 (CH-aliphatic), 2213 (2 C≡N), 1669 (C=O), 1365, 1170 (SO_2_) cm^−1^; ^1^H NMR (400 MHz, DMSO-*d_6_*, *δ* = ppm) *δ* = 3.32 (s, 3H, SO_2_CH_3_), 3.62 (s, 3H, OCH_3_), 3.85 (s, 6H, 2OCH_3_), 6.89 (s, 2H, trimethoxyphenyl H-2, H-6), 6.91 (s, 2H, NH_2_, D_2_O exchangeable), 7.47–7.53 (m, 4H, indole H-2, H-5, H-6, H-7), 7.96 (d, 1H, *J* = 6.8 Hz, indole H-4), 8.22 (s, 1H, =CH); ^13^C NMR (100 MHz, DMSO-*d_6_*, *δ* = ppm) *δ* = 41.77, 56.28, 78.92, 105.11, 106.40, 107.32, 116.77, 118.69, 119.64, 121.20, 124.34, 125.34, 126.30, 126.93, 128.48, 131.27, 134.14, 138.40, 143.30, 153.67, 156.52, 159.50, 163.18, 169.40; EIMS (m/z) 547.25 (M^+.^, 8.99%), 357.52 (100%). Anal.Calcd for C_26_H_22_N_6_O_6_S (546.55): C, 57.14; H, 4.06; N, 15.38. Found: C, 57.49; H, 4.15; N, 15.19.

#### General procedure for the synthesis of compounds 10a,b

A mixture of compound **8** (0.001 mol, 0.30 g), the appropriate isothiocyanate derivative (0.001 mol) and sulphur (0.001 mol, 0.03 g) were dissolved in absolute ethanol (15 mL). A catalytic amount of triethylamine was added to the mixture. The reaction mixture was heated under reflux temperature for 10–12 h (monitored by TLC). The obtained solid was filtered while hot, dried, and crystallised from 95% ethanol to give pure form of the compounds **10a,b**.

#### (E)-4-Amino-3-ethyl-N'-{[1-(methylsulfonyl)-1H-indol-3-yl]methylene}-2-thioxo-2,3-dihydrothiazole-5-carbohydrazide (10a)

Yellow solid; 72%; mp 207–209 °C; IR (KBr) 3349, 3252 (NH_2_ and NH), 3028 (CH-aromatic), 2929 (CH-aliphatic), 1624 (C=O), 1361, 1167 (SO_2_), 1128 (C=S) cm^−1^; ^1^H NMR (400 MHz, DMSO-d_6_, δ = ppm) *δ* = 1.21 (t, 3H, J = 8 Hz, CH_2_CH_3_), 3.55 (s, 3H, SO_2_CH_3_), 4.27 (q, 2H, J = 8 Hz, CH_2_CH_3_), 7.41–7.51 (m, 2H, indole H-5, H-6), 7.91–8.08 (m, 4H, indole H-4, H-7 and NH_2_, D_2_O exchangeable), 8.26 (s, 1H, indole H-2), 8.38 (s, 1H, =CH), 11.33 (s, 1H, NH, D_2_O exchangeable); ^13^C NMR (100 MHz, DMSO-d_6_, *δ* = ppm) *δ* = 12.07, 40.67, 41.46, 76.43, 108.61, 113.62, 118.70, 121.20, 124.22, 126.08, 130.10, 131.65, 142.01, 161.24, 168.54, 182.70; EIMS (m/z) 424.63 (M + 1, 48.52%), 72.79 (100%). Anal.Calcd for C_16_H_17_N_5_O_3_S_3_ (423.53): C, 45.37; H, 4.05; N, 16.54. Found: C, 45.67; H, 4.15; N, 16.78.

#### (E)-4-Amino-N'-{[1-(methylsulfonyl)-1H-indol-3-yl]methylene}-3-phenyl-2-thioxo-2,3-dihydrothiazole-5-carbohydrazide (10b)

Yellow solid; 75%; mp 187–189 °C; IR (KBr) 3384, 3268 (NH_2_ and NH), 3028 (CH-aromatic), 2925 (CH-aliphatic), 1631 (C=O), 1362, 1165 (SO_2_), 1249 (C=S) cm^−1^; ^1^H NMR (400 MHz, DMSO-d_6_, δ = ppm) *δ* = 3.64 (s, 3H, SO_2_CH_3_), 7.21–7.53 (m, 6H, indole H-5, H-6, phenyl H-2, H-6, and NH_2_, D_2_O exchangeable), 7.66–7.98 (m, 3H, phenyl H-3, H-4, H-5), 8.08 (s, 1H, indole H-2), 8.27 (d, 1H, *J* = 7.8 Hz, indole H-7), 8.37 (d, 1H, *J* = 8 Hz, indole H-4), 9.06 (s, 1H, =CH), 11.83 (s, 1H, NH, D_2_O exchangeable); ^13^C NMR (100 MHz, DMSO-d_6_, δ = ppm) *δ* = 41.76, 92.52, 107.99, 112.05, 121.56, 122.49, 123.71, 124.06, 125.01, 126.28, 128.78, 129.40, 130.35, 138.22, 143.29, 157.45, 165.04, 197.51; Anal.Calcd for C_20_H_17_N_5_O_3_S_3_ (471.58): C, 50.94; H, 3.63; N, 14.85. Found: C, 51.18; H, 3.55; N, 14.78.

### Biological evaluation

#### Assessment of AChE and BuChE inhibitory activities

The inhibitory efficacy of synthesised compounds **3a–c**, **4a–d**, **5a–c**, **6a,b**, **7a–c**, **9a–c,** and **10a,b** against AChE and BuChE in comparison with reference drugs tacrine and donepezil, was investigated using a modified Elman’s test. The reaction of thiocholine with 5,5-dithio-bis (2-nitrobenzoic) acid (DTNB), generates a yellow chromophore that can be quantified at 412 nm^41^.

#### Inhibition of Aβ1–42 self-induced aggregation

Inhibition of self-induced β-amyloid Aβ1–42 assessment was performed for the selected compounds **3c**, **4a**, **4b**, **4d**, **5b**, **6b**, **7c,** and **10b** in comparison with tacrine. Screening Aβ42 ligands that could prevent aggregation is critical for developing potential therapeutic treatments. In BioVision’s Beta-Amyloid 1–42 (Aβ42) Ligand Screening kit, a dye binds to the beta-sheets of an aggregated amyloid peptide resulting in an intense fluorescent product at wave length 450 nm using a BIOLINE ELISA reader. In the presence of an Aβ42 ligand, this reaction is impeded/abolished resulting in a decrease or total loss of fluorescence. This assay is useful in screening Aβ42 ligands for developing potential therapeutic agents against AD^42^. The assessment was performed according to BioVision’s Beta-Amyloid 1–42 (Aβ42) Ligand Screening Kit Catalog No. K570-100.

#### Assessments of anti-neuroinflammatory activity

The most active tested compounds, namely, **5b** and **6b** were selected to be assessed on NO, IL-1B, TNF-α, and COX-2 production in LPS-induced BV2 microglial cell lines. LPS was the used positive control.

NO plays an important role in neurotransmission, vascular regulation, immune response, and apoptosis. NO is rapidly oxidised to nitrite and nitrate which are used to quantitate NO. NO was estimated using the Abcam ELISA kit (catalog No. ab65328). Briefly, enzyme was added to cell lysate, followed with cofactor and incubated at room temperature for 60 min, after that Griess reagent was added and incubated at room temperature for 10 min., finally optical density was measured at 540 nm.

*For COX-2*, all reagents, samples, and standards were prepared as Kit instructions, then 100 μl of standard or sample were added to each well, incubated 2.5 h at room temperature. Then 100 μl of prepared biotin antibody was added to each well, incubated an hour at room temperature. After that 100 μl of prepared Streptavidin solution was added. Then it was incubated for 45 min at room temperature. An aliquot of 100 μl TMB One-Step Substrate Reagent was added to each well, incubated 30 min at room temperature, 50 μL stop solution added to each well. Finally, the optical density was read at 450 nm immediately.

*For IL-1β*, this assay employs the quantitative sandwich enzyme immunoassay technique. A monoclonal antibody specific for human IL-1β has been pre-coated onto a micro plate. Standards and samples are piped into the wells and any IL-1β present is bound by the immobilised antibody. After washing away any unbound substances, an enzyme-linked polyclonal antibody specific for human IL-1β is added to the wells. Following a wash to remove any unbound antibody-enzyme reagent, a substrate solution is added to the wells and colour develops in proportion to the amount of IL-1β bound in the initial step. The colour development is stopped and the intensity of the colour is measured^43^, the method of assessment was performed as instructed in the IL-1 β R&D system ELISA kit (catalog No. DLB50).

*For TNF-α*, cell lysate was used to assess TNF-α using the MyBioSource ELISA kit (Catalog No: MBS2502004). This assay employs the quantitative sandwich enzyme immunoassay technique^44^. Briefly, 100 µl of the samples were added to each well. Incubate for 90 min at 37 °C, and immediately add 100 μl of Biotinylated Detection Ab working solution to each well. Incubate for 1 h at 37 °C, then add 350 µl of wash buffer to each well, 100 μl of HRP Conjugate working solution to each well was added, incubated for 30 min at 37 °C. Add 90 μl of Substrate Reagent to each well. Incubate for about 15 min at 37 °C. Then, 50 μl of Stop Solution was added to each well. Finally, the optical density of each well was detected at 450 nm.

#### Cytotoxicity on SH-SY5Y and THLE2 cell lines

##### Cell culture protocol

Microglia cell Line, BV-2, human neuroblastoma (SH-SY5Y), and normal hepatic (THLE2) cells were obtained from American Type Culture Collection, cells were cultured using DMEM (Invitrogen/Life Technologies, Carlsbad, CA) supplemented with 10% FBS (Hyclone), 10 µg/mL of insulin (Sigma, St. Louis, MO), and 1% penicillin-streptomycin. All of the other chemicals and reagents were from Sigma, or Invitrogen. Plate cells (cells density 1.2 − 1.8 × 10,000 cells/well) in a volume of 100 µL complete growth medium + 100 µL of the tested compounds per well in a 96-well plate for 24 h before the MTT assay.

After treatment of cells with the serial concentrations of the compound to be tested, incubation is carried out for 48 h at 37 °C, and then the plates are to be examined under the inverted microscope and proceed for the MTT assay^45^.

##### *In vitro* cell viability assay (MTT assay method)

The MTT method is simple, accurate, and yields reproducible results. It is used to investigate cytotoxicity of **5b** and **6b** in human neuroblastoma (SH-SY5Y) and normal hepatic (THLE2) cell lines. Cells were seeded in wells at number 10^6^ cells/cm^2^. Each test should include a blank containing complete medium without cells.

Solutions of MTT, dissolved in medium or balanced salt solutions without red phenol, are yellowish in colour. Add reconstituted MTT to an amount equal to 10% of the cultural medium volume. Then the cultures were returned to the incubator for 2–4 h.

Mitochondrial dehydrogenases of viable cells cleave the tetrazolium ring, yielding purple formazan crystals which are insoluble in aqueous solutions. The crystals are dissolved in acidified isopropanol. The resulting purple solution is spectrophotometrically measured at a wavelength of 570 nm. Measure the background absorbance of multiwell plates at 690 nm and subtract from the 570 nm measurement.

An increase or decrease in cell number results in a concomitant change in the amount of formazan formed, indicating the degree of cytotoxicity caused by the test material.

### Docking study

To identify molecular features that might be responsible for the biological activity of synthesised compounds and to predict their mechanism of action, a docking study was performed. X-ray crystal structure of *rh*AChE in complex with donepezil (https://www.rcsb.org/structure/4EY, PDB ID: 4EY7) and *h*BuChE with its ligand (https://www.rcsb.org/structure/4tpk, PDB ID: 4TPK) were downloaded from Protein Data Bank at Research Collaboration for Structural Bioinformatics (RSCB) Protein Database (PDB). All molecular modelling calculations and docking studies were carried out using Molecular Operating Environment Software (MOE 2014.0901). All water molecules were deleted. To ensure the accuracy of the docking protocol, validation was performed by re-docking the co-crystallised ligand (donepezil) into the *rh*AChE active site and *N*-{[1–(2,3-dihydro-1*H*-inden-2-yl)piperidin-3-yl]methyl}-N-(2-methoxyethyl)-2-naphthamide into *h*BuChE active site with a resolution of 2.35 and 2.7 Å, and energy score of −17.2793 and −16.4403 Kcal/mol, respectively**.**

Selected target compounds were protonated, energy minimised by Merk Molecular Force Field (MMFF94X), and docked into enzyme active sites using the same protocol for ligand compounds. The most stable conformer was chosen and amino acid interactions were depicted. All docking data are summarised in Table 2.

### ADMET study

#### Predicted molecular properties and drug-likeness

To evaluate drug-likeness of the most active synthesised target derivatives **3c**, **4a**, **4d**, **5b,** and **6b**, molinspiration (2018.02 version)^46^ was used to calculate molecular properties such as MW, number of the hydrogen-bond acceptor (nON), number of hydrogen-bond donors (nOHNH), partition coefficient (log*p*), number of rotatable bonds (nrotb), topological polar surface area (TPSA), absorption percentage (%Abs), which was calculated using formula %Abs = 109 – (0.345 × TPSA), and violation of Lipinski’s rule of five (n violation). Both acceptable values and predicted results of target compounds and standard drugs are listed in Table 3.

#### In silico ADME prediction

PreADME online server^47^ was used to predict *in silico* ADME properties of the selected compounds **3c**, **4a**, **4d**, **5b,** and **6b** and compared with donepezil and tacrine drugs. Human intestinal absorption (HIA), cell permeability of CaCo-2 cell and Maden Darby Canine Kideny (MDCK) cell, PPB, BBB (BBB), SP, and pure water solubility were calculated and predicted values are listed in Table 4.

#### Predicted toxicity properties

PreADMET online server^47^ was used to predict toxicity properties using the AMES test, rodent carcinogenicity assay (mice and rats), and hERG-inhibition. The obtained results for compounds **3c**, **4a**, **4d**, **5b**, **6b**, donepezil, and tacrine are recorded in Table 5.

#### Metabolism prediction

Metabolism prediction for the tested compounds **3c**, **4a**, **4d**, **5b,** and **6b** was examined using Swissadme online server^48^. The most important parameters used to measure metabolism and excretion were cytochrome P450 (CYP) isoforms and P-gp. All the obtained data for tested derivatives and standard drugs are listed in Table 6.

## Supplementary Material

Supplemental Material

## Data Availability

The data supporting the findings of this study are available within the article [and/or] its supplementary materials.
