# Supplementary material for: Design and synthesis of new indole drug candidates to treat Alzheimer’s disease and targeting neuro-inflammation using a multi-target-directed ligand (MTDL) strategy
Source: J Enzyme Inhib Med Chem. 2022 Sep 22;37(1):2660–78. doi: 10.1080/14756366.2022.2126464 (PMC9518246; doi:10.1080/14756366.2022.2126464)
Supplement: Supplemental Material [file IENZ_A_2126464_SM1551.pdf]

3c-Carbon

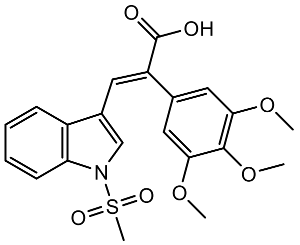
O
OH
O
O
O
N
O=S=O
O

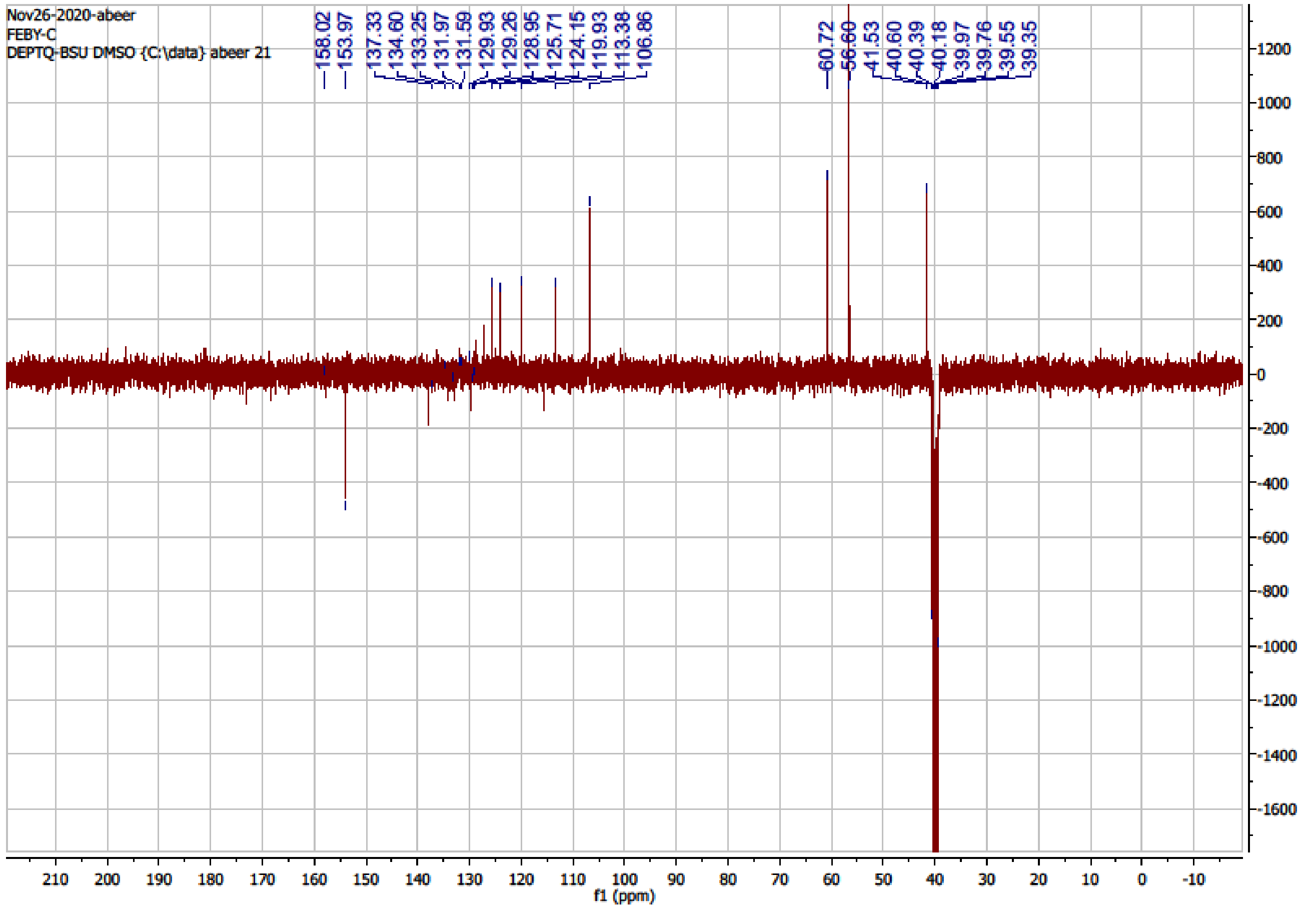
Nov26-2020-abeer
FEBY-C
DEPTQ-BSU DMSO {C:\data} abeer 21
158.02
153.97
137.33
134.60
133.25
131.97
131.59
129.93
129.26
128.95
125.71
124.15
119.93
113.38
106.86
60.72
56.60
41.53
40.60
40.39
40.18
39.97
39.76
39.55
39.35
f1 (ppm)

4b-Carbon

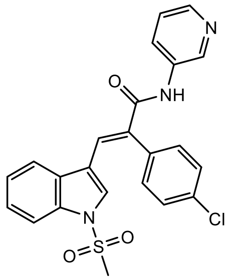
N
O
NH
N
S
O
O
Cl

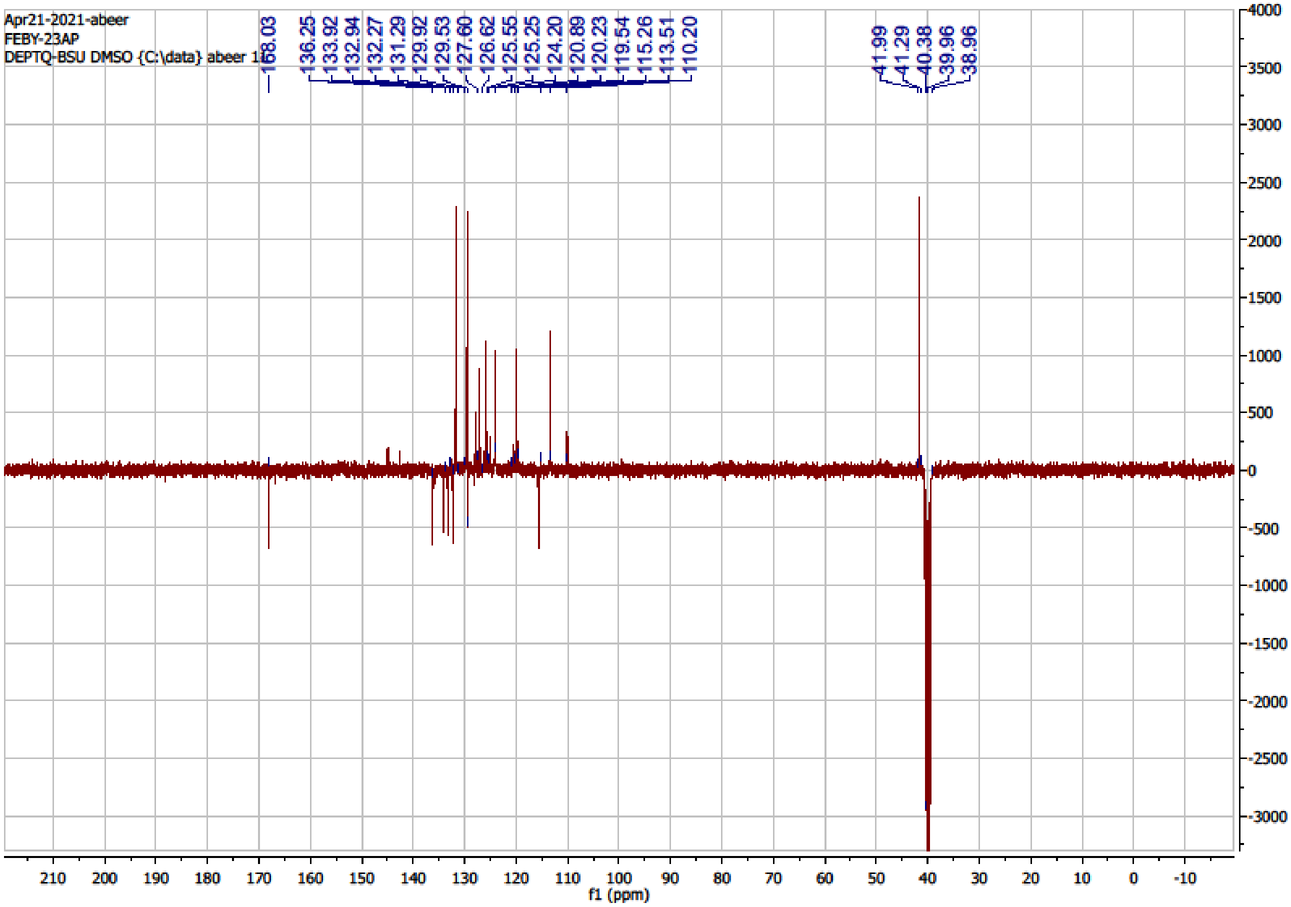
Apr21-2021-abeer
FEBY-23AP
DEPTQ-BSU DMSO {C:\data} abeer 1
168.03
136.25
133.92
132.94
132.27
131.29
129.92
129.53
127.60
126.62
125.55
125.25
124.20
120.89
120.23
119.54
115.26
113.51
110.20
41.99
41.29
40.38
39.96
38.96
f1 (ppm)

5a-Carbon

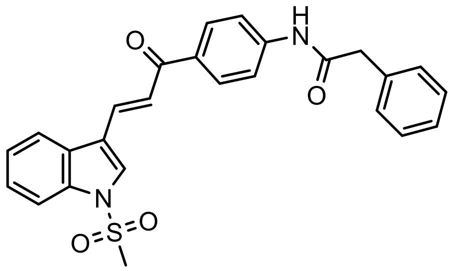

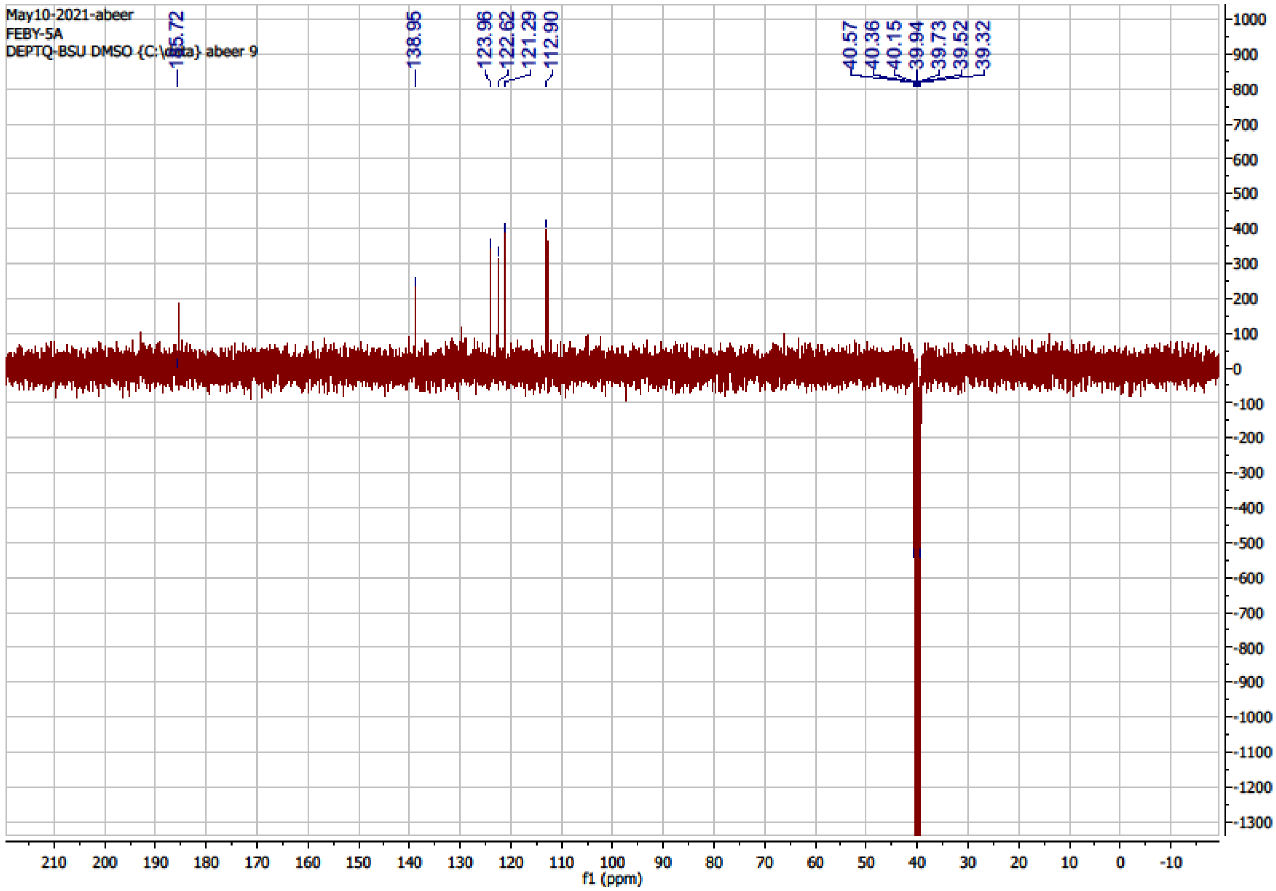

May10-2021-abeer
FEBY-5A
DEPTQ-BSU DMSO {C:\data} abeer 9
185.72
138.95
123.96
122.62
121.29
112.90
40.57
40.36
40.15
39.94
39.73
39.52
39.32
f1 (ppm)

5c-Carbon

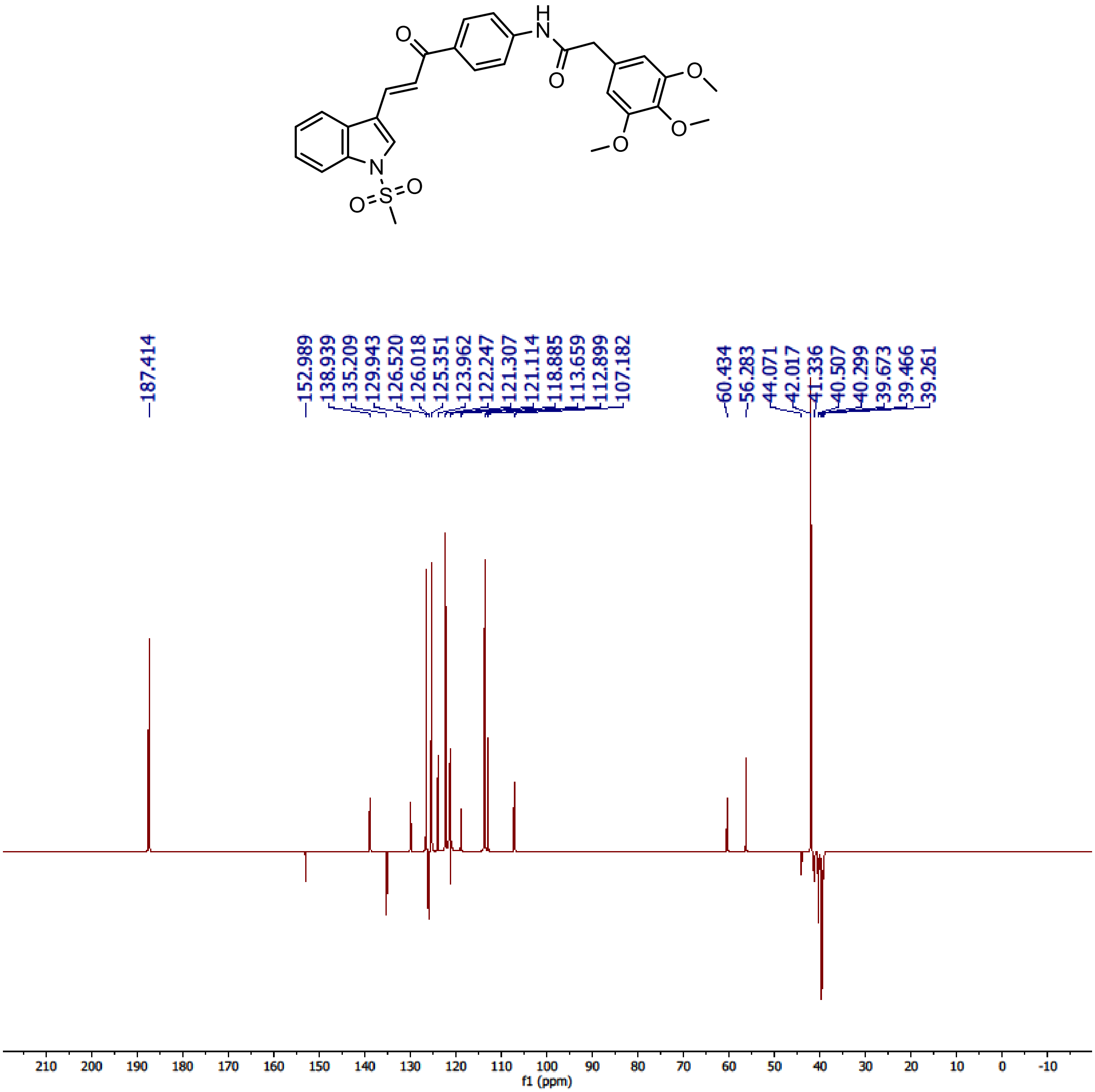

H
N
O
O
O
O
O
N
S
O
O
187.414
152.989
138.939
135.209
129.943
126.520
126.018
125.351
123.962
122.247
121.307
121.114
118.885
113.659
112.899
107.182
60.434
56.283
44.071
42.017
41.336
40.507
40.299
39.673
39.466
39.261
210 200 190 180 170 160 150 140 130 120 110 100 90 80 70 60 50 40 30 20 10 0 -10
f1 (ppm)

6a-Carbon

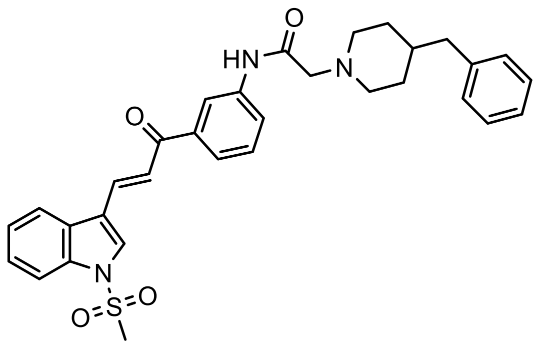
O
HN
O
N
O
N
O=S=O

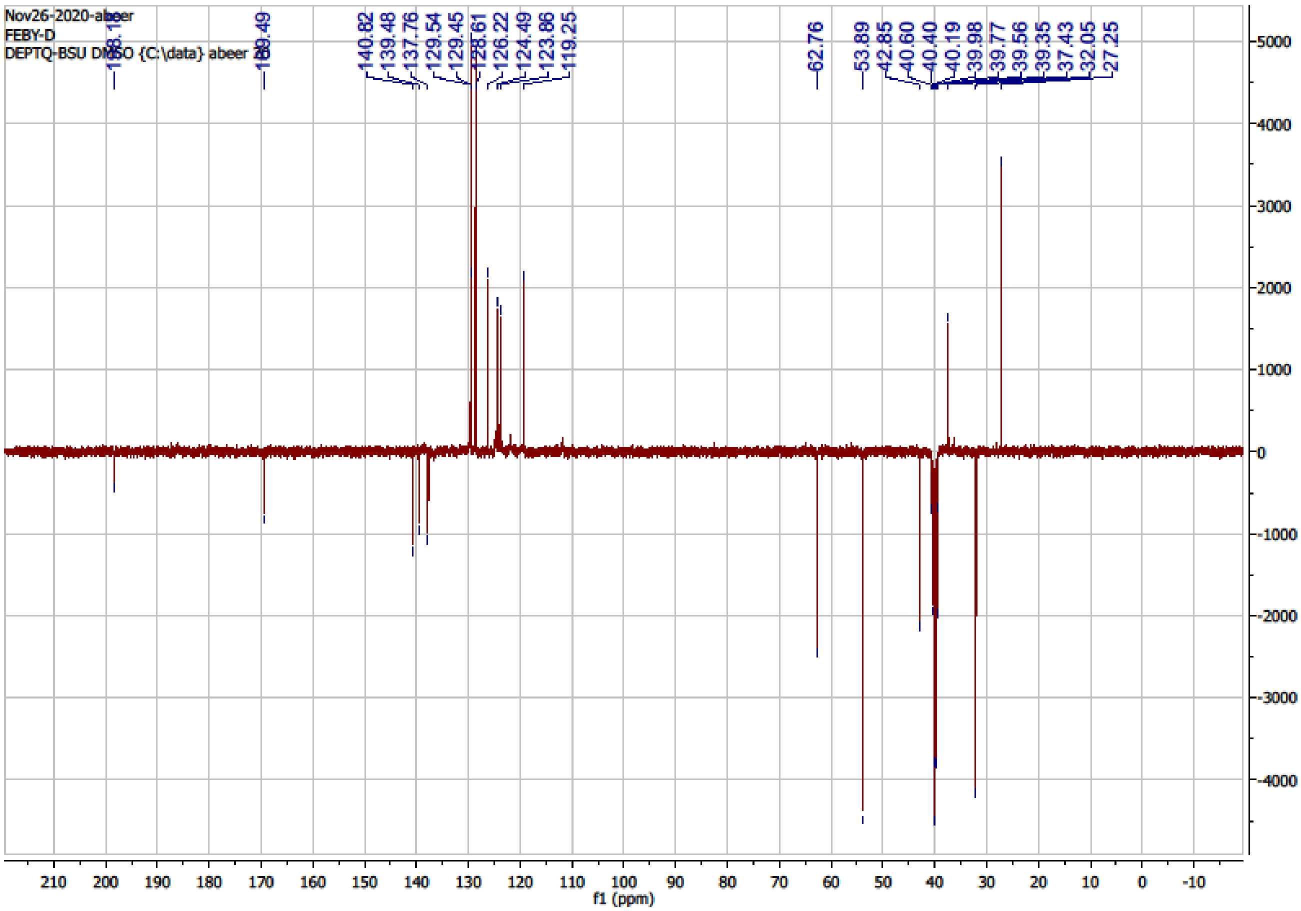
Nov26-2020-abeer
FEBY-D
DEPTQ-BSU DMSO {C:\data} abeer 26
169.49
140.82
139.48
137.76
129.54
129.45
128.61
126.22
124.49
123.86
119.25
62.76
53.89
42.85
40.60
40.40
40.19
39.98
39.77
39.56
39.35
37.43
32.05
27.25
f1 (ppm)

6b-Carbon

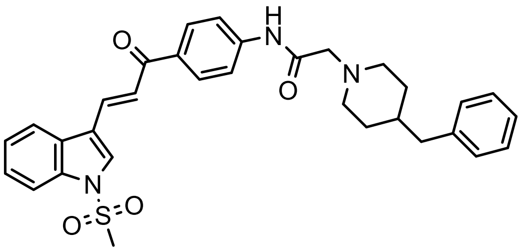

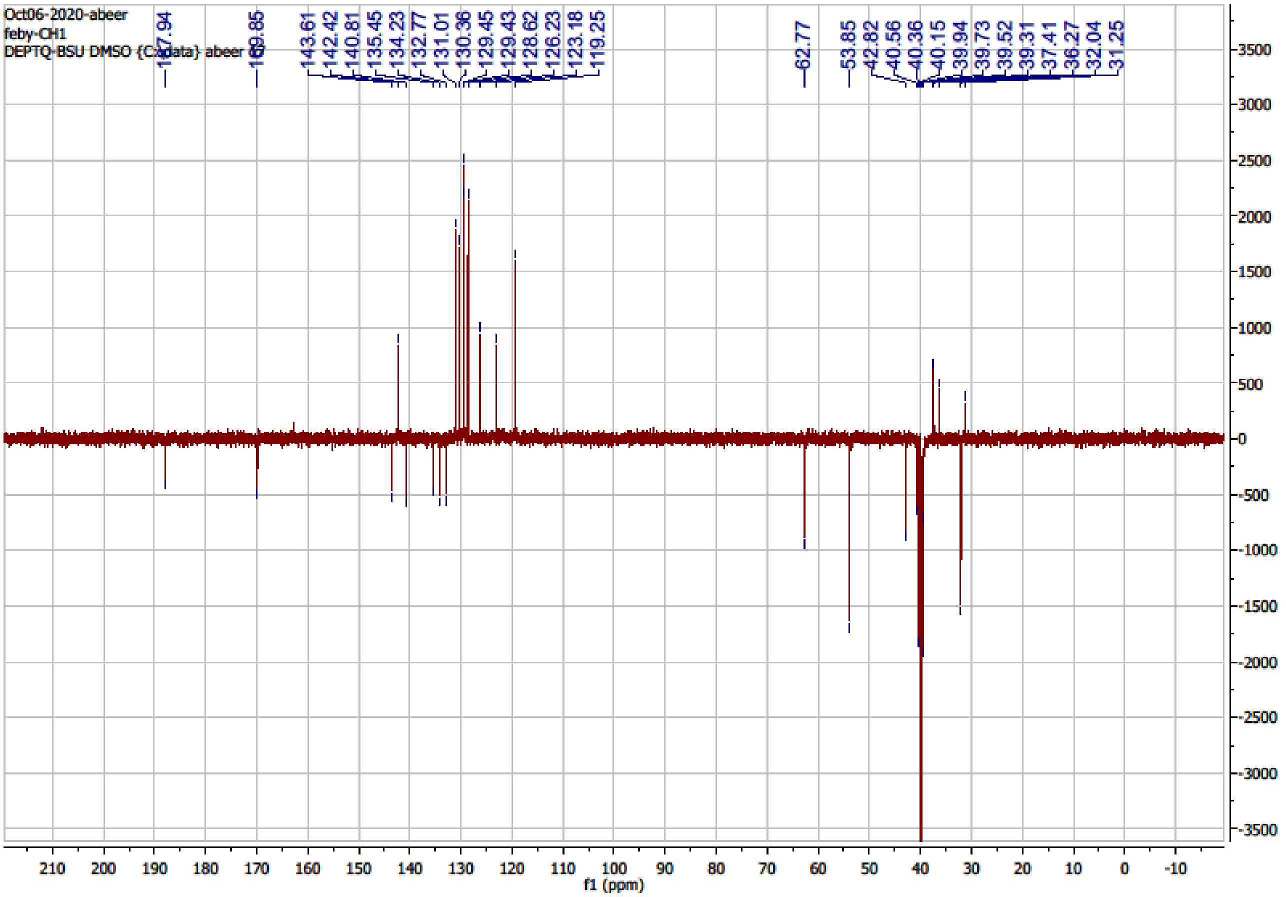

Oct06-2020-abeer
feby-CH1
DEPTQ-BSU DMSO {C:\data} abeer 6
187.94
169.85
143.61
142.42
140.81
135.45
134.23
132.77
131.01
130.36
129.45
129.43
128.62
126.23
123.18
119.25
62.77
53.85
42.82
40.56
40.36
40.15
39.94
39.73
39.52
39.31
37.41
36.27
32.04
31.25
f1 (ppm)

7c-Carbon

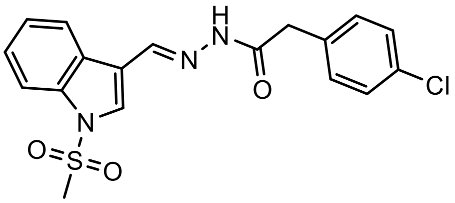

H
N
N
O
Cl
N
O
S
O

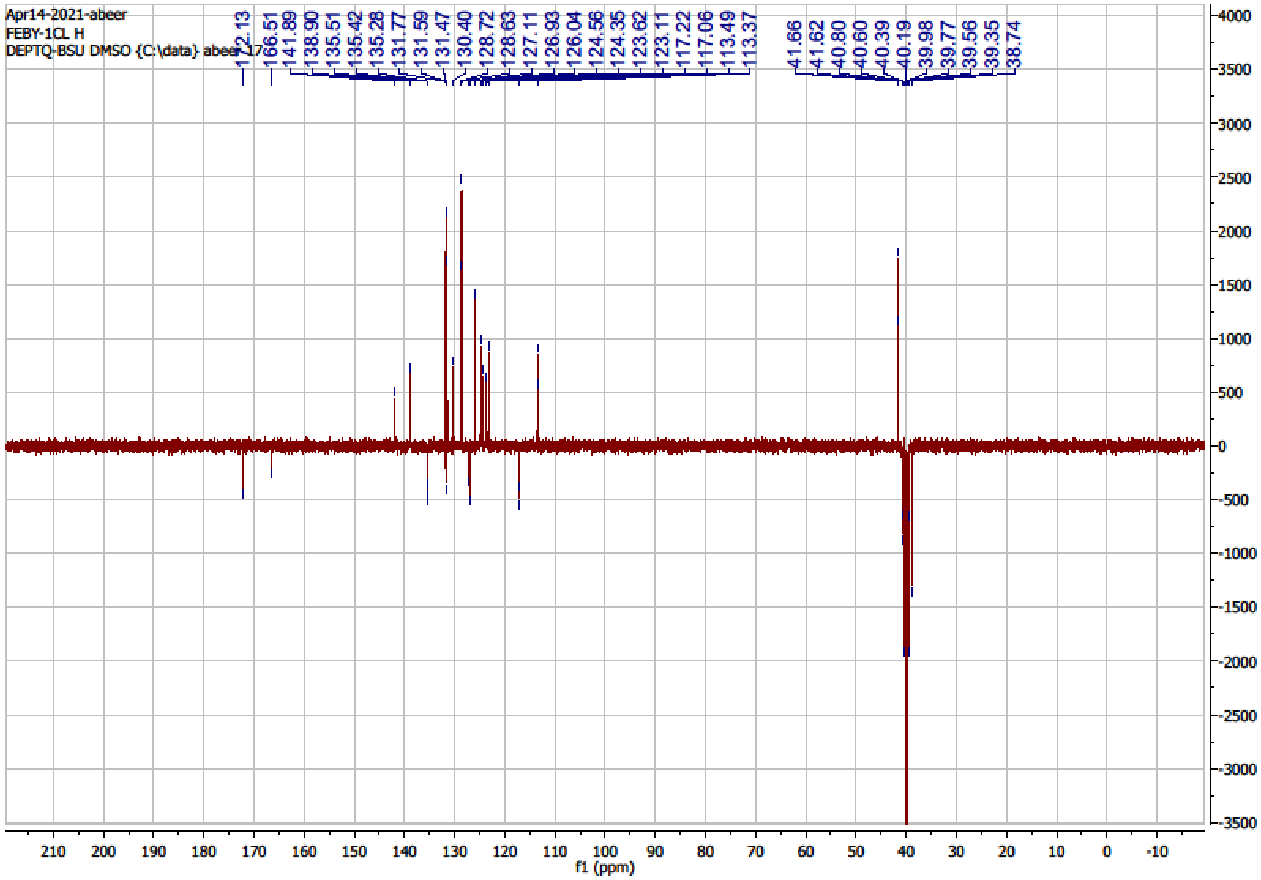

Apr14-2021-abeer
FEBY-1CL H
DEPTQ-BSU DMSO {C:\data} abeer 17
166.51
141.89
138.90
135.51
135.42
135.28
131.77
131.59
131.47
130.40
128.72
128.63
127.11
126.93
126.04
124.56
124.35
123.62
123.11
117.22
117.06
113.49
113.37
41.66
41.62
40.80
40.60
40.39
40.19
39.98
39.77
39.56
39.35
38.74
f1 (ppm)

8-Carbon

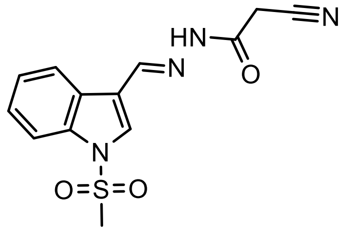
HN
N
O
N
N
O=S=O

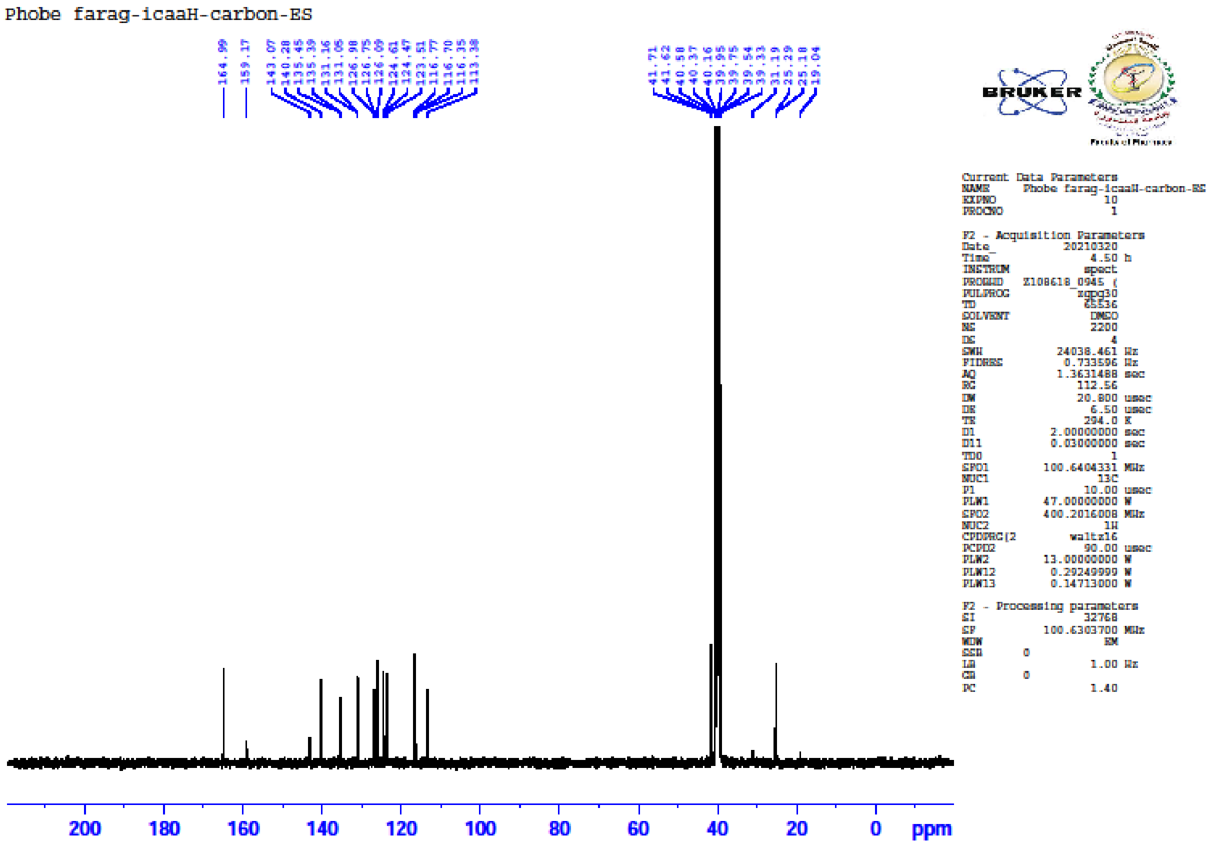
Phobe farag-icaaH-carbon-ES
164.99
159.17
143.07
140.28
135.45
135.39
131.16
131.05
126.98
126.75
126.09
124.61
124.47
123.51
116.77
116.70
116.35
113.38
41.71
41.62
40.58
40.37
40.16
39.95
39.75
39.54
39.33
31.19
25.29
25.18
19.04
200
180
160
140
120
100
80
60
40
20
0
ppm
BRUKER
Current Data Parameters
NAME Phobe farag-icaaH-carbon-ES
EXPNO 10
PROCNO 1
F2 - Acquisition Parameters
Date_ 20210320
Time 4.50 h
INSTRUM spect
PROBHD Z108618_0945 (
PULPROG zgpg30
TD 65536
SOLVENT DMSO
NS 2200
DS 4
SWH 24038.461 Hz
FIDRES 0.733596 Hz
AQ 1.3631488 sec
RG 112.56
DW 20.800 usec
DE 6.50 usec
TE 294.0 K
D1 2.00000000 sec
D11 0.03000000 sec
TD0 1
SFO1 100.6404331 MHz
NUC1 13C
P1 10.00 usec
PLW1 47.00000000 W
SFO2 400.2016008 MHz
NUC2 1H
CPDPRG[2 waltz16
PCPD2 90.00 usec
PLW2 13.00000000 W
PLW12 0.29249999 W
PLW13 0.14713000 W
F2 - Processing parameters
SI 32768
SF 100.6303700 MHz
WDW EM
SSB 0
LB 1.00 Hz
GB 0
PC 1.40

9b-Carbon

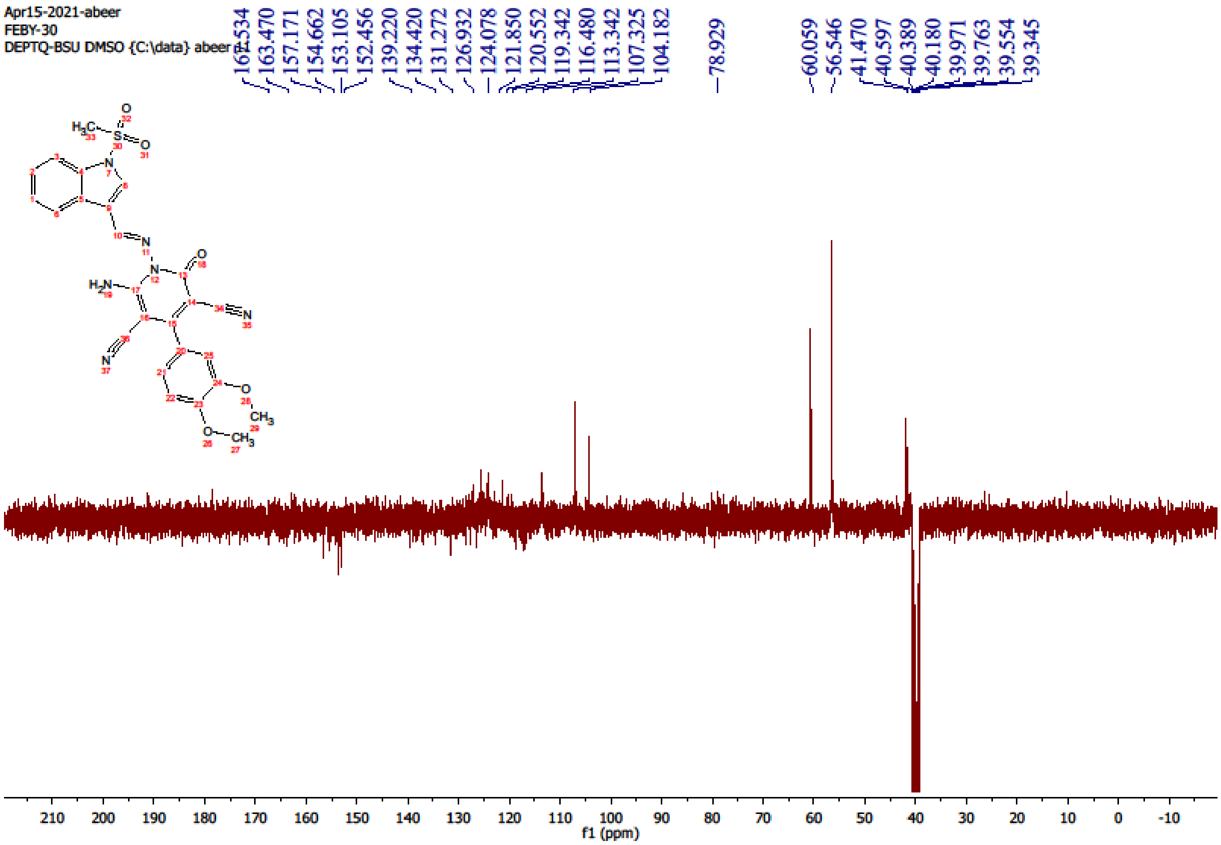

Apr15-2021-abeer
FEBY-30
DEPTQ-BSU DMSO {C:\data} abeer 11
163.470
157.171
154.662
153.105
152.456
139.220
134.420
131.272
126.932
124.078
121.850
120.552
119.342
116.480
113.342
107.325
104.182
78.929
60.059
56.546
41.470
40.597
40.389
40.180
39.971
39.763
39.554
39.345
f1 (ppm)

Ca-Carbon

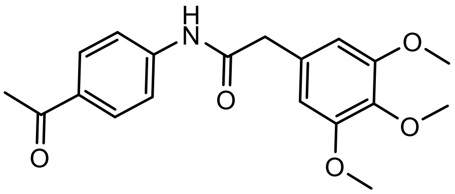

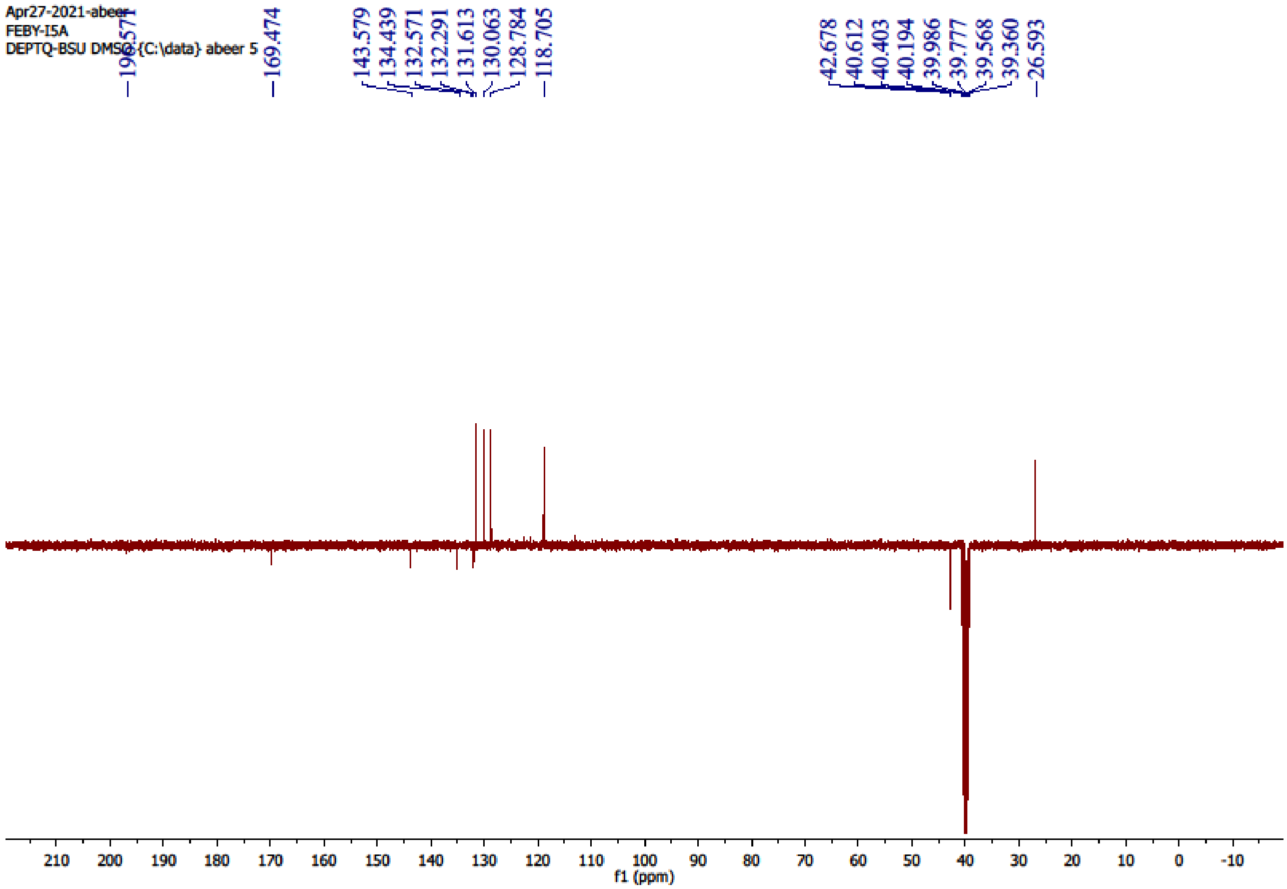
Apr27-2021-abeer
FEBY-I5A
DEPTQ-BSU DMSO {C:\data} abeer 5
196.571
169.474
143.579
134.439
132.571
132.291
131.613
130.063
128.784
118.705
42.678
40.612
40.403
40.194
39.986
39.777
39.568
39.360
26.593
210 200 190 180 170 160 150 140 130 120 110 100 90 80 70 60 50 40 30 20 10 0 -10
f1 (ppm)

Fa-Carbon

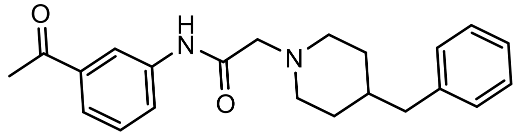
O
H
N
N
O

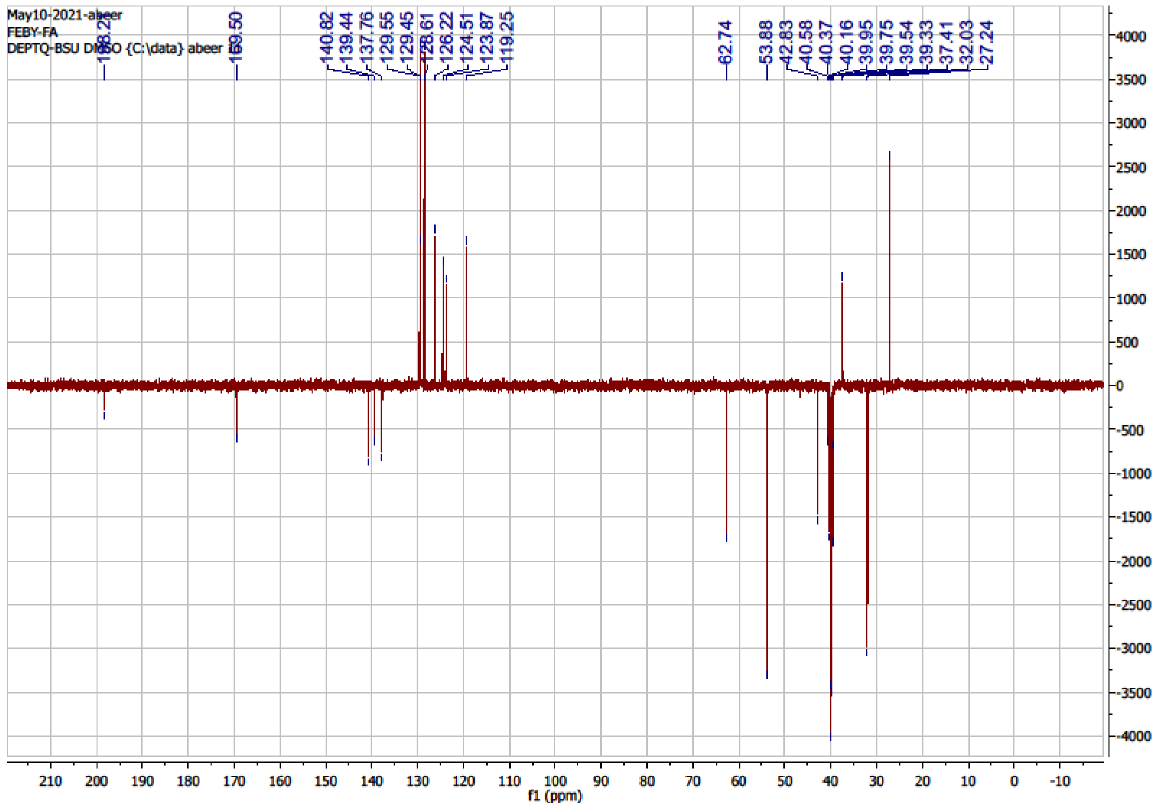
May10-2021-abeer
FEBY-FA
DEPTQ-BSU DMSO {C:\data} abeer 16
198.21
169.50
140.82
139.44
137.76
129.55
129.45
128.61
126.22
124.51
123.87
119.25
62.74
53.88
42.83
40.58
40.37
40.16
39.95
39.75
39.54
39.33
37.41
32.03
27.24
f1 (ppm)

IR-Data

3a

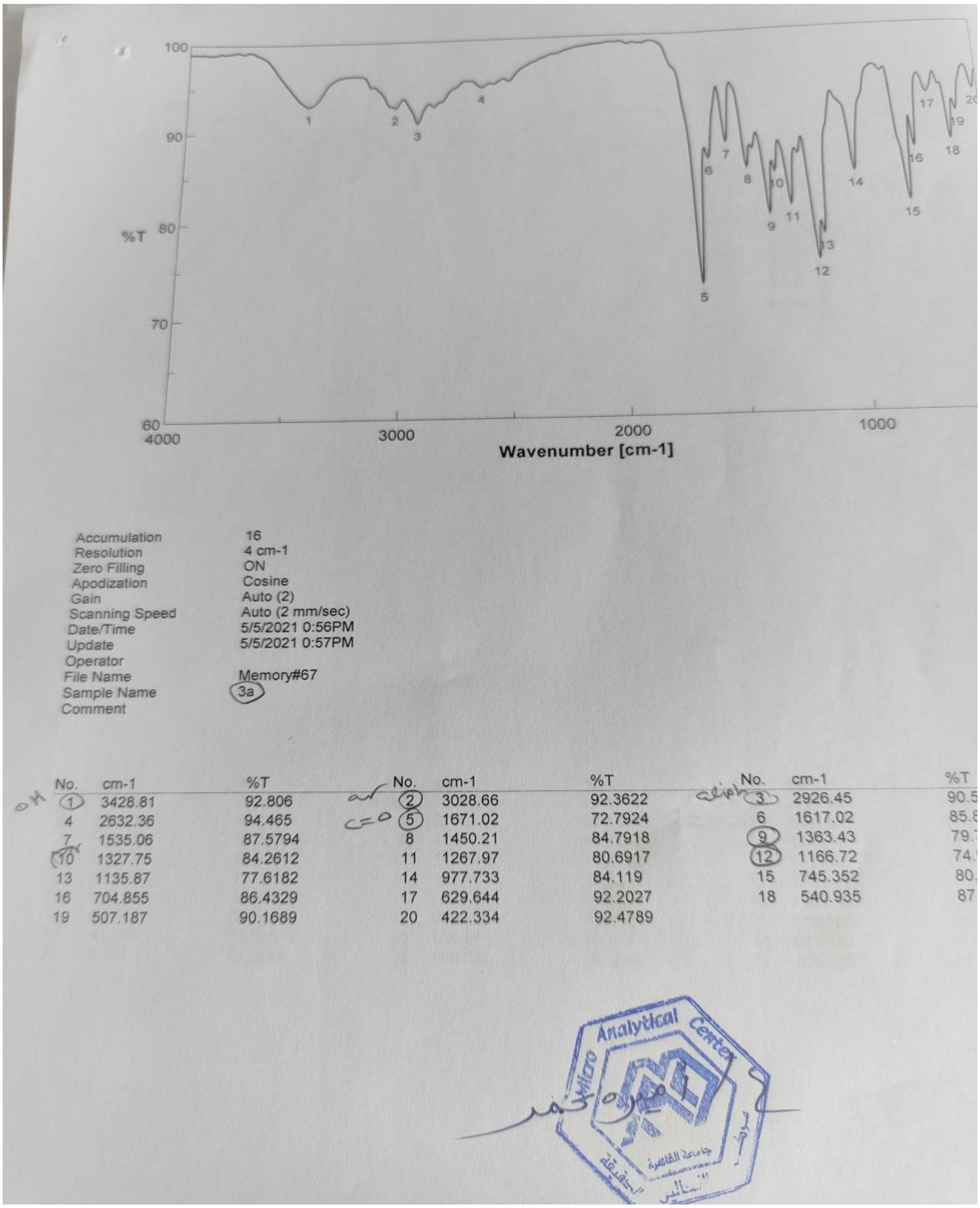

Accumulation	16
Resolution	4 cm-1
Zero Filling	ON
Apodization	Cosine
Gain	Auto (2)
Scanning Speed	Auto (2 mm/sec)
Date/Time	5/5/2021 0:56PM
Update	5/5/2021 0:57PM
Operator	
File Name	Memory#67
Sample Name	3a
Comment	

No.	cm-1	%T	No.	cm-1	%T	No.	cm-1	%T
1	3428.81	92.806	2	3028.66	92.3622	3	2926.45	90.5[illegible]
4	2632.36	94.465	5	1671.02	72.7924	6	1617.02	85.[illegible]
7	1535.06	87.5794	8	1450.21	84.7918	9	1363.43	79.[illegible]
10	1327.75	84.2612	11	1267.97	80.6917	12	1166.72	74.[illegible]
13	1135.87	77.6182	14	977.733	84.119	15	745.352	80.[illegible]
16	704.855	86.4329	17	629.644	92.2027	18	540.935	87[illegible]
19	507.187	90.1689	20	422.334	92.4789			

4b

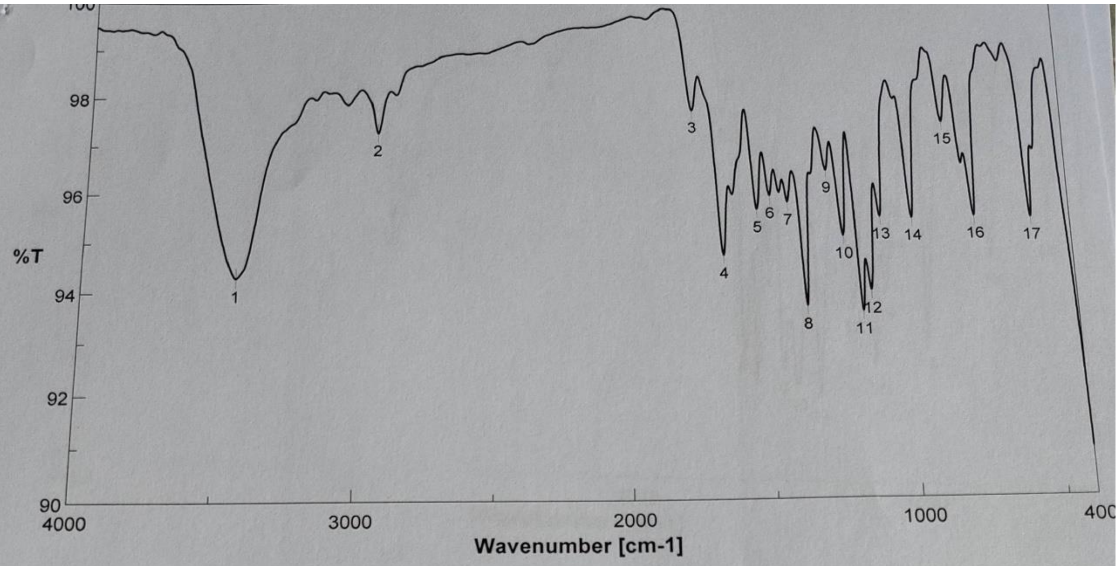

Accumulation	16
Resolution	4 cm-1
Zero Filling	ON
Apodization	Cosine
Gain	Auto (2)
Scanning Speed	Auto (2 mm/sec)
Date/Time	5/5/2021 1:22PM
Update	5/5/2021 1:25PM
Operator	
File Name	Memory#148
Sample Name	4b
Comment	

No.	cm-1	%T	No.	cm-1	%T	No.	cm-1	%T
1	3432.67	94.2779	2	2924.52	97.1856	3	1755.87	97.5792
4	1658.48	94.6488	5	1532.17	95.5415	6	1484.92	95.8098
7	1420.32	95.6733	8	1366.32	93.6258	9	1275.68	96.305
10	1225.54	94.9872	11	1168.65	93.5048	12	1135.87	93.9022
13	1088.62	95.3587	14	973.876	95.3206	15	837.919	97.2517
16	745.352	95.3478	17	539.971	95.3106			

Micro Analytical Center

5a

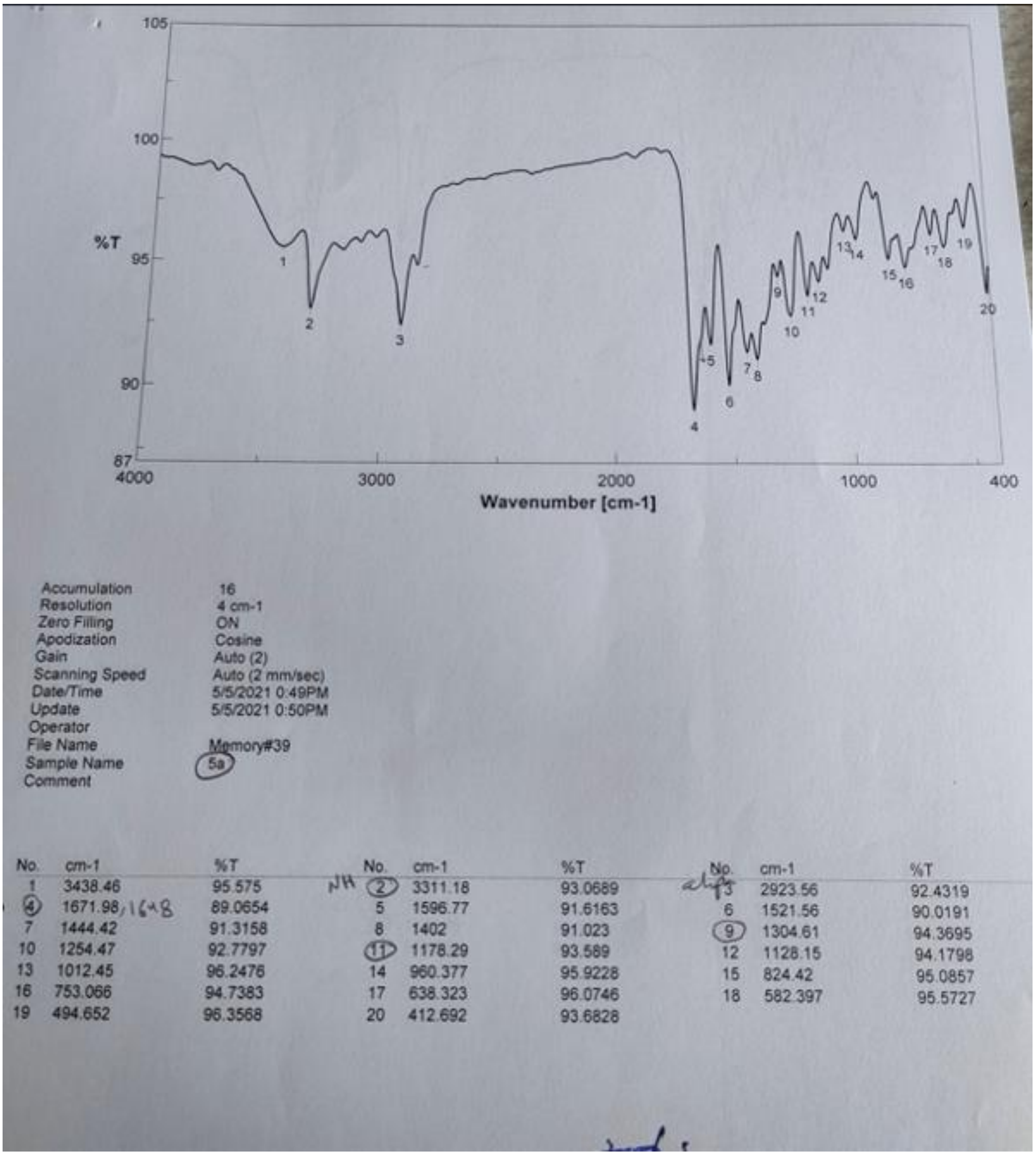

Accumulation	16
Resolution	4 cm-1
Zero Filling	ON
Apodization	Cosine
Gain	Auto (2)
Scanning Speed	Auto (2 mm/sec)
Date/Time	5/5/2021 0:49PM
Update	5/5/2021 0:50PM
Operator	
File Name	Memory#39
Sample Name	5a
Comment	

No.	cm-1	%T	No.	cm-1	%T	No.	cm-1	%T
1	3438.46	95.575	NH 2	3311.18	93.0689	alip 3	2923.56	92.4319
4	1671.98, 1648	89.0654	5	1596.77	91.6163	6	1521.56	90.0191
7	1444.42	91.3158	8	1402	91.023	9	1304.61	94.3695
10	1254.47	92.7797	11	1178.29	93.589	12	1128.15	94.1798
13	1012.45	96.2476	14	960.377	95.9228	15	824.42	95.0857
16	753.066	94.7383	17	638.323	96.0746	18	582.397	95.5727
19	494.652	96.3568	20	412.692	93.6828			

5c

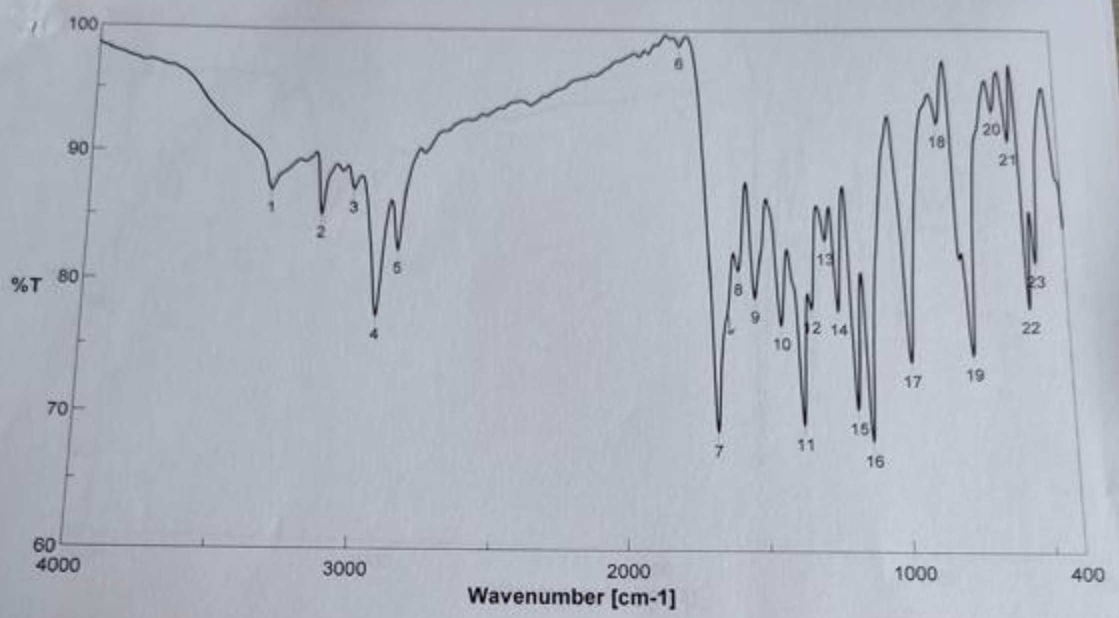

Accumulation	16
Resolution	4 cm-1
Zero Filling	ON
Apodization	Cosine
Gain	Auto (2)
Scanning Speed	Auto (2 mm/sec)
Date/Time	5/5/2021 1:45PM
Update	5/5/2021 1:47PM
Operator	
File Name	Memory#184
Sample Name	5c
Comment	

No.	cm-1	%T	No.	cm-1	%T	No.	cm-1	%T
1	3324.68	87.0364	2	3136.65	85.0991	3	3019.01	87.0936
4	2926.45	77.273	5	2850.27	82.3839	6	1801.19	98.774
7	1676.8	68.6416, 1648 cm	8	1594.84	80.8282	9	1535.06	78.736
10	1443.46	76.6577	11	1368.25	69.1618	12	1332.57	77.9393
13	1275.68	83.2398	14	1234.22	77.802	15	1174.44	70.3848
16	1125.26	68.0073	17	975.804	73.9388	18	845.633	92.4437
19	749.209	74.4658	20	637.358	93.4282	21	583.361	90.984
22	538.042	78.0129	23	507.187	81.4978			

6a

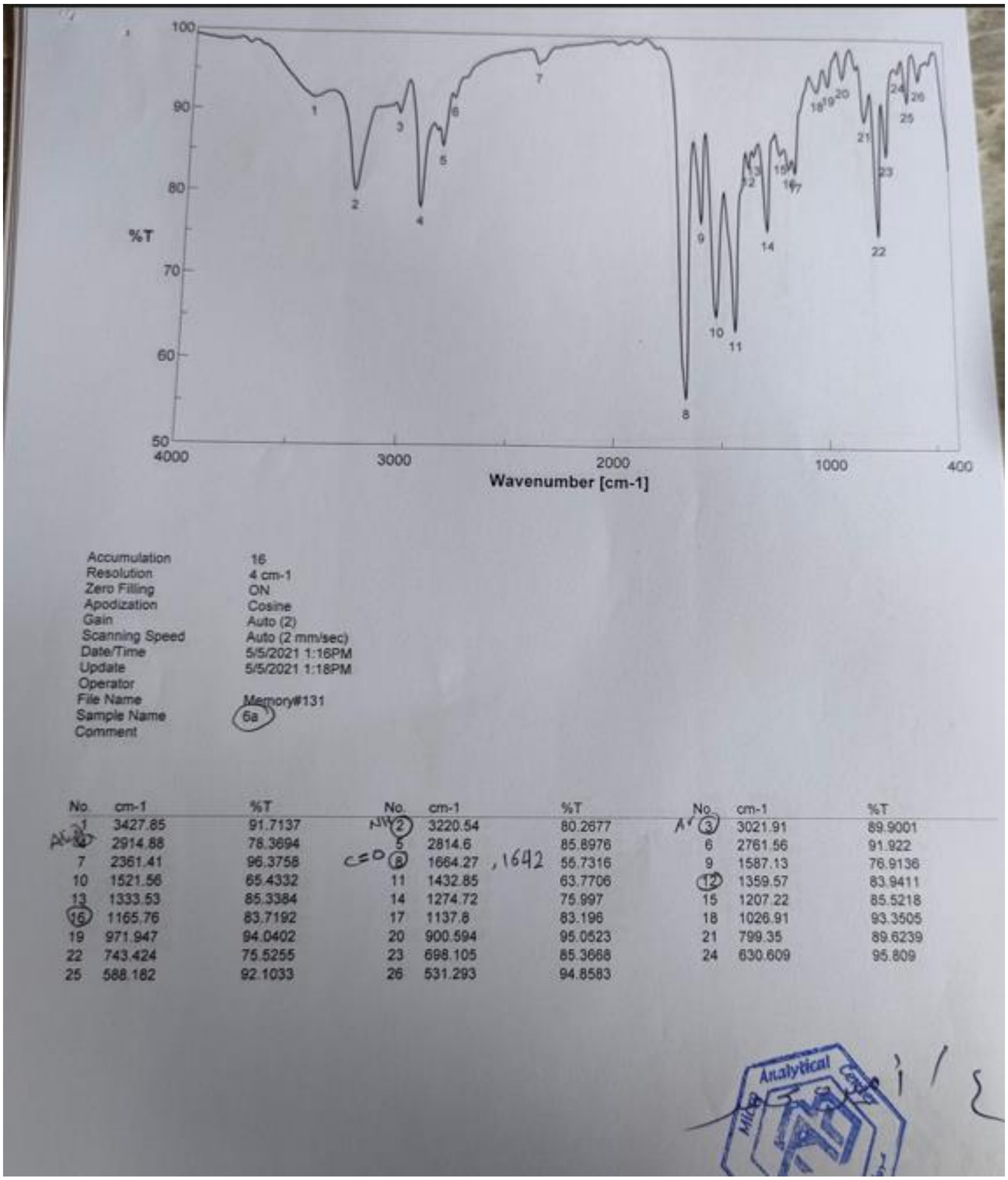

Accumulation	16
Resolution	4 cm-1
Zero Filling	ON
Apodization	Cosine
Gain	Auto (2)
Scanning Speed	Auto (2 mm/sec)
Date/Time	5/5/2021 1:16PM
Update	5/5/2021 1:18PM
Operator	
File Name	Memory#131
Sample Name	6a
Comment	

No.	cm-1	%T	No.	cm-1	%T	No.	cm-1	%T
1	3427.85	91.7137	2	3220.54	80.2677	3	3021.91	89.9001
[illegible]	2914.88	78.3694	5	2814.6	85.8976	6	2761.56	91.922
7	2361.41	96.3758	8	1664.27	55.7316	9	1587.13	76.9136
10	1521.56	65.4332	11	1432.85	63.7706	12	1359.57	83.9411
13	1333.53	85.3384	14	1274.72	75.997	15	1207.22	85.5218
16	1165.76	83.7192	17	1137.8	83.196	18	1026.91	93.3505
19	971.947	94.0402	20	900.594	95.0523	21	799.35	89.6239
22	743.424	75.5255	23	698.105	85.3668	24	630.609	95.809
25	588.182	92.1033	26	531.293	94.8583			

Analytical

7a

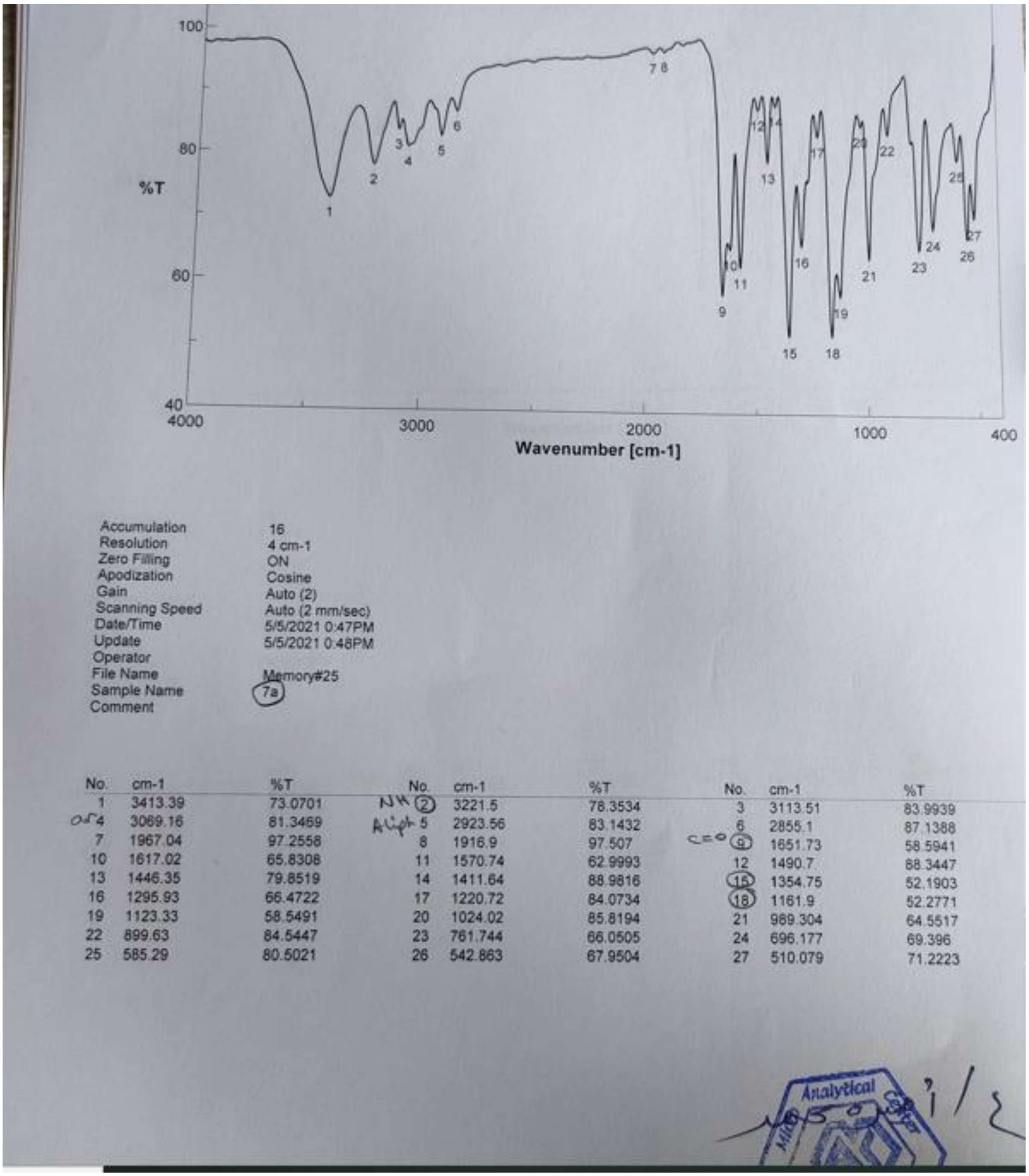

Accumulation	16
Resolution	4 cm-1
Zero Filling	ON
Apodization	Cosine
Gain	Auto (2)
Scanning Speed	Auto (2 mm/sec)
Date/Time	5/5/2021 0:47PM
Update	5/5/2021 0:48PM
Operator	
File Name	Memory#25
Sample Name	7a
Comment	

No.	cm-1	%T	No.	cm-1	%T	No.	cm-1	%T
1	3413.39	73.0701	NH 2	3221.5	78.3534	3	3113.51	83.9939
4	3069.16	81.3469	Aliph 5	2923.56	83.1432	6	2855.1	87.1388
7	1967.04	97.2558	8	1916.9	97.507	C=O 9	1651.73	58.5941
10	1617.02	65.8308	11	1570.74	62.9993	12	1490.7	88.3447
13	1446.35	79.8519	14	1411.64	88.9816	15	1354.75	52.1903
16	1295.93	66.4722	17	1220.72	84.0734	18	1161.9	52.2771
19	1123.33	58.5491	20	1024.02	85.8194	21	989.304	64.5517
22	899.63	84.5447	23	761.744	66.0505	24	696.177	69.396
25	585.29	80.5021	26	542.863	67.9504	27	510.079	71.2223

Analytical

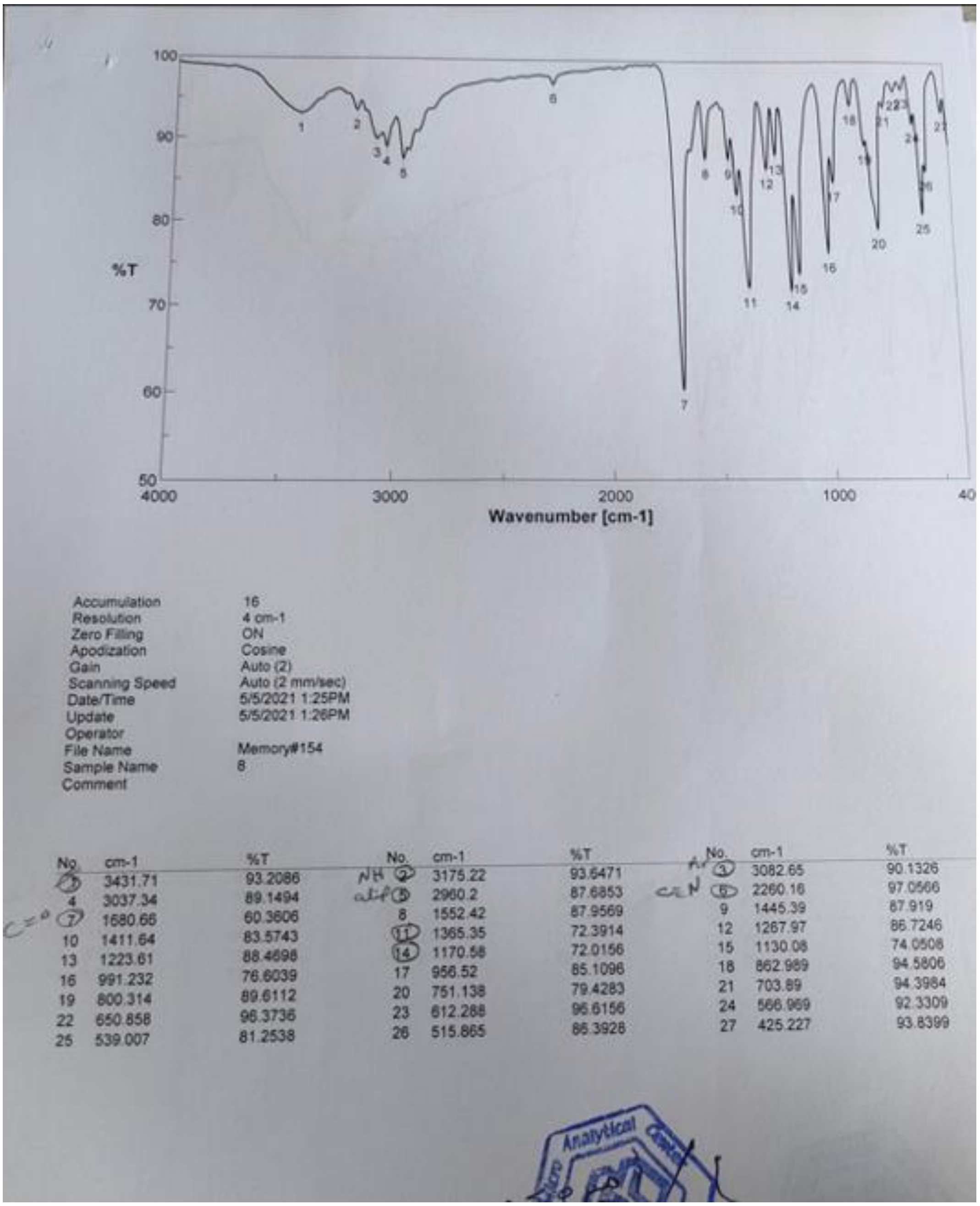

Accumulation	16
Resolution	4 cm-1
Zero Filling	ON
Apodization	Cosine
Gain	Auto (2)
Scanning Speed	Auto (2 mm/sec)
Date/Time	5/5/2021 1:25PM
Update	5/5/2021 1:26PM
Operator	
File Name	Memory#154
Sample Name	8
Comment	

No.	cm-1	%T	No.	cm-1	%T	No.	cm-1	%T
1	3431.71	93.2086	2	3175.22	93.6471	3	3082.65	90.1326
4	3037.34	89.1494	5	2960.2	87.6853	6	2260.16	97.0566
7	1680.66	60.3606	8	1552.42	87.9569	9	1445.39	87.919
10	1411.64	83.5743	11	1365.35	72.3914	12	1267.97	86.7246
13	1223.61	88.4698	14	1170.58	72.0156	15	1130.08	74.0508
16	991.232	76.6039	17	956.52	85.1096	18	862.989	94.5806
19	800.314	89.6112	20	751.138	79.4283	21	703.89	94.3984
22	650.858	96.3736	23	612.288	96.6156	24	566.969	92.3309
25	539.007	81.2538	26	515.865	86.3928	27	425.227	93.8399

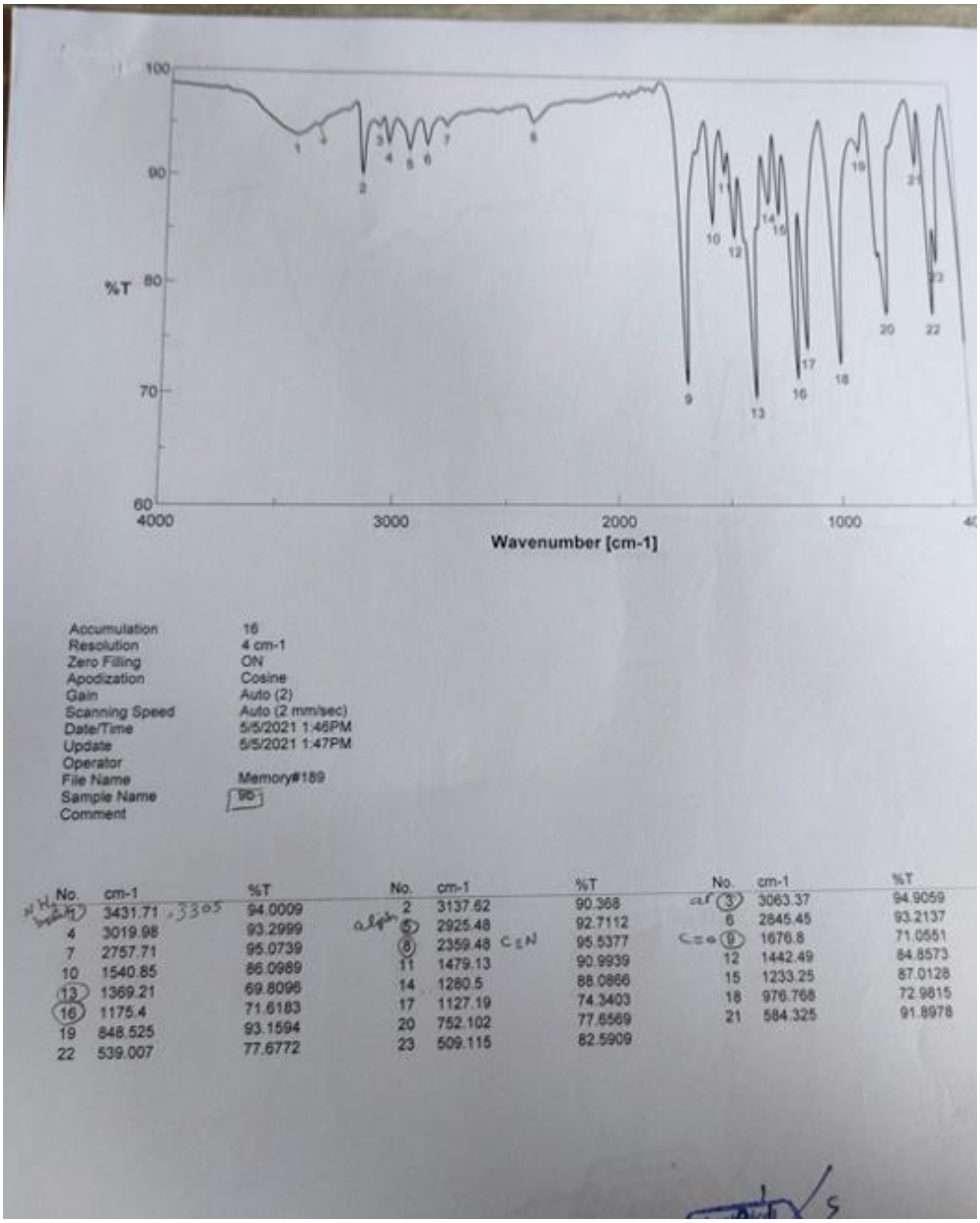

Accumulation	16
Resolution	4 cm-1
Zero Filling	ON
Apodization	Cosine
Gain	Auto (2)
Scanning Speed	Auto (2 mm/sec)
Date/Time	5/5/2021 1:46PM
Update	5/5/2021 1:47PM
Operator	
File Name	Memory#189
Sample Name	9b
Comment	

No.	cm-1	%T	No.	cm-1	%T	No.	cm-1	%T
1	3431.71	94.0009	2	3137.62	90.368	3	3063.37	94.9059
4	3019.98	93.2999	5	2925.48	92.7112	6	2845.45	93.2137
7	2757.71	95.0739	8	2359.48	95.5377	9	1676.8	71.0551
10	1540.85	86.0989	11	1479.13	90.9939	12	1442.49	84.8573
13	1369.21	69.8096	14	1260.5	88.0866	15	1233.25	87.0128
16	1175.4	71.6183	17	1127.19	74.3403	18	976.768	72.9815
19	848.525	93.1594	20	752.102	77.6569	21	584.325	91.8978
22	539.007	77.6772	23	509.115	82.5909			

10a

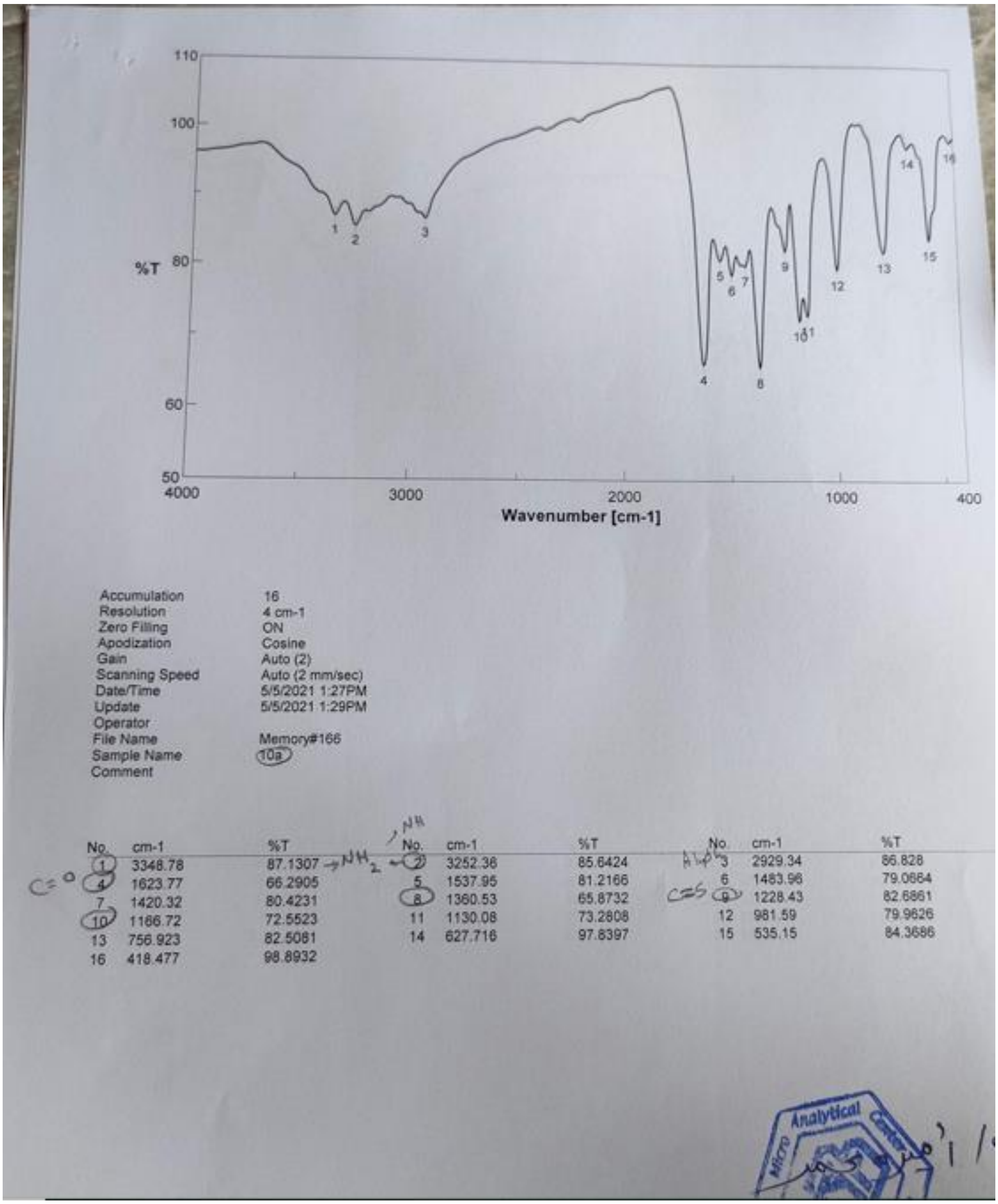

Accumulation	16
Resolution	4 cm-1
Zero Filling	ON
Apodization	Cosine
Gain	Auto (2)
Scanning Speed	Auto (2 mm/sec)
Date/Time	5/5/2021 1:27PM
Update	5/5/2021 1:29PM
Operator	
File Name	Memory#166
Sample Name	10a
Comment	

No.	cm-1	%T	No.	cm-1	%T	No.	cm-1	%T
1	3348.78	87.1307	2	3252.36	85.6424	3	2929.34	86.828
4	1623.77	66.2905	5	1537.95	81.2166	6	1483.96	79.0664
7	1420.32	80.4231	8	1360.53	65.8732	9	1228.43	82.6861
10	1166.72	72.5523	11	1130.08	73.2808	12	981.59	79.9626
13	756.923	82.5081	14	627.716	97.8397	15	535.15	84.3686
16	418.477	98.8932						

C=O → NH_2 NH Alph C=S

Analytical Center

Mass Data

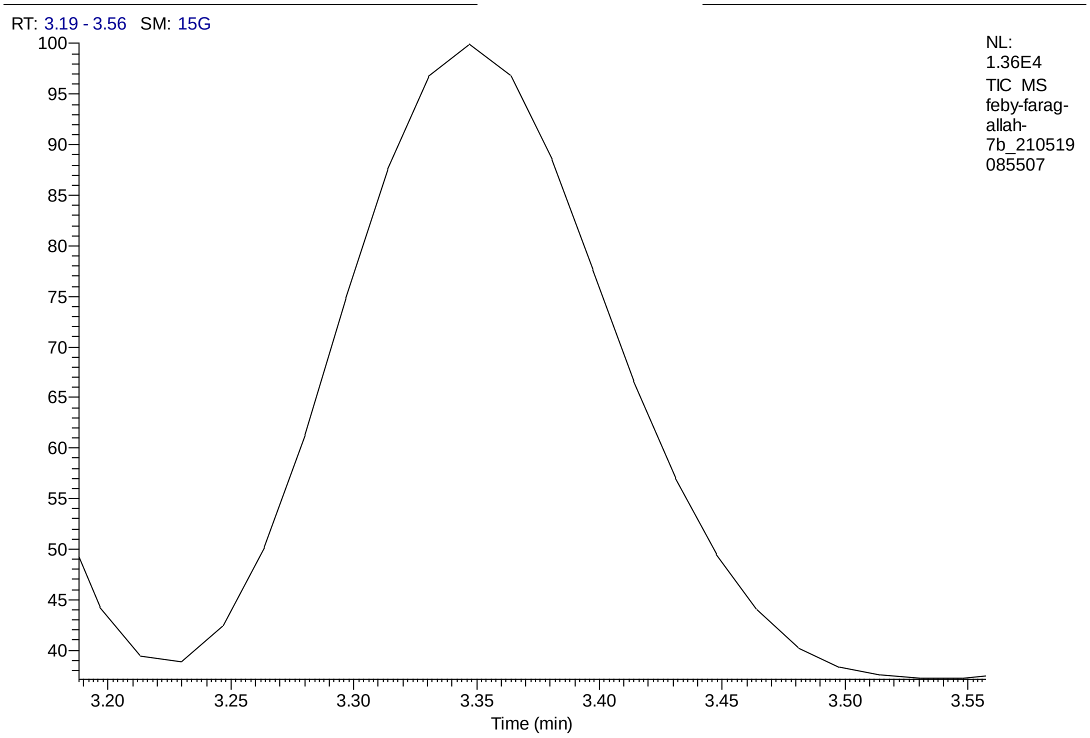

RT: 3.19 - 3.56 SM: 15G
NL: 1.36E4
TIC MS
feby-farag-allah-7b_210519085507
Time (min)

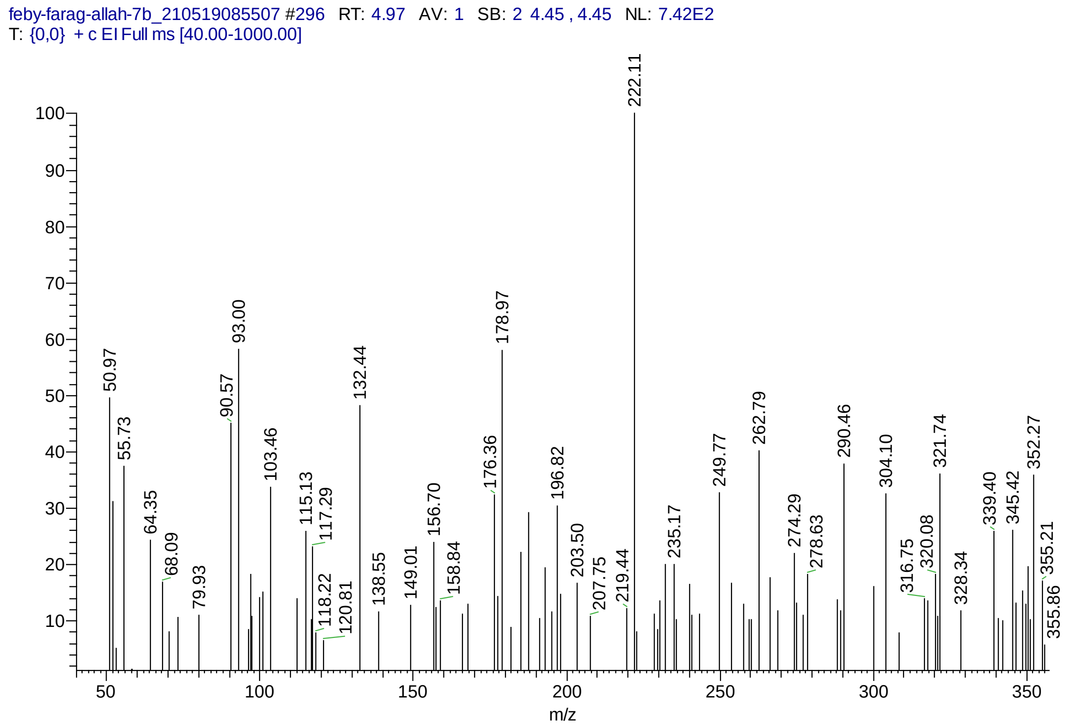

feby-farag-allah-7b_210519085507 #296 RT: 4.97 AV: 1 SB: 2 4.45 , 4.45 NL: 7.42E2
T: {0,0} + c EI Full ms [40.00-1000.00]
222.11
178.97
93.00
132.44
50.97
90.57
m/z

m/z	Intensity	Relative
50.97	368.2	49.64
52.08	231.5	31.20
53.29	38.1	5.14
55.73	278.1	37.49
58.28	9.7	1.31
64.35	180.9	24.39
68.09	125.3	16.89
70.24	60.1	8.11
73.41	79.2	10.68
79.93	81.1	10.93
90.57	334.8	45.13
93.00	432.0	58.23
96.27	63.5	8.55
96.88	135.5	18.26
97.48	80.5	10.86
99.82	105.1	14.16
100.98	112.3	15.13
103.46	250.8	33.81
112.02	103.6	13.96
115.13	192.4	25.93
116.74	76.3	10.28
117.29	171.2	23.08
118.22	57.7	7.78

120.81	47.7	6.43
132.44	357.7	48.22
138.55	86.4	11.65
149.01	94.9	12.80
156.70	176.9	23.85
157.21	91.3	12.31
158.84	100.5	13.55
165.89	82.7	11.14
167.81	96.8	13.05
176.36	239.7	32.31
177.62	106.3	14.32
178.97	429.6	57.91
182.01	65.5	8.82
185.04	164.7	22.20
187.48	216.5	29.19
191.35	77.9	10.50
193.14	144.8	19.52
195.28	86.1	11.61
196.82	226.1	30.48
197.94	109.7	14.79
203.50	123.5	16.64
207.75	80.1	10.80
219.44	90.8	12.24
222.11	741.8	100.00
222.94	60.3	8.12

228.68	82.5	11.13
229.72	62.5	8.43
230.27	100.3	13.52
232.02	148.3	19.99
235.17	149.1	20.09
235.81	75.6	10.19
240.21	121.9	16.43
240.75	81.7	11.02
243.27	83.2	11.21
249.77	243.2	32.78
253.81	124.1	16.73
257.68	96.8	13.05
259.47	76.1	10.26
260.27	76.5	10.32
262.79	298.1	40.19
266.21	130.4	17.58
268.67	86.9	11.72
274.29	163.3	22.02
274.81	97.5	13.14
277.28	81.7	11.02
278.63	136.0	18.33
288.18	101.3	13.66
289.27	87.9	11.84
290.46	281.3	37.92
300.23	118.8	16.01

304.10	241.6	32.57
308.21	58.9	7.94
316.75	104.1	14.04
317.85	100.7	13.57
320.08	135.7	18.30
320.81	80.1	10.80
321.74	267.0	36.00
328.34	87.1	11.74
339.40	192.5	25.95
340.67	77.9	10.50
342.30	74.1	9.99
345.42	194.1	26.17
346.36	98.3	13.25
348.70	113.2	15.26
349.74	96.4	12.99
350.61	145.5	19.61
351.26	75.6	10.19
352.27	265.6	35.80
355.21	127.2	17.15

355.86	41.7	5.63

RT: 3.06 - 3.84 SM: 15G

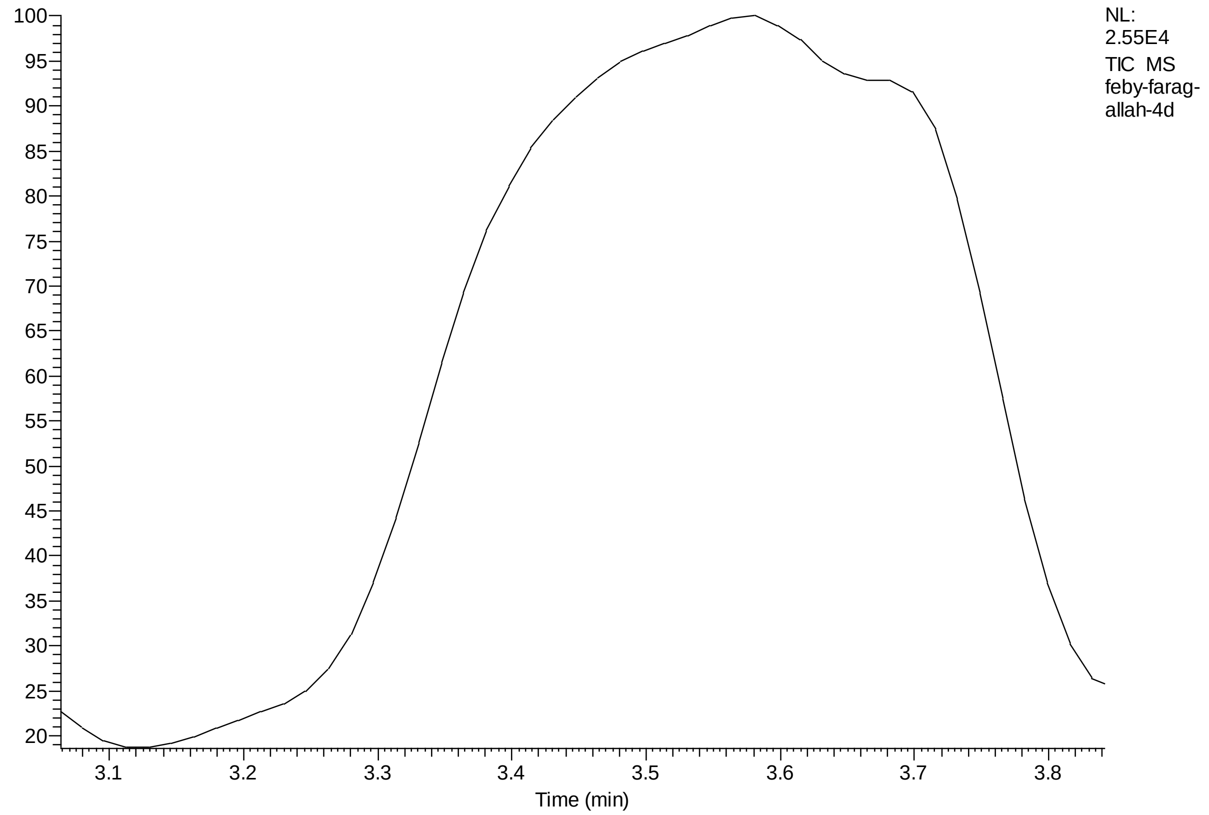

feby-farag-allah-4d #202 RT: 3.40 AV: 1 SB: 2 4.45 , 4.45 NL: 3.65E2
T: {0,0} + c EI Full ms [40.00-1000.00]

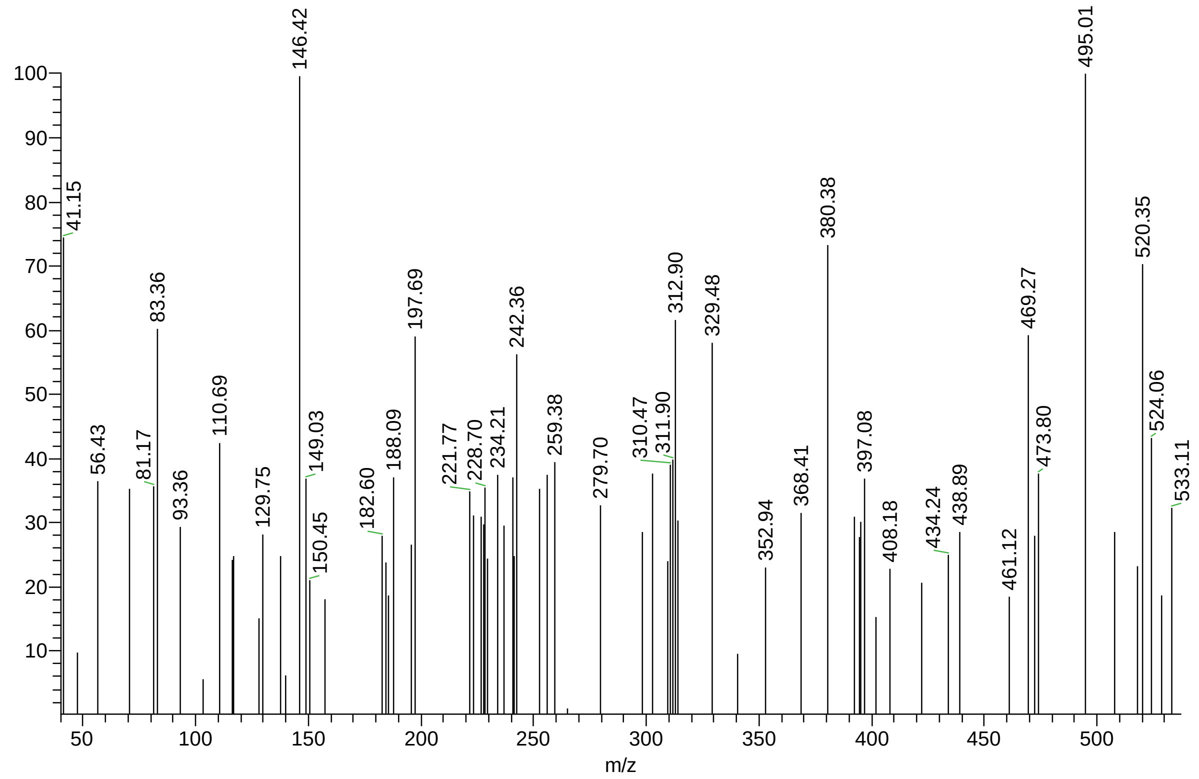

m/z	Intensity	Relative
41.15	270.9	74.27
47.43	35.3	9.69
56.43	132.8	36.40
70.77	128.4	35.20
81.17	130.0	35.64
83.36	219.3	60.12
93.36	106.5	29.20
103.41	20.0	5.48
110.69	154.4	42.32
116.19	87.7	24.05
116.83	89.7	24.60
128.08	54.4	14.91
129.75	102.0	27.96
137.96	90.1	24.71
140.10	21.9	5.99
146.42	363.2	99.56
149.03	134.4	36.84
150.45	76.3	20.91
157.47	65.5	17.95
182.60	101.5	27.81
184.57	86.1	23.61
185.56	67.3	18.46
188.09	134.5	36.88

195.54	96.3	26.39
197.69	215.3	59.03
221.77	126.5	34.69
223.09	113.5	31.10
226.75	112.3	30.77
227.61	107.9	29.57
228.70	129.2	35.42
229.39	88.8	24.34
234.21	136.0	37.28
237.14	107.6	29.50
240.55	134.8	36.95
241.15	90.3	24.74
242.36	205.1	56.21
252.54	128.1	35.12
256.15	136.4	37.39
259.38	143.7	39.40
265.26	3.3	0.91
279.70	119.2	32.68
298.39	103.7	28.44
303.01	137.3	37.65
309.27	86.9	23.83
310.47	142.0	38.93
311.90	145.2	39.80
312.90	224.1	61.44
313.95	110.3	30.23

329.48	211.2	57.89
340.52	34.5	9.47
352.94	83.5	22.88
368.41	114.9	31.51
380.38	267.0	73.21
392.14	112.1	30.74
394.72	101.2	27.74
395.39	109.3	29.97
397.08	134.4	36.84
402.13	55.7	15.28
408.18	82.8	22.70
421.97	74.7	20.47
434.24	90.7	24.85
438.89	104.0	28.51
461.12	67.1	18.38
469.27	215.7	59.14
472.52	101.5	27.81
473.80	137.1	37.57
495.01	364.8	100.00
508.06	103.5	28.36
518.04	84.5	23.17
520.35	256.2	70.25
524.06	157.3	43.13
528.52	67.5	18.49

533.11	117.7	32.27

RT: 3.59 - 4.13 SM: 15G

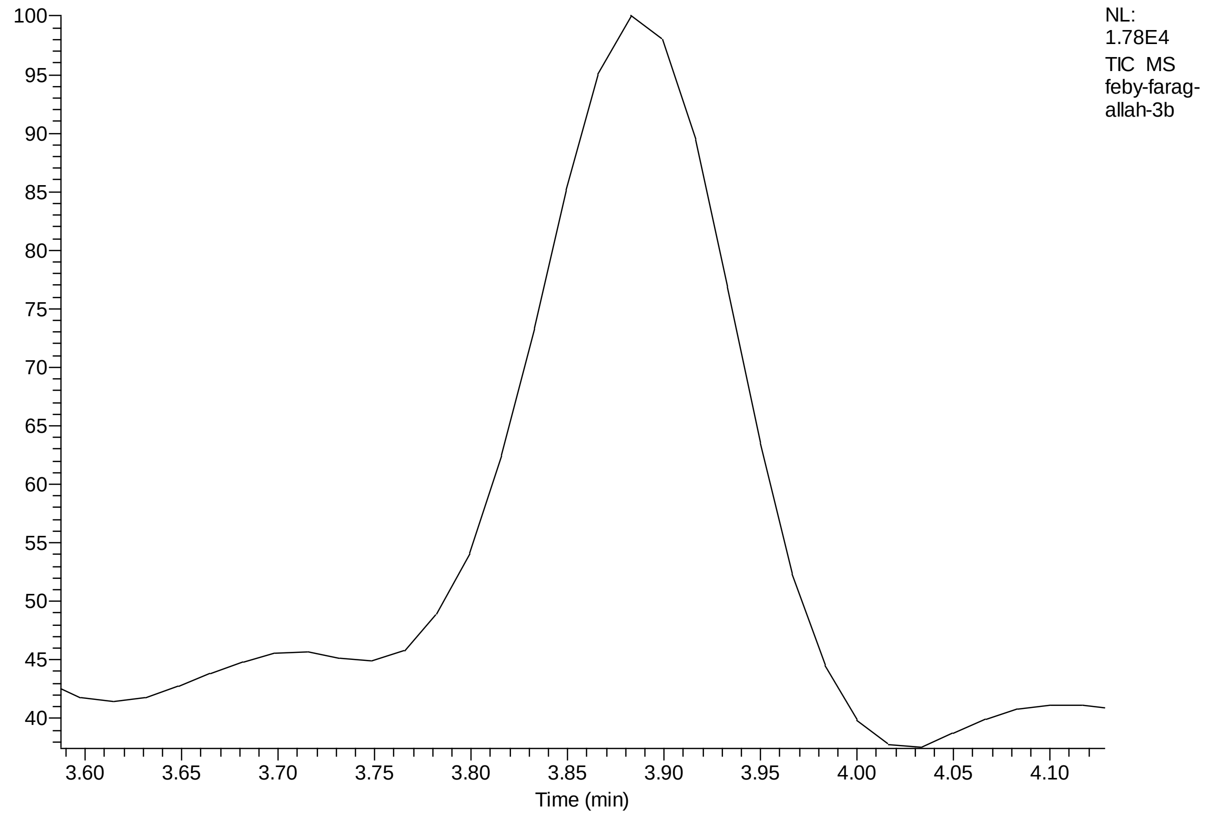

feby-farag-allah-3b #231 RT: 3.88 AV: 1 SB: 2 4.45 , 4.45 NL: 6.21E2
T: {0,0} + c EI Full ms [40.00-1000.00]

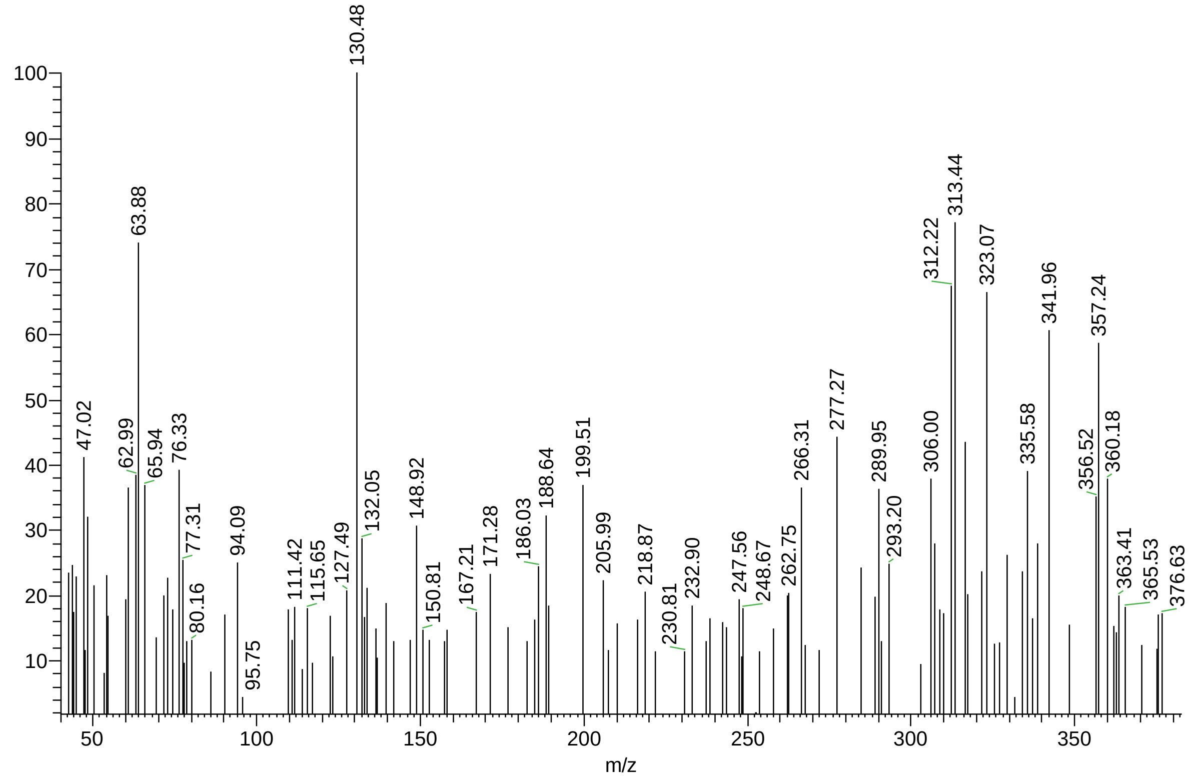

m/z	Intensity	Relative
42.44	145.5	23.43
43.50	153.1	24.65
44.23	108.5	17.48
45.00	142.0	22.87
47.02	255.2	41.10
47.55	71.7	11.55
48.51	198.8	32.02
50.38	133.5	21.49
53.26	50.5	8.14
54.04	143.3	23.08
54.70	104.4	16.81
60.02	120.4	19.39
60.75	227.1	36.57
62.99	239.1	38.50
63.88	459.6	74.02
65.94	229.3	36.93
69.43	83.6	13.46
71.44	123.7	19.93
72.83	141.1	22.72
74.28	110.8	17.84
76.33	243.7	39.25
77.31	158.1	25.47
78.01	60.3	9.71

78.71	80.3	12.93
80.16	81.6	13.14
85.95	51.9	8.35
90.10	105.3	16.96
94.09	155.5	25.04
95.75	26.7	4.29
109.80	110.1	17.74
110.83	82.1	13.23
111.42	112.8	18.17
113.88	53.6	8.63
115.65	111.9	18.02
117.00	60.3	9.71
122.50	104.7	16.86
123.15	66.1	10.65
127.49	128.5	20.70
130.48	620.9	100.00
132.05	178.0	28.67
133.02	103.3	16.64
133.78	131.5	21.17
136.28	92.5	14.90
137.01	64.3	10.35
139.42	117.1	18.85
141.86	80.3	12.93
146.89	81.1	13.06
148.92	190.8	30.73

150.81	91.7	14.77
152.86	82.1	13.23
157.49	80.5	12.97
158.35	91.7	14.77
167.21	108.3	17.44
171.28	144.1	23.21
176.74	93.2	15.01
182.47	80.3	12.93
185.12	100.7	16.21
186.03	152.0	24.48
188.64	200.4	32.27
189.17	114.3	18.40
199.51	228.4	36.78
205.99	138.0	22.22
207.51	71.6	11.53
210.23	97.9	15.76
216.36	101.1	16.28
218.87	127.9	20.59
221.71	70.3	11.32
230.81	70.7	11.38
232.90	114.3	18.40
237.29	80.8	13.01
238.35	102.5	16.51
242.50	98.9	15.93
243.60	93.5	15.05

247.56	120.3	19.37
248.14	65.6	10.56
248.67	111.7	17.99
252.57	12.1	1.95
253.84	71.2	11.47
257.90	91.9	14.79
262.08	123.9	19.95
262.75	125.9	20.27
266.31	226.9	36.55
267.60	76.9	12.39
272.07	72.3	11.64
277.27	274.9	44.28
284.60	150.5	24.24
289.08	122.3	19.69
289.95	225.2	36.27
291.01	80.0	12.88
293.20	154.3	24.84
303.08	58.5	9.43
306.00	235.5	37.92
307.09	173.9	28.00
308.70	110.0	17.72
309.74	106.5	17.16
312.22	418.0	67.32
313.44	478.6	77.09
316.65	269.4	43.40

317.41	125.2	20.16
321.74	146.5	23.60
323.07	412.5	66.44
325.62	77.5	12.48
327.14	78.9	12.71
329.27	163.1	26.26
331.53	27.7	4.47
334.08	146.4	23.58
335.58	242.1	39.00
337.08	101.9	16.41
338.55	172.9	27.85
341.96	376.1	60.58
348.54	96.5	15.55
356.52	218.5	35.19
357.24	364.1	58.64
360.18	234.5	37.77
361.80	95.2	15.33
362.80	88.7	14.28
363.41	124.1	19.99
365.53	113.1	18.21
370.47	76.9	12.39
374.96	73.5	11.83
375.63	105.5	16.99

376.63	106.5	17.16

RT: 2.86 - 3.41 SM: 15G

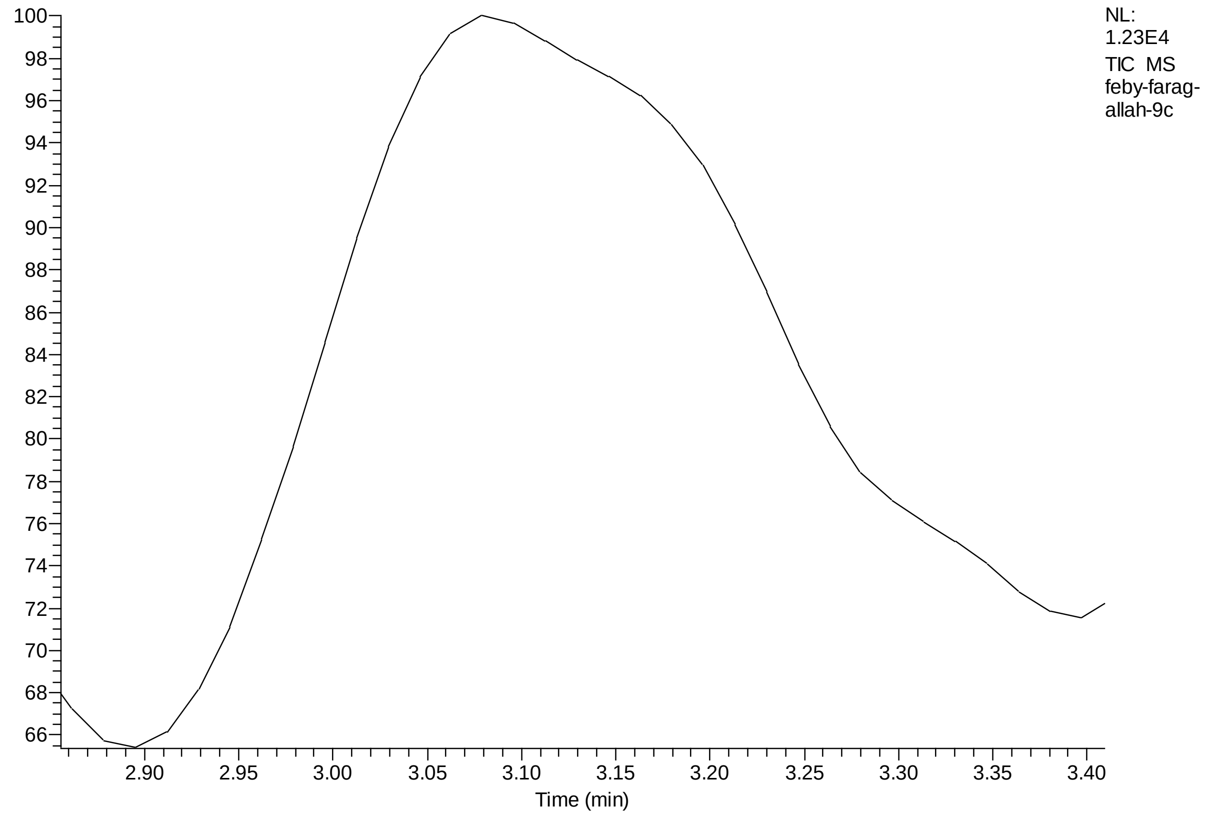

feby-farag-allah-9c #175-177 RT: 2.95-2.98 AV: 3 SB: 2 4.45 , 4.45 NL: 2.41E2
T: {0,0} + c EI Full ms [40.00-1000.00]

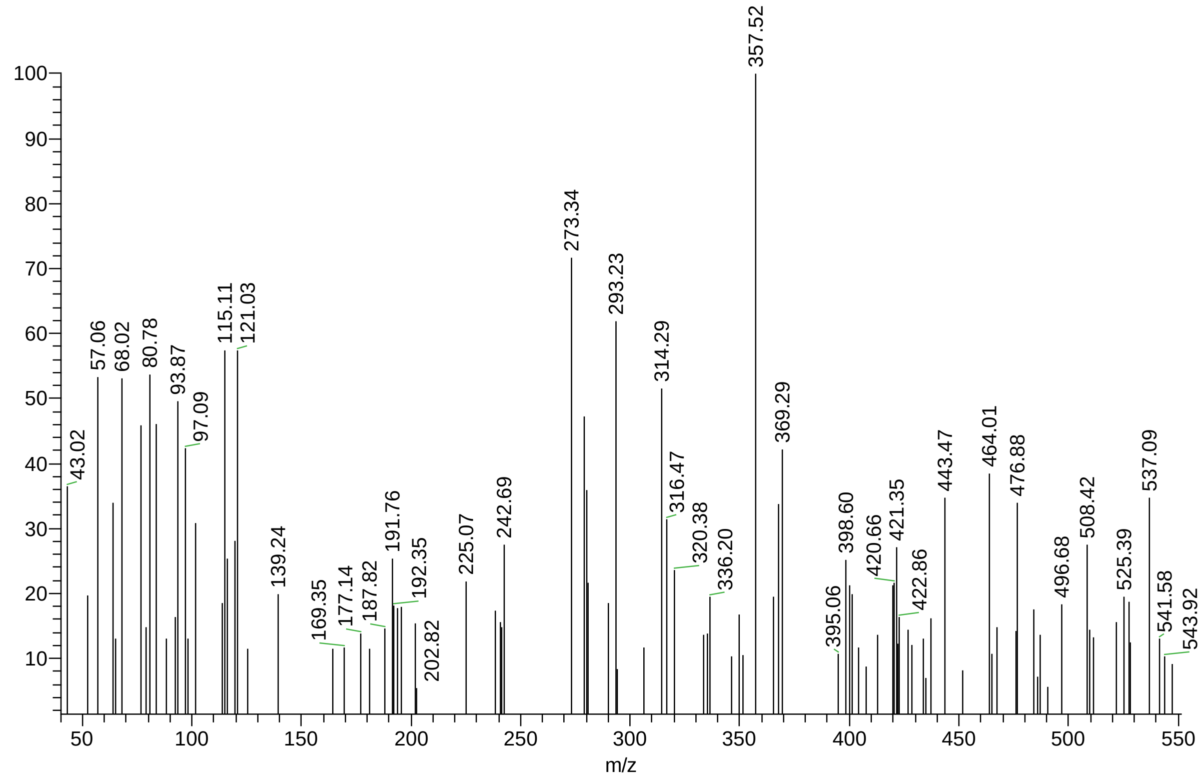

m/z	Intensity	Relative
43.02	87.8	36.47
52.22	47.2	19.59
57.06	127.8	53.11
64.14	81.7	33.94
65.28	31.2	12.98
68.02	127.8	53.09
76.51	110.0	45.72
79.36	35.6	14.79
80.78	129.0	53.61
83.94	110.7	46.02
88.55	31.2	12.96
92.46	39.1	16.23
93.87	118.9	49.42
97.09	101.5	42.20
98.25	31.1	12.93
101.57	74.0	30.77
113.83	44.4	18.43
115.11	138.1	57.38
116.35	60.9	25.30
119.42	67.2	27.90
121.03	137.8	57.25
125.55	27.2	11.30
139.24	47.5	19.72

164.34	27.2	11.28
169.35	28.0	11.63
177.14	32.8	13.63
180.88	27.4	11.38
187.82	35.0	14.55
191.76	60.8	25.26
192.35	43.4	18.04
194.03	42.3	17.56
195.69	43.1	17.91
201.81	36.8	15.31
202.82	12.8	5.34
225.07	52.4	21.77
238.30	41.6	17.29
240.54	37.2	15.46
241.08	35.6	14.77
242.69	66.0	27.42
273.34	172.4	71.63
279.26	113.5	47.17
280.14	86.4	35.90
280.93	52.0	21.59
290.14	44.1	18.32
293.23	148.8	61.85
294.07	19.6	8.16
306.48	27.9	11.58
314.29	123.6	51.36

316.47	75.2	31.23
320.38	56.4	23.42
333.53	32.7	13.59
335.00	33.2	13.81
336.20	46.5	19.34
346.43	24.4	10.14
349.80	40.1	16.68
351.63	25.0	10.38
357.52	240.7	100.00
365.33	46.5	19.32
367.47	80.8	33.57
369.29	101.1	41.99
395.06	25.6	10.62
398.60	60.4	25.12
400.14	50.8	21.13
401.30	47.8	19.85
404.07	27.6	11.49
407.58	20.9	8.70
412.93	32.6	13.55
420.00	51.1	21.22
420.66	51.7	21.50
421.35	64.9	26.98
422.23	29.1	12.10
422.86	39.0	16.21
426.67	34.4	14.29

428.79	28.9	12.00
433.72	31.1	12.93
434.79	16.7	6.94
437.06	38.7	16.08
443.47	83.5	34.72
451.89	19.6	8.13
464.01	92.1	38.26
465.21	25.6	10.64
467.40	35.6	14.77
476.34	34.2	14.20
476.88	81.3	33.78
484.14	41.7	17.34
485.85	16.9	7.02
487.19	32.4	13.46
490.45	13.5	5.60
496.68	44.0	18.30
508.42	66.0	27.44
509.93	34.4	14.28
511.39	31.8	13.22
521.73	37.1	15.40
525.39	46.6	19.37
527.45	44.8	18.61
528.27	29.6	12.32
537.09	83.2	34.57
541.58	31.3	13.00

543.92	24.4	10.12

547.25	21.6	8.99

RT: 4.55 - 4.96 SM: 15G

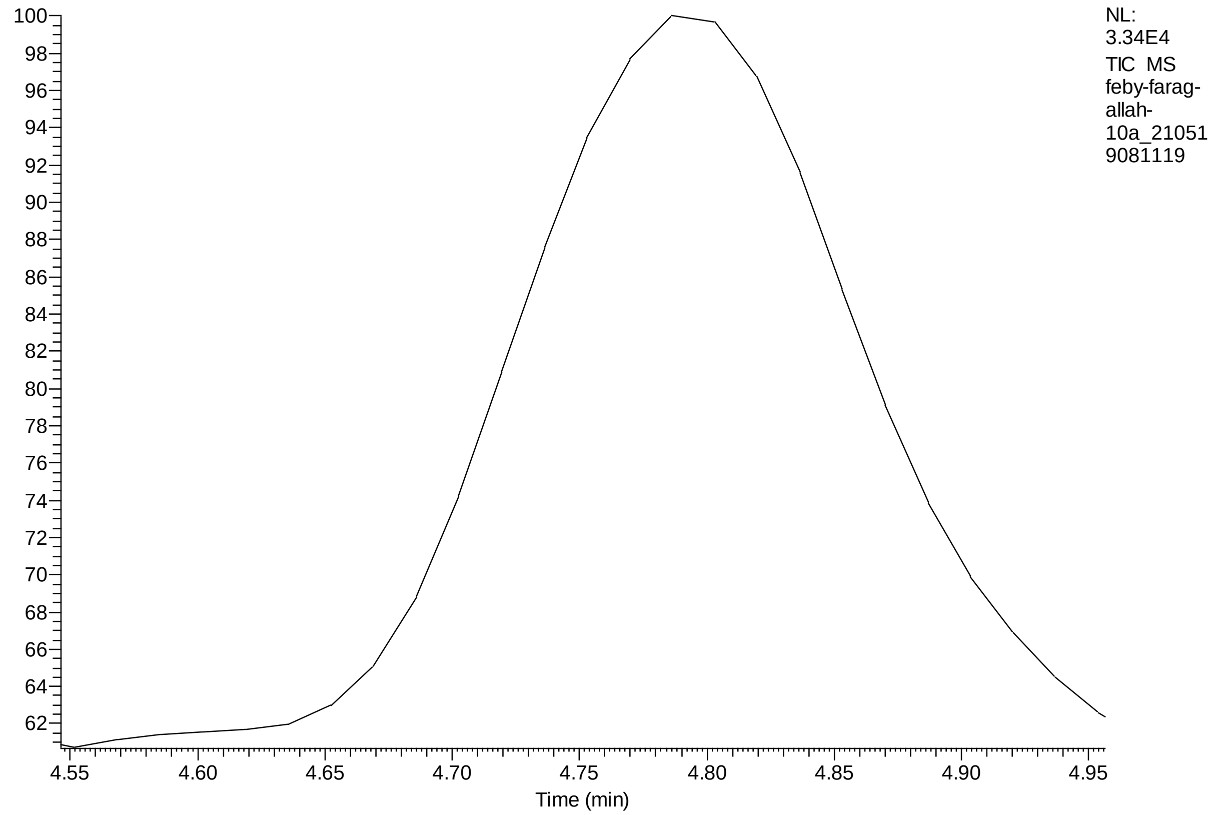

feby-farag-allah-10a_210519081119 #114 RT: 1.92 AV: 1 SB: 2 4.45 , 4.45 NL: 2.61E2
T: {0,0} + c EI Full ms [40.00-1000.00]

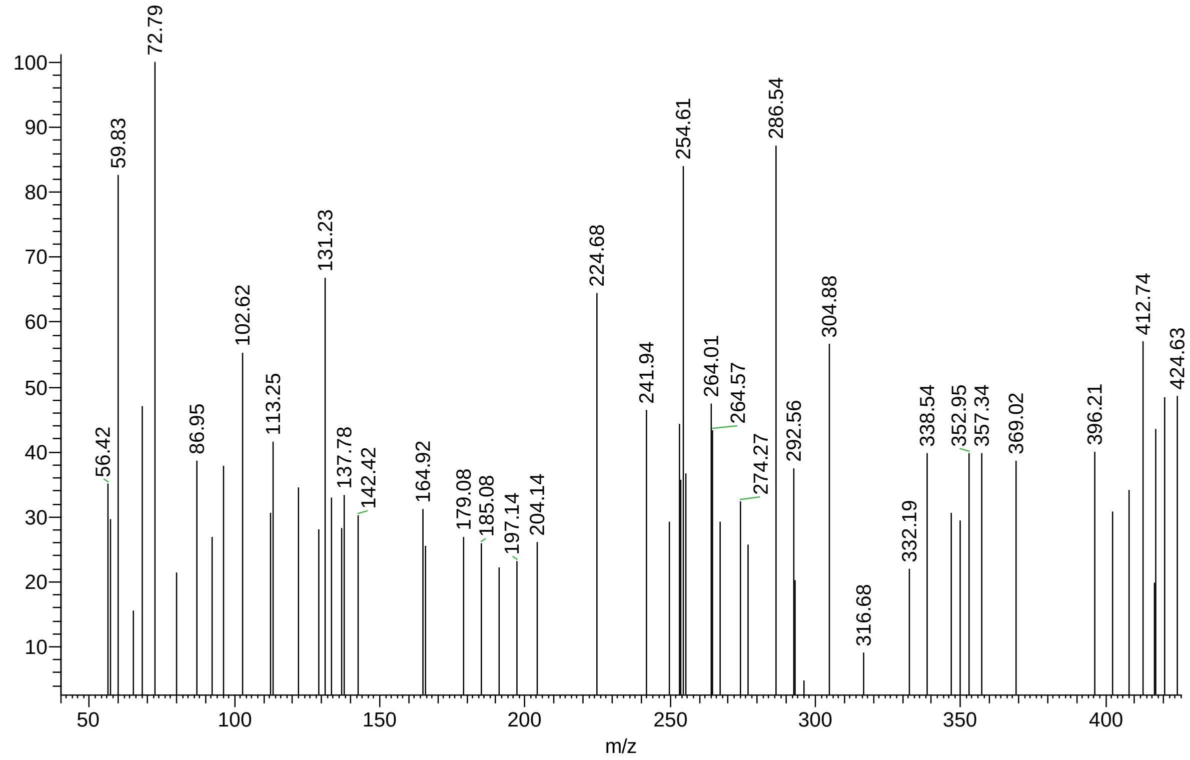

m/z	Intensity	Relative
40.16	221.2	84.90
56.42	91.5	35.11
57.17	76.9	29.53
59.83	215.2	82.60
65.08	40.4	15.51
68.03	122.5	47.03
72.79	260.5	100.00
79.90	55.7	21.39
86.95	100.7	38.64
92.15	69.9	26.82
96.35	98.7	37.87
102.62	143.6	55.12
112.56	79.5	30.50
113.25	108.1	41.50
122.22	90.0	34.54
129.10	73.2	28.10
131.23	173.9	66.73
133.28	85.7	32.91
136.82	73.6	28.25
137.78	86.7	33.27
142.42	78.7	30.19
164.92	81.1	31.12
165.74	66.3	25.44

179.08	69.7	26.77
185.08	67.5	25.90
190.93	57.5	22.06
197.14	60.0	23.03
204.14	68.0	26.10
224.68	167.7	64.38
241.94	121.1	46.47
249.53	76.1	29.22
253.03	115.2	44.22
253.57	92.7	35.57
254.61	218.7	83.93
255.27	95.6	36.69
264.01	123.3	47.34
264.57	112.5	43.19
267.41	76.1	29.22
274.27	84.3	32.34
276.87	67.1	25.74
286.54	226.9	87.10
292.56	97.3	37.36
293.10	52.4	20.11
296.10	12.4	4.76
304.88	147.2	56.50
316.68	23.6	9.06
332.19	57.2	21.95
338.54	103.5	39.71

346.91	79.6	30.55
350.02	76.4	29.32
352.95	103.5	39.71
357.34	103.7	39.82
369.02	100.5	38.59
396.21	104.1	39.97
402.20	80.1	30.76
408.23	88.9	34.14
412.74	148.4	56.96
416.86	51.6	19.81
417.43	113.2	43.45
420.20	126.1	48.41

424.63	126.4	48.52

RT: 2.99 - 4.09 SM: 15G

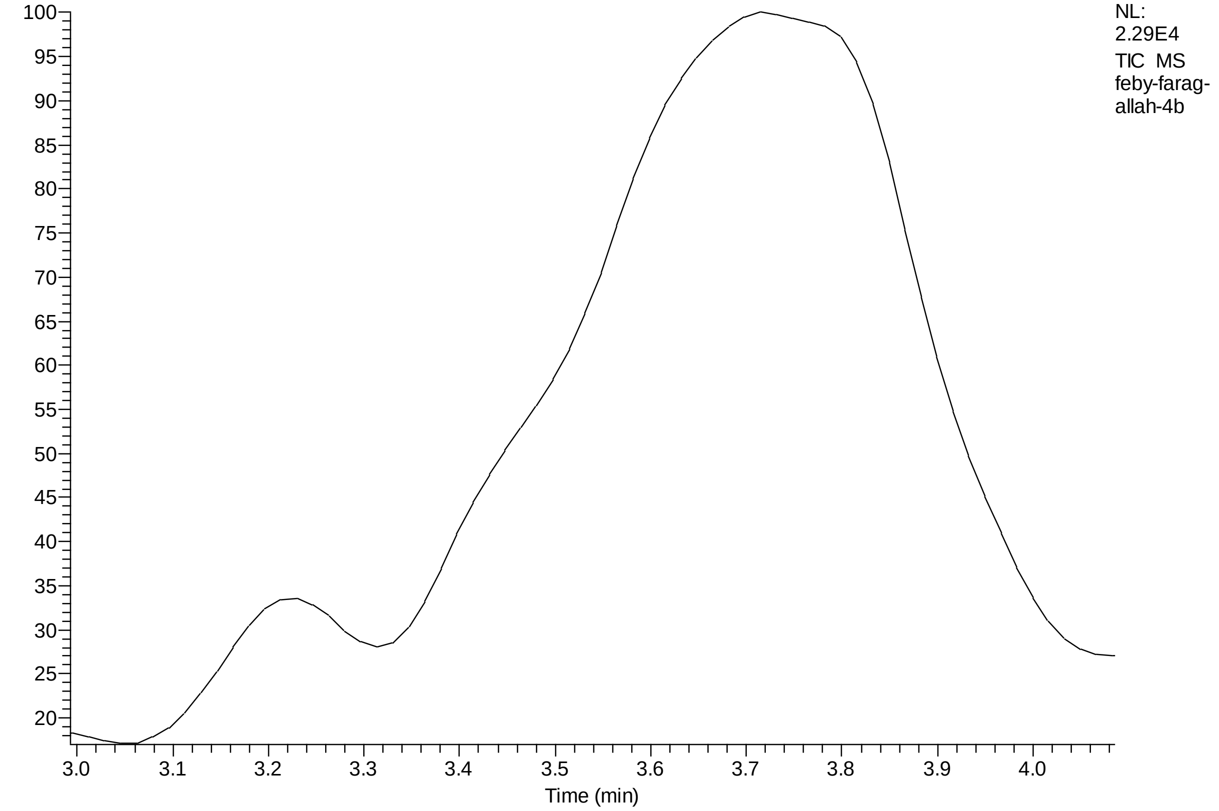

feby-farag-allah-4b #216 RT: 3.63 AV: 1 SB: 2 4.45 , 4.45 NL: 5.41E2
T: {0,0} + c EI Full ms [40.00-1000.00]

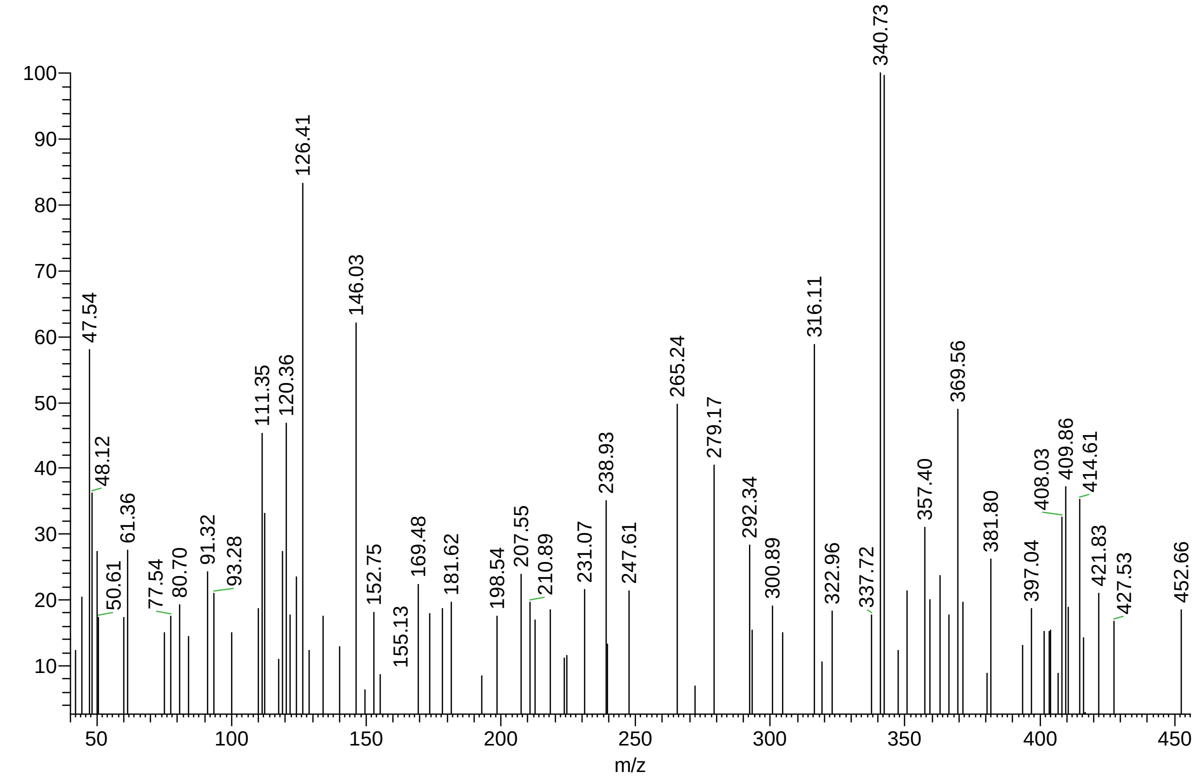

m/z	Intensity	Relative
42.20	66.3	12.26
44.45	110.3	20.39
47.54	313.8	58.05
48.12	196.0	36.25
49.91	148.0	27.37
50.61	93.3	17.26
60.09	94.0	17.39
61.36	149.3	27.62
75.29	81.5	15.07
77.54	94.4	17.46
80.70	103.7	19.19
83.82	78.0	14.43
91.32	131.5	24.32
93.28	113.1	20.91
100.26	80.7	14.92
109.79	100.7	18.62
111.35	245.1	45.33
112.53	179.6	33.22
117.61	58.8	10.88
118.96	147.9	27.35
120.36	252.8	46.76
121.78	95.2	17.61
123.96	126.7	23.43

126.41	450.2	83.28
128.95	66.1	12.23
133.91	94.5	17.48
140.15	70.0	12.95
146.03	335.2	62.00
149.75	33.6	6.21
152.75	98.3	18.18
155.13	46.1	8.53
169.48	120.8	22.34
173.41	96.8	17.90
178.39	100.8	18.64
181.62	105.6	19.53
192.94	45.1	8.34
198.54	94.7	17.51
207.55	129.1	23.87
210.89	106.4	19.68
212.88	92.0	17.02
218.57	99.3	18.37
223.40	60.7	11.22
224.27	62.0	11.47
231.07	116.7	21.58
238.93	190.0	35.14
239.61	71.3	13.19
247.61	116.0	21.45
265.24	268.8	49.72

272.01	37.6	6.95
279.17	218.8	40.47
292.34	153.3	28.36
293.07	82.8	15.31
300.89	102.7	18.99
304.72	80.8	14.94
316.11	317.6	58.74
319.40	56.8	10.51
322.96	98.4	18.20
337.72	95.5	17.66
340.73	540.6	100.00
342.40	539.3	99.75
347.64	66.0	12.21
350.80	115.5	21.36
357.40	167.2	30.92
359.44	108.1	20.00
362.94	128.4	23.75
366.06	95.9	17.73
369.56	264.5	48.93
371.35	105.9	19.58
380.46	47.3	8.75
381.80	141.5	26.17
393.73	71.1	13.14
397.04	100.8	18.64
401.74	82.1	15.19

403.41	81.9	15.14
404.21	83.1	15.36
406.86	48.0	8.88
408.03	176.1	32.58
409.86	200.9	37.16
410.39	102.4	18.94
414.61	190.7	35.27
416.06	77.1	14.25
416.69	14.8	2.74
421.83	113.9	21.06
427.53	90.7	16.77

452.66	99.3	18.37

RT: 1.49 - 1.94 SM: 15G

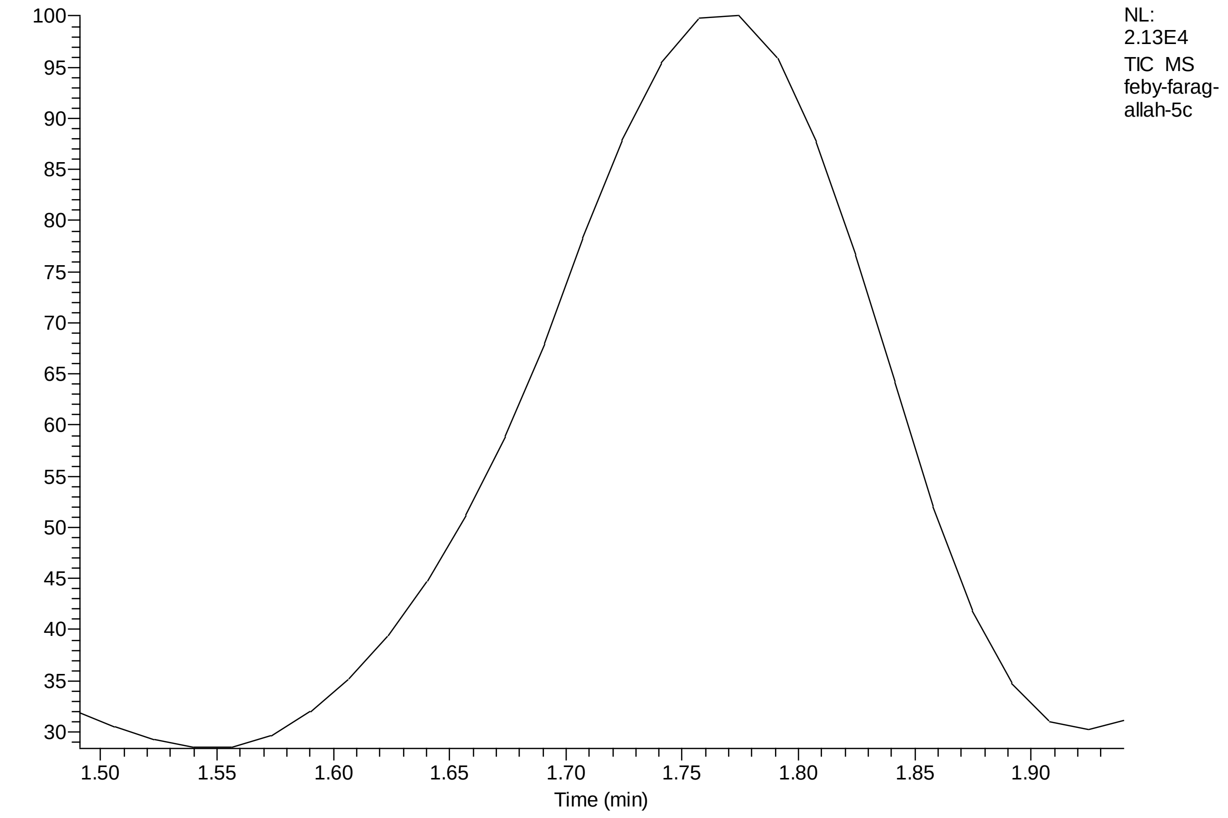

feby-farag-allah-5c #105 RT: 1.77 AV: 1 SB: 2 4.45 , 4.45 NL: 3.75E2
T: {0,0} + c EI Full ms [40.00-1000.00]

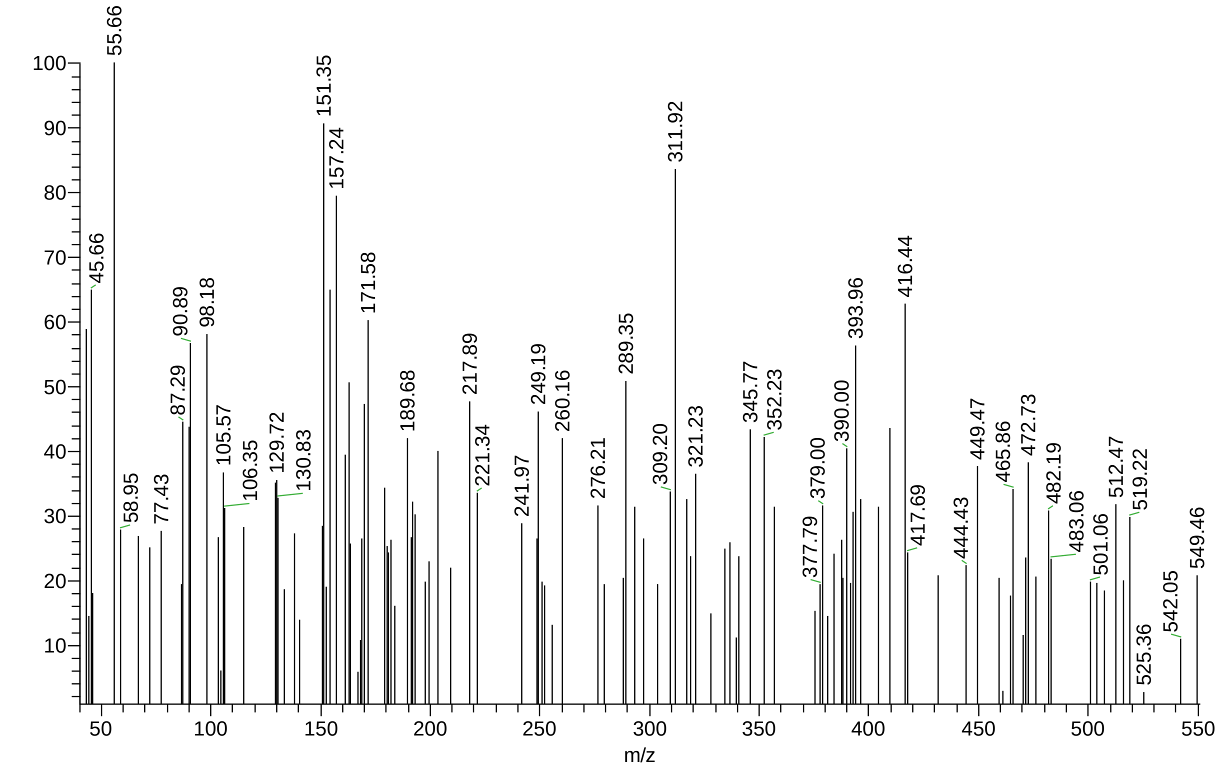

m/z	Intensity	Relative
40.21	172.7	46.00
43.17	220.7	58.79
44.37	54.1	14.42
45.66	243.9	64.97
46.35	67.9	18.08
55.66	375.3	100.00
58.95	104.1	27.74
67.16	100.5	26.79
72.36	94.0	25.04
77.43	103.5	27.57
86.71	72.8	19.40
87.29	167.3	44.58
90.31	164.3	43.77
90.89	212.5	56.63
98.18	217.6	57.98
103.50	99.7	26.57
104.25	22.4	5.97
105.57	137.3	36.59
106.35	116.8	31.12
115.02	106.0	28.24
129.18	131.9	35.13
129.72	133.5	35.56
130.83	122.7	32.68

133.25	70.0	18.65
138.28	102.0	27.18
140.37	52.4	13.96
150.68	106.3	28.31
151.35	340.6	90.76
152.55	71.1	18.93
154.40	243.6	64.90
157.24	298.5	79.54
161.51	148.0	39.43
162.94	189.9	50.59
163.70	96.3	25.65
167.32	22.0	5.86
167.95	40.1	10.69
168.54	99.5	26.50
169.99	177.2	47.21
171.58	226.1	60.25
179.01	128.7	34.28
180.22	95.1	25.33
180.75	91.5	24.37
182.09	98.5	26.25
183.94	60.1	16.02
189.68	157.6	41.99
191.22	99.7	26.57
192.15	120.3	32.04
192.94	112.9	30.09

197.75	74.3	19.79
199.64	86.1	22.95
203.39	150.1	40.00
209.31	82.4	21.95
217.89	178.8	47.64
221.34	125.5	33.43
241.97	107.7	28.70
248.68	99.5	26.50
249.19	173.1	46.11
250.89	74.5	19.86
252.40	71.6	19.08
255.88	49.5	13.18
260.16	157.5	41.95
276.21	118.5	31.58
279.59	72.8	19.40
287.74	76.3	20.32
289.35	190.5	50.76
293.34	117.5	31.30
297.35	98.9	26.36
303.66	72.7	19.36
309.20	126.8	33.78
311.92	314.0	83.66
316.95	122.4	32.61
318.40	89.1	23.73
321.23	136.8	36.45

328.20	55.7	14.85
334.40	93.3	24.87
336.40	97.1	25.86
339.58	41.5	11.05
340.86	88.8	23.66
345.77	162.7	43.34
352.23	158.5	42.24
357.14	117.9	31.40
375.26	57.1	15.20
377.79	72.7	19.36
379.00	118.1	31.47
381.26	54.3	14.46
384.27	90.1	24.01
387.35	98.5	26.25
388.07	76.4	20.36
390.00	151.3	40.32
391.47	73.7	19.64
392.73	114.5	30.52
393.96	211.6	56.38
396.41	122.3	32.58
404.33	117.7	31.37
409.78	163.2	43.48
416.44	235.5	62.74
417.69	91.2	24.30
431.37	77.6	20.67

444.43	83.6	22.27
449.47	141.5	37.69
459.53	76.4	20.36
461.22	10.5	2.81
464.63	66.0	17.58
465.86	128.3	34.17
470.30	43.5	11.58
471.62	88.0	23.45
472.73	143.5	38.22
476.07	77.3	20.60
482.19	115.5	30.76
483.06	87.2	23.23
501.06	74.5	19.86
504.18	73.3	19.54
507.22	69.2	18.44
512.47	119.1	31.72
516.15	74.7	19.89
519.22	111.5	29.70
525.36	9.9	2.63
542.05	40.7	10.83

549.46	77.9	20.75

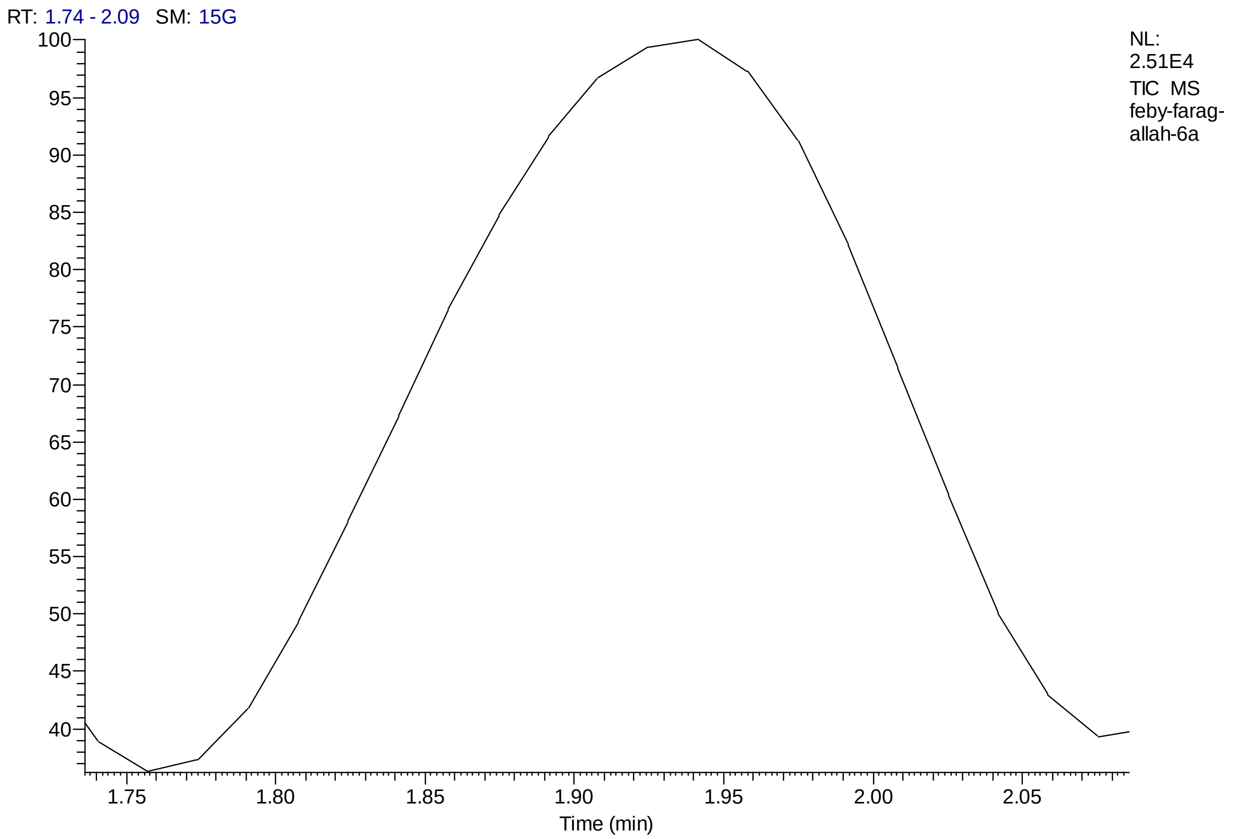
RT: 1.74 - 2.09 SM: 15G
NL: 2.51E4
TIC MS feby-farag-allah-6a
Time (min)

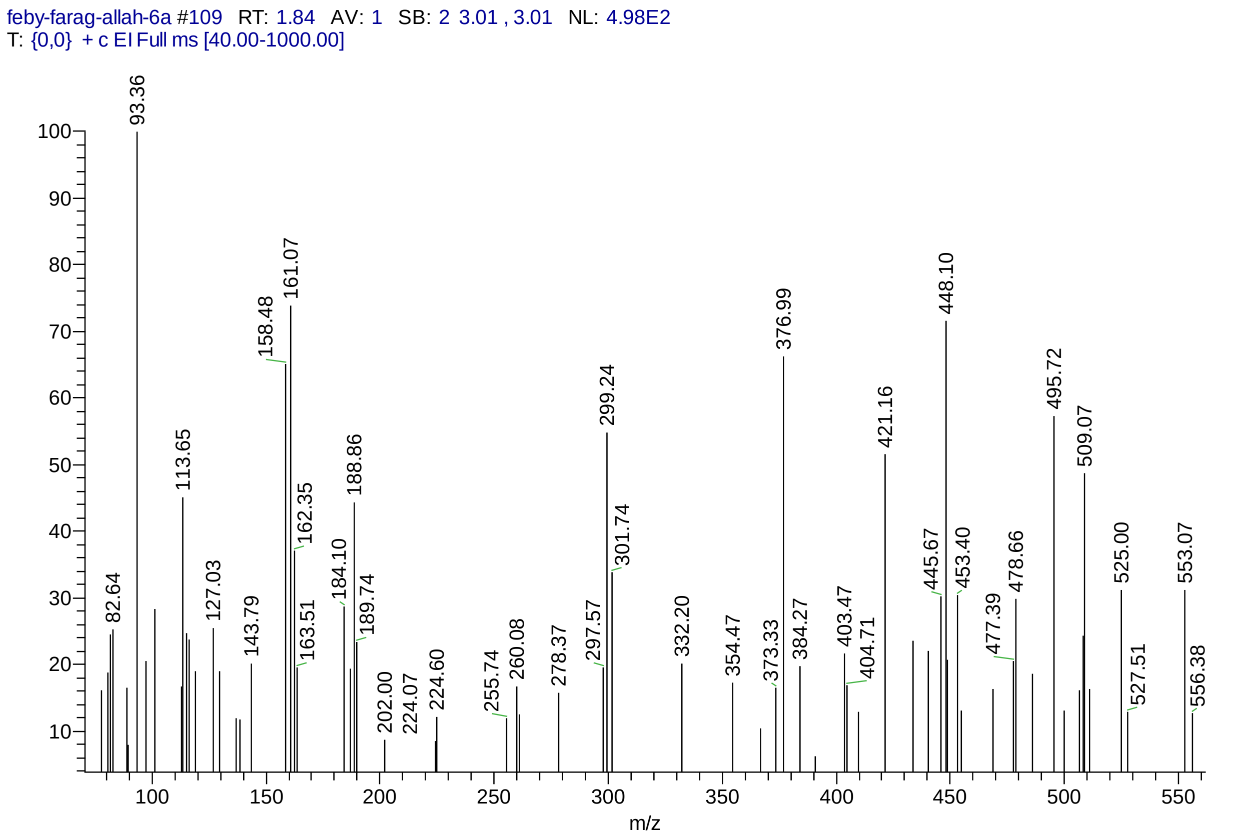
feby-farag-allah-6a #109 RT: 1.84 AV: 1 SB: 2 3.01 , 3.01 NL: 4.98E2
T: {0,0} + c EI Full ms [40.00-1000.00]
93.36
82.64
113.65
127.03
143.79
158.48
161.07
162.35
163.51
184.10
188.86
189.74
202.00
224.07
224.60
255.74
260.08
278.37
297.57
299.24
301.74
332.20
354.47
373.33
376.99
384.27
403.47
404.71
421.16
445.67
448.10
453.40
477.39
478.66
495.72
509.07
525.00
527.51
553.07
556.38
m/z

m/z	Intensity	Relative
78.04	79.9	16.03
80.38	93.5	18.76
81.59	121.9	24.46
82.64	125.5	25.19
88.84	82.0	16.46
89.43	38.9	7.82
93.36	498.1	100.00
97.17	102.1	20.50
101.44	140.4	28.19
112.82	83.1	16.68
113.65	224.3	45.02
115.22	122.4	24.57
116.15	118.1	23.72
119.21	94.7	19.00
127.03	126.7	25.43
129.64	94.4	18.95
137.03	59.3	11.91
138.44	58.1	11.67
143.79	100.4	20.16
158.48	323.7	64.99
161.07	367.4	73.77
162.35	184.9	37.13
163.51	97.3	19.54

184.10	142.5	28.61
187.15	96.0	19.27
188.86	220.4	44.25
189.74	115.9	23.26
202.00	43.5	8.73
224.07	42.4	8.51
224.60	60.7	12.18
255.74	59.3	11.91
260.08	83.1	16.68
261.16	61.7	12.39
278.37	78.3	15.71
297.57	97.1	19.49
299.24	272.9	54.79
301.74	168.1	33.75
332.20	99.9	20.05
354.47	86.1	17.29
366.97	51.9	10.41
373.33	81.9	16.43
376.99	329.7	66.19
384.27	98.4	19.75
390.58	30.5	6.13
403.47	108.0	21.68
404.71	84.0	16.86
409.67	63.7	12.79
421.16	256.4	51.47

433.85	117.2	23.53
440.27	110.0	22.08
445.67	150.7	30.25
448.10	356.0	71.47
448.86	102.7	20.61
453.40	151.6	30.43
455.07	65.3	13.12
468.85	81.1	16.27
477.39	101.6	20.40
478.66	148.5	29.82
486.19	92.7	18.60
495.72	284.6	57.15
500.00	64.5	12.96
506.58	80.4	16.14
508.32	120.9	24.28
509.07	242.4	48.66
510.95	80.9	16.25
525.00	155.1	31.13
527.51	63.7	12.79
553.07	155.2	31.16
556.38	63.5	12.74
